# Supplementary material for: Kinase Inhibitory Activities and Molecular Docking of a Novel Series of Anticancer Pyrazole Derivatives
Source: Molecules. 2018 Nov 24;23(12):3074. doi: 10.3390/molecules23123074 (PMC6321587; doi:10.3390/molecules23123074)

Supplementary material

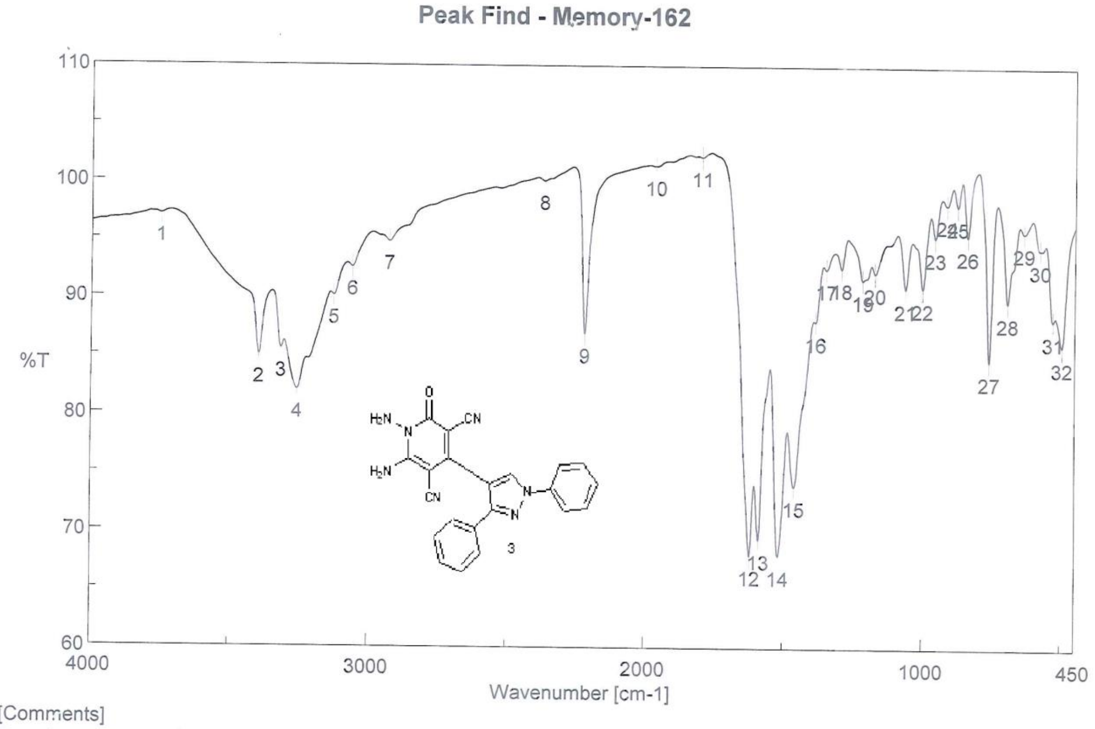

[Comments]

Sample name	V1
Comment	21/4/2016
User	IR
Division	IR
Company	MAC

[Result of Peak Picking]

No.	Position	Intensity	No.	Position	Intensity	No.	Position	Intensity
1	3747.98	97.0565	2	3393.14	84.9864	3	3315.03	85.5201
4	3256.22	82.0298	5	3125.08	90.0806	6	3058.55	92.54
7	2925.48	94.7572	8	2365.26	100.037	9	2217.74	86.873
10	1964.14	101.342	11	1796.37	102.136	12	1620.88	67.9284
13	1589.06	69.2981	14	1519.63	67.9184	15	1462.74	73.8798
16	1386.57	87.9578	17	1348.96	92.4886	18	1294.97	92.5656
19	1217.83	91.5481	20	1173.47	92.1635	21	1063.55	90.818
22	1001.84	90.8775	23	956.52	95.2485	24	914.093	98.0922
25	875.524	97.9658	26	838.883	95.3726	27	758.852	84.6902
28	693.284	89.6731	29	635.43	95.7189	30	576.612	94.2598
31	529.364	88.1336	32	496.58	85.9436			

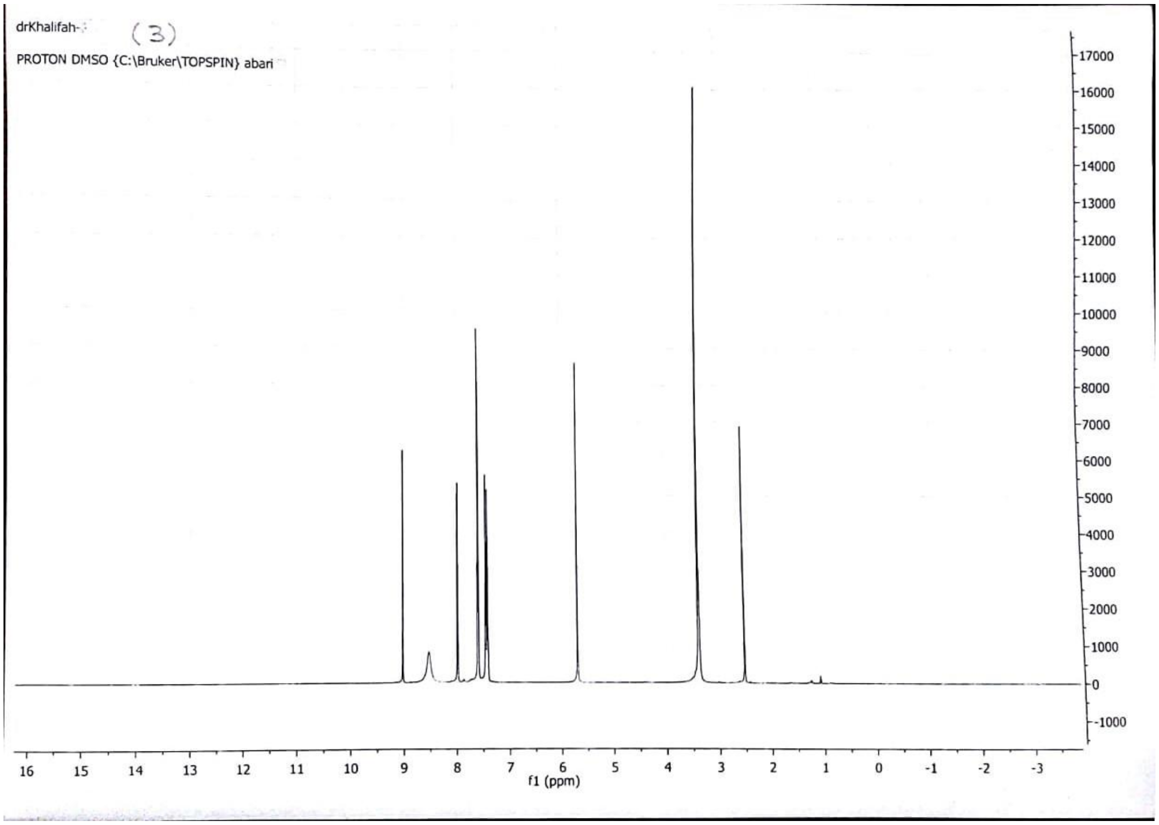
drKhalifah-
(3)
PROTON DMSO {C:\Bruker\TOPSPIN} abari
17000
16000
15000
14000
13000
12000
11000
10000
9000
8000
7000
6000
5000
4000
3000
2000
1000
0
-1000
16 15 14 13 12 11 10 9 8 7 6 5 4 3 2 1 0 -1 -2 -3
f1 (ppm)

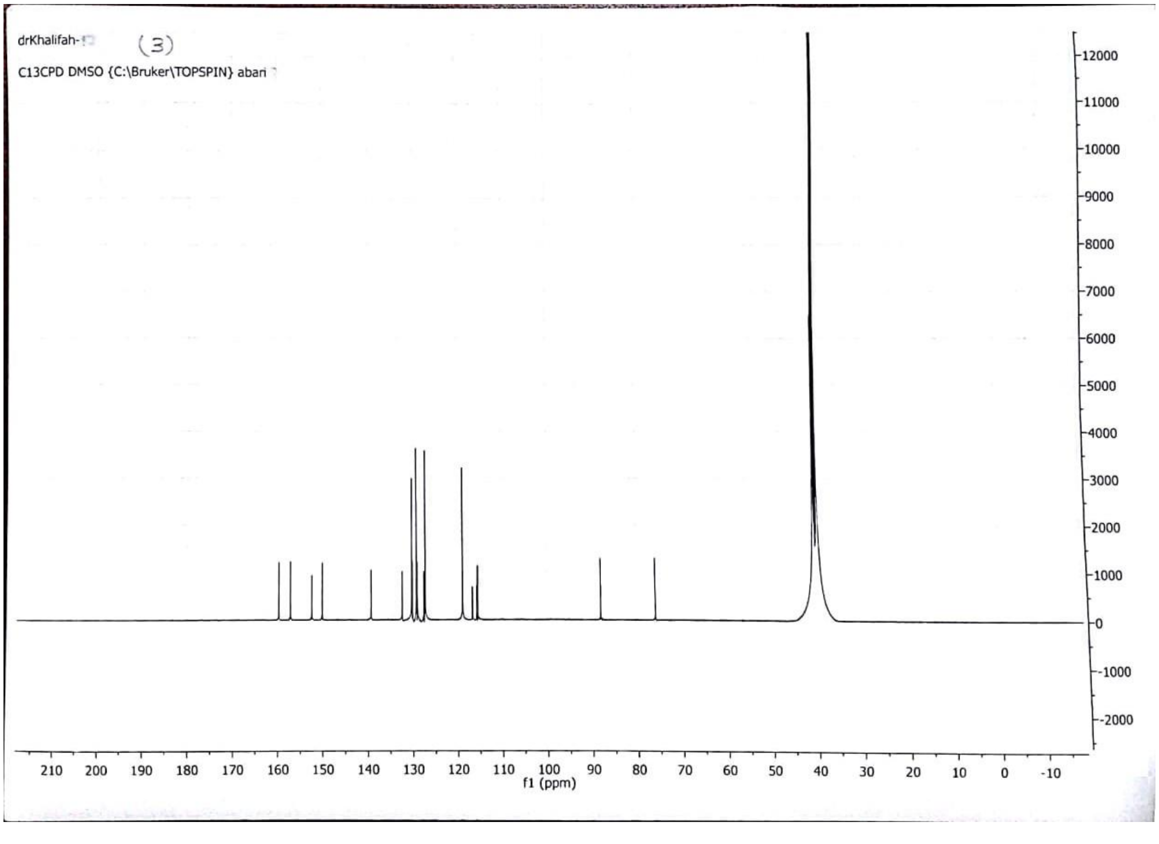
drKhalifah-
(3)
C13CPD DMSO {C:\Bruker\TOPSPIN} abari
12000
11000
10000
9000
8000
7000
6000
5000
4000
3000
2000
1000
0
-1000
-2000
210 200 190 180 170 160 150 140 130 120 110 100 90 80 70 60 50 40 30 20 10 0 -10
f1 (ppm)

Al-Azhar University
The Regional Center for Mycology & Biotechnology
C:\Xcalibur\data\S\eman-samir-v1

2/15/2016 1:32:34 PM

C:\Xcalibur\methods\Mass DIP.meth

RT: 0.60 - 4.36 SM: 15B

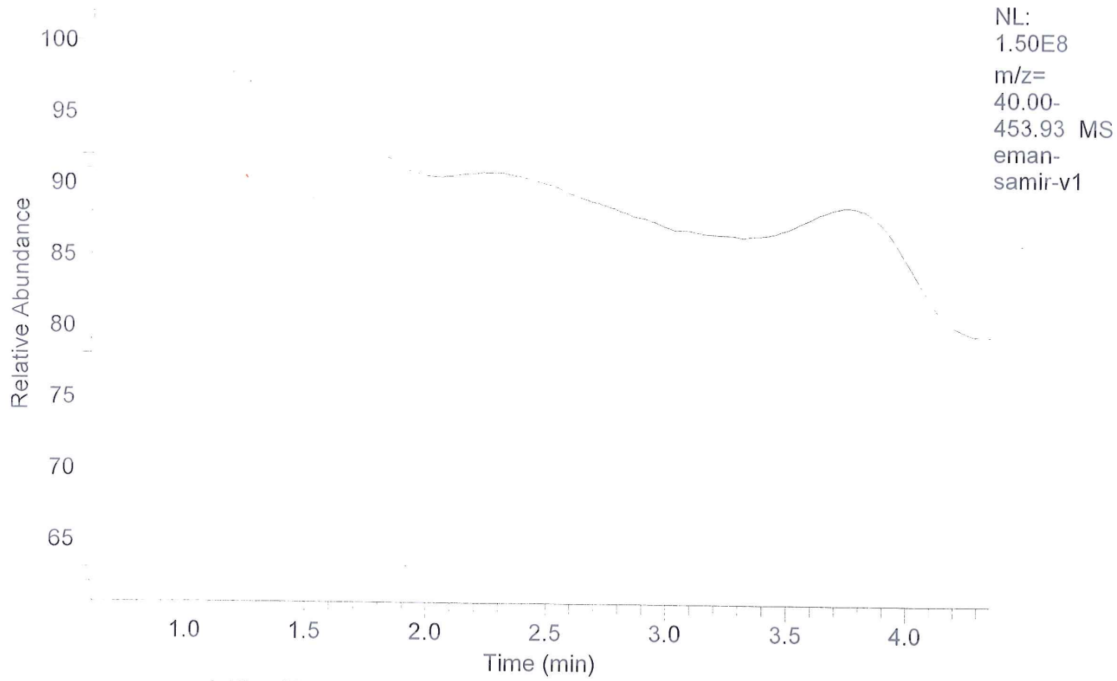

eman-samir-v1 #341 RT: 5.72 AV: 1 NL: 3.64E7
T: {0,0} + c EI Full ms [40.00-1000.00]

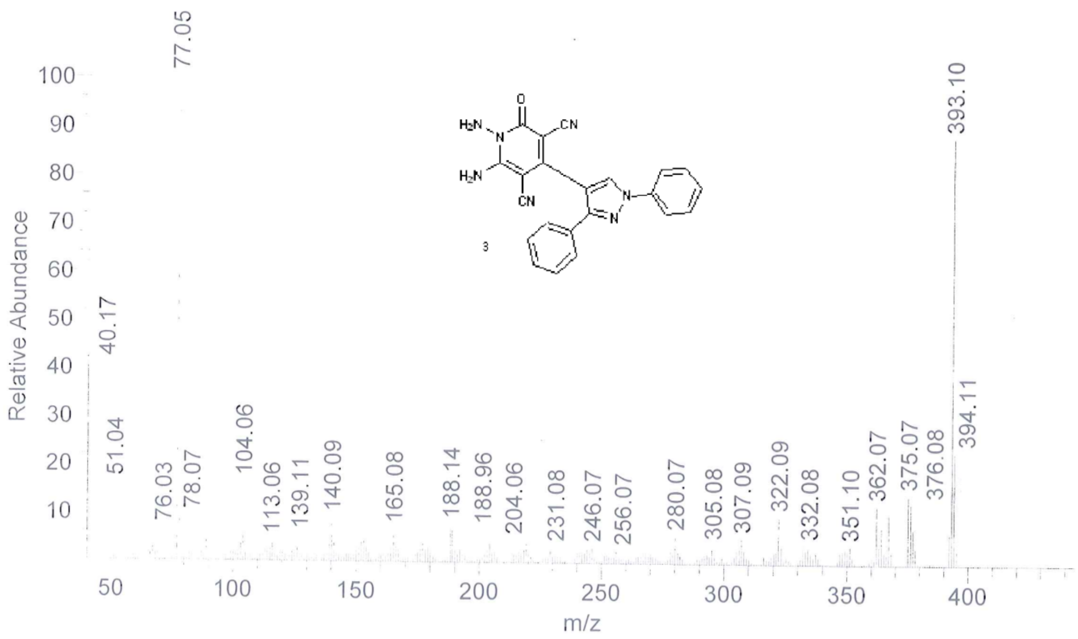

Peak Find - Memory-86

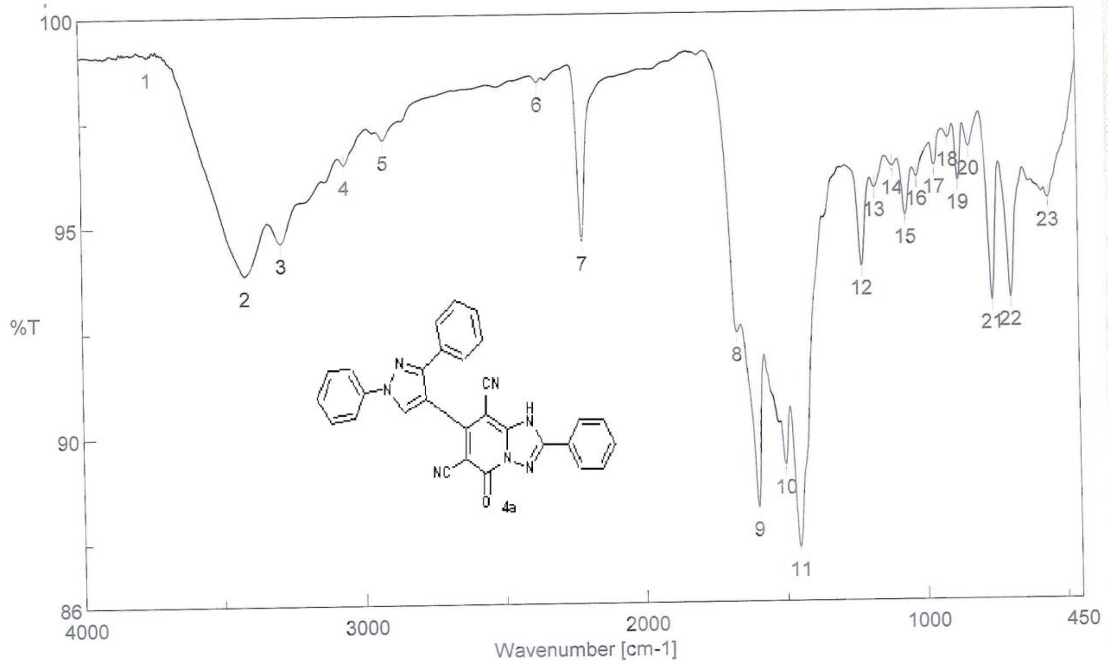

[Comments]

Sample name	V6
Comment	21/4/2016
User	IR
Division	IR
Company	MAC

[Result of Peak Picking]

No.	Position	Intensity	No.	Position	Intensity	No.	Position	Intensity
1	3759.55	99.0883	2	3420.14	93.8496	3	3290.93	94.6125
4	3064.33	96.457	5	2925.48	97.0357	6	2369.12	98.3889
7	2216.77	94.5853	8	1669.09	92.3884	9	1598.7	88.2264
10	1501.31	89.2323	11	1454.06	87.265	12	1222.65	93.926
13	1174.44	95.7825	14	1108.87	96.293	15	1064.51	95.1287
16	1023.05	96.0274	17	959.412	96.3077	18	910.236	96.9516
19	875.524	95.9337	20	835.99	96.7327	21	755.959	93.0871
22	690.391	93.1489	23	555.398	95.4864			

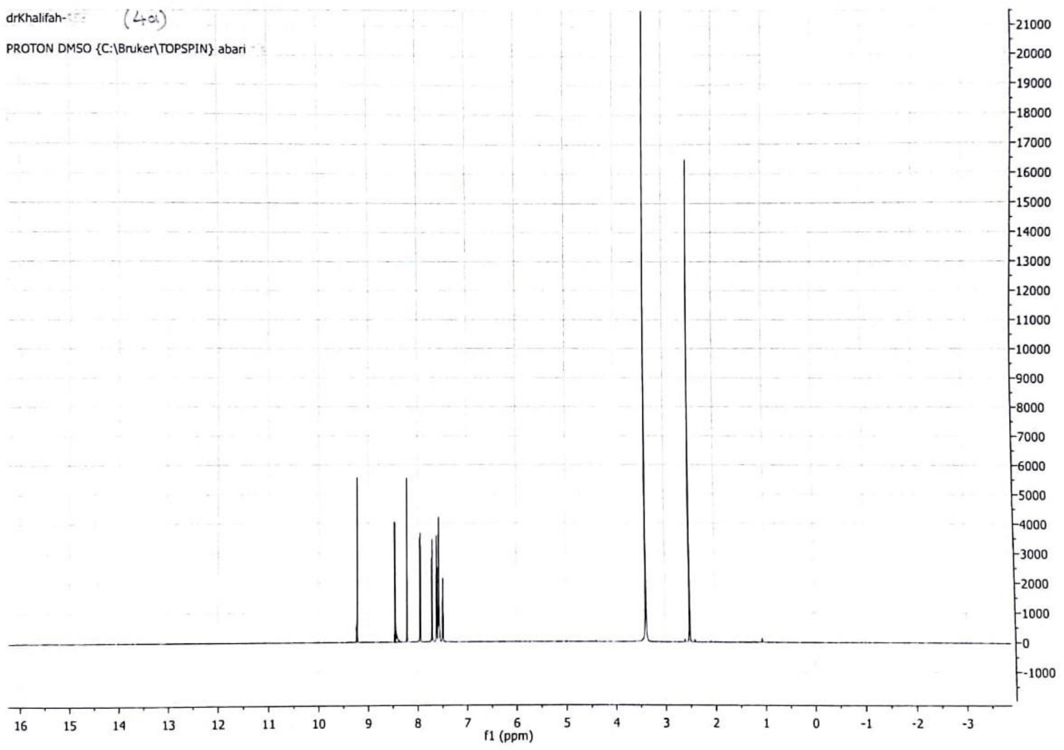
drKhalifah-
(4a)
PROTON DMSO {C:\Bruker\TOPSPIN} abari
f1 (ppm)

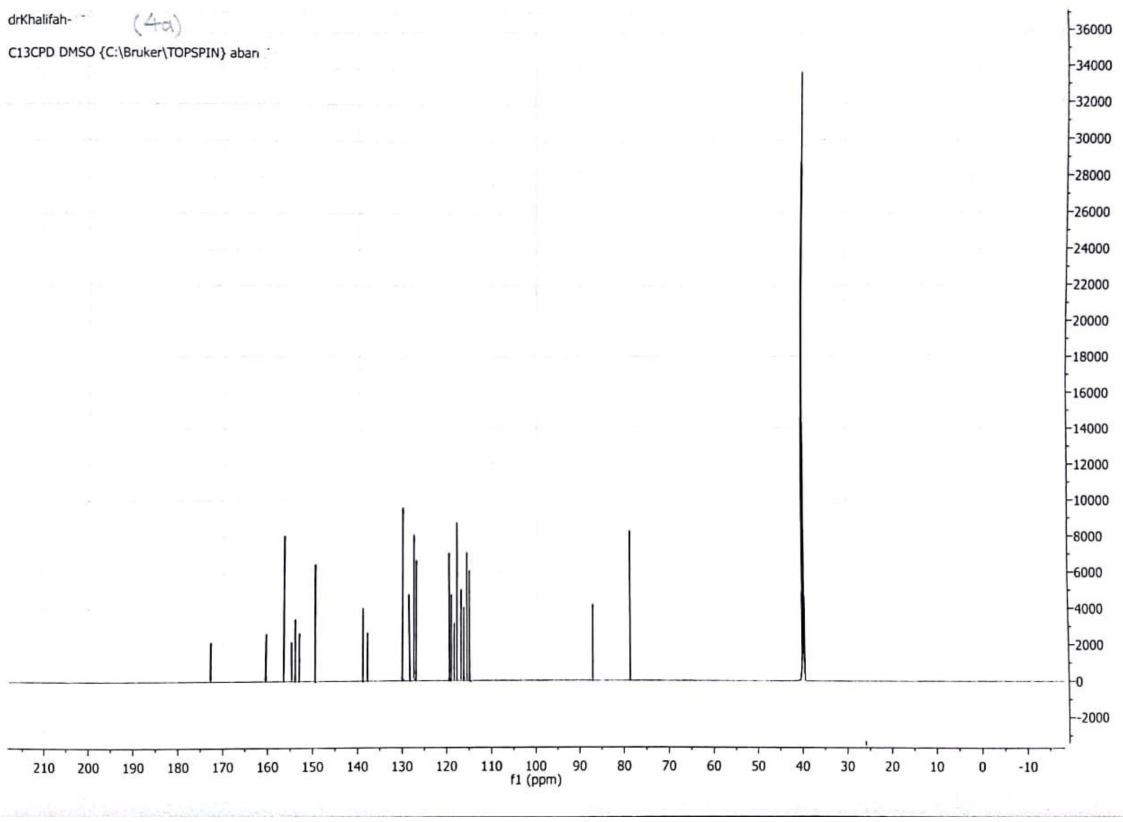
drKhalifah-
(4a)
C13CPD DMSO {C:\Bruker\TOPSPIN} aban
f1 (ppm)

Al-Azhar University
The Regional Center for Mycology & Biotechnology
C:\Xcalibur\data\S\EMAN-SAMIR-V6

2/16/2016 10:11:27 AM
C:\Xcalibur\methods\Mass DIP.meth

RT: 3.43 - 5.98 SM: 15B

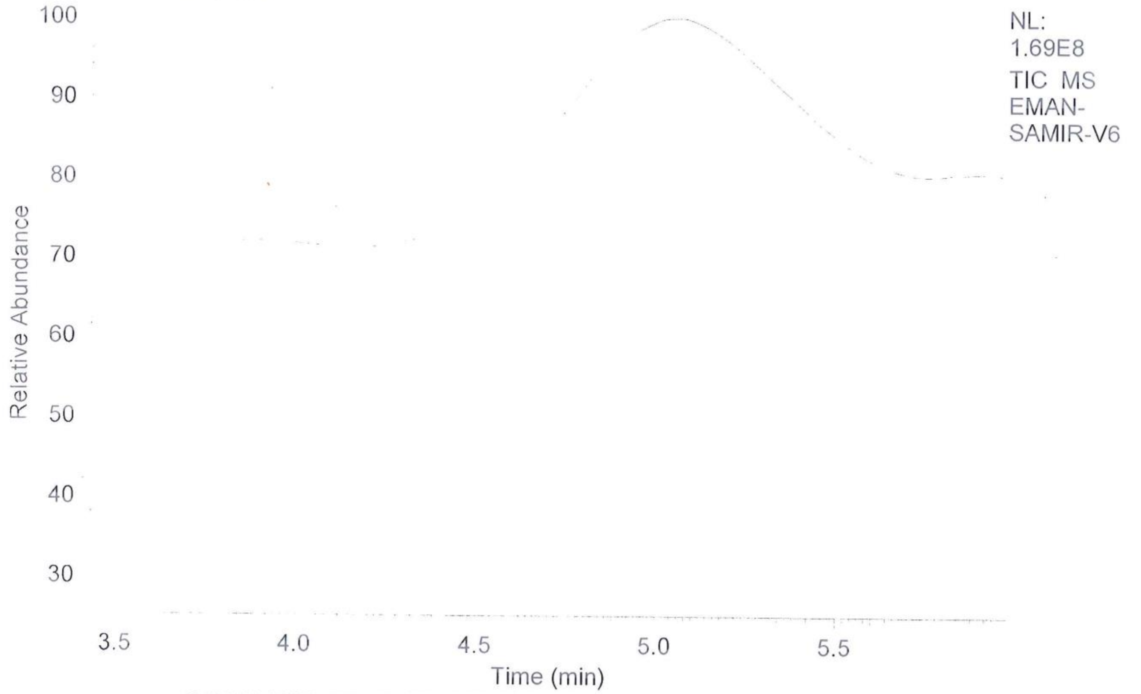

EMAN-SAMIR-V6 #336 RT: 5.64 AV: 1 SB: 10 4.15-4.28 , 4.55 NL: 2.79E6
T: {0,0} + c EI Full ms [40.00-1000.00]

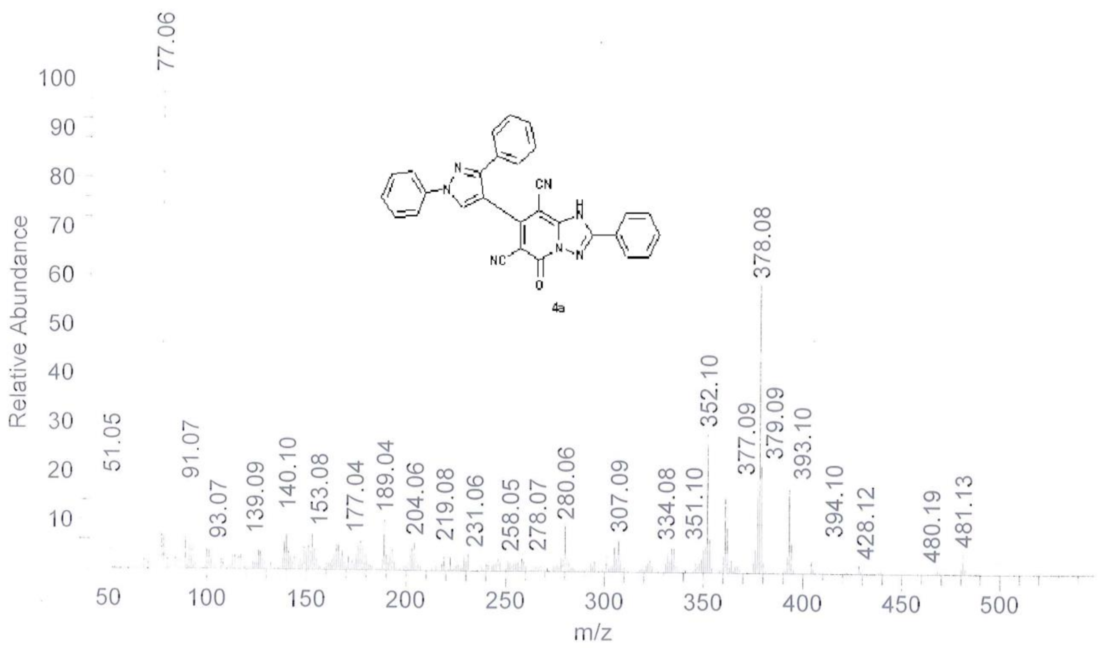

Peak Find - Memory-79

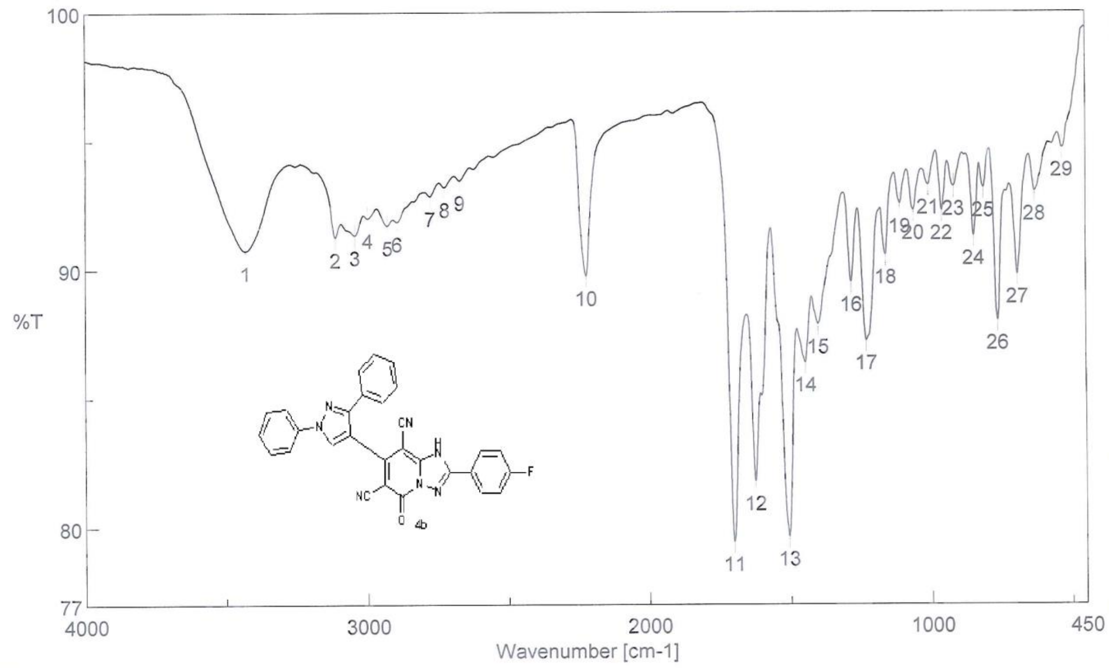

[Comments]

Sample name	V8
Comment	21/4/2016
User	IR
Division	IR
Company	MAC

[Result of Peak Picking]

No.	Position	Intensity	No.	Position	Intensity	No.	Position	Intensity
1	3431.71	90.7308	2	3113.51	91.2383	3	3044.09	91.3233
4	2998.77	91.9883	5	2928.38	91.6995	6	2893.66	91.8429
7	2776.03	92.8299	8	2724.92	93.1782	9	2670.93	93.4424
10	2221.59	89.7271	11	1700.91	79.4129	12	1625.7	81.7832
13	1507.1	79.6191	14	1447.31	86.3816	15	1402	87.8605
16	1284.36	89.4913	17	1231.33	87.2354	18	1161.9	90.5451
19	1110.8	92.5073	20	1062.59	92.261	21	1009.55	93.2417
22	961.341	92.2498	23	918.914	93.1856	24	847.561	91.2779
25	812.849	93.1294	26	762.709	88.0056	27	692.32	89.7891
28	630.609	92.984	29	533.221	94.6829			

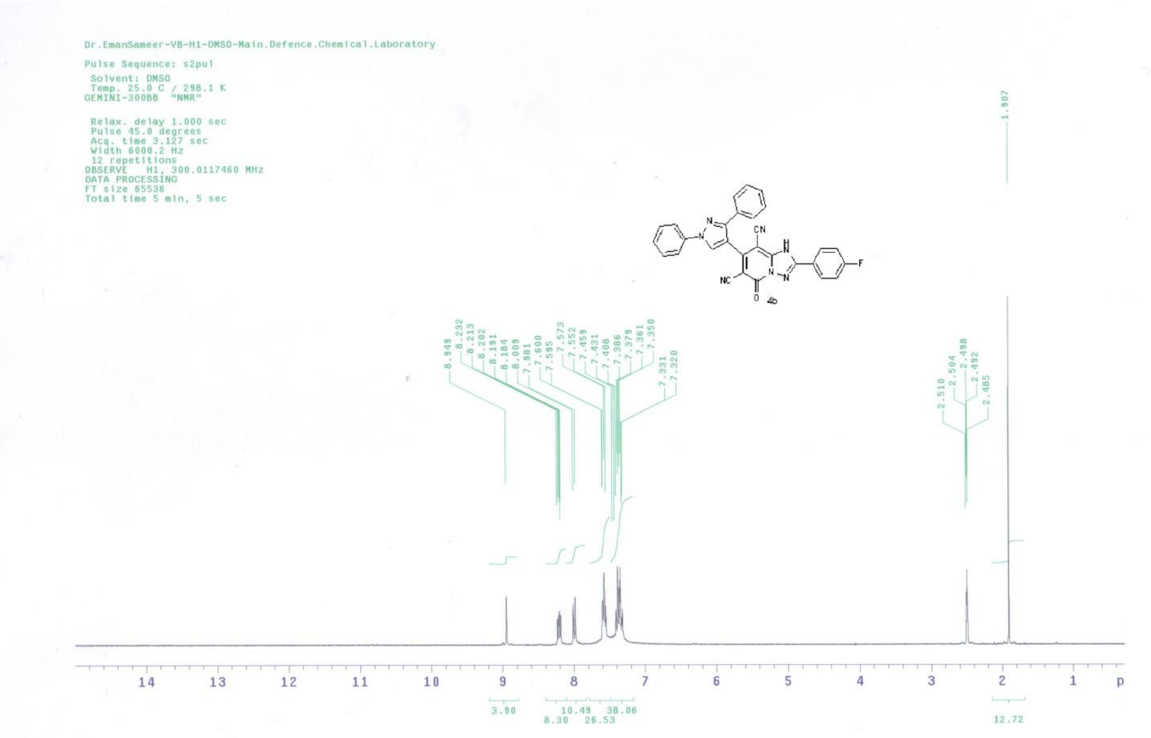
Dr.EmanSameer-VB-H1-DMSO-Main.Defence.Chemical.Laboratory
Pulse Sequence: s2pul
Solvent: DMSO
Temp. 25.0 C / 298.1 K
GEMINI-300BB "NMR"
Relax. delay 1.000 sec
Pulse 45.0 degrees
Acq. time 3.127 sec
Width 6000.2 Hz
12 repetitions
OBSERVE H1, 300.0117460 MHz
DATA PROCESSING
FT size 65536
Total time 5 min, 5 sec
CN
NC
F
O
4b
8.949
8.232
8.213
8.202
8.191
8.184
8.009
7.981
7.600
7.595
7.573
7.552
7.459
7.431
7.408
7.386
7.379
7.361
7.350
7.331
7.320
2.510
2.504
2.498
2.492
2.485
1.907
14 13 12 11 10 9 8 7 6 5 4 3 2 1 p
3.90
10.49
38.06
8.30
26.53
12.72

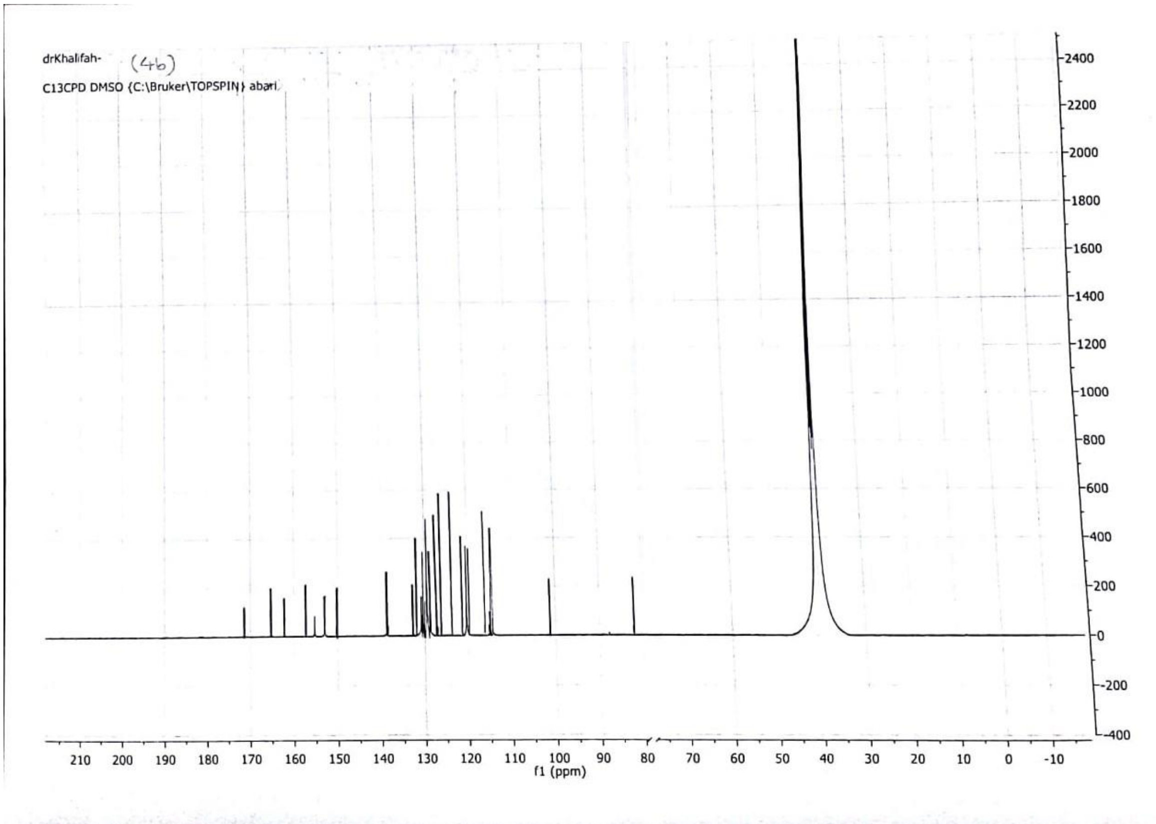
drKhalifah- (4b)
C13CPD DMSO {C:\Bruker\TOPSPIN} abari.
2400
2200
2000
1800
1600
1400
1200
1000
800
600
400
200
0
-200
-400
210 200 190 180 170 160 150 140 130 120 110 100 90 80 70 60 50 40 30 20 10 0 -10
f1 (ppm)

Al-Azhar University
The Regional Center for Mycology & Biotechnology
C:\Xcalibur\data\S\EMAN-SAMIR-V8

2/15/2016 10:59:21 AM
C:\Xcalibur\methods\Mass DIP.meth

RT: 3.65 - 6.00 SM: 15B

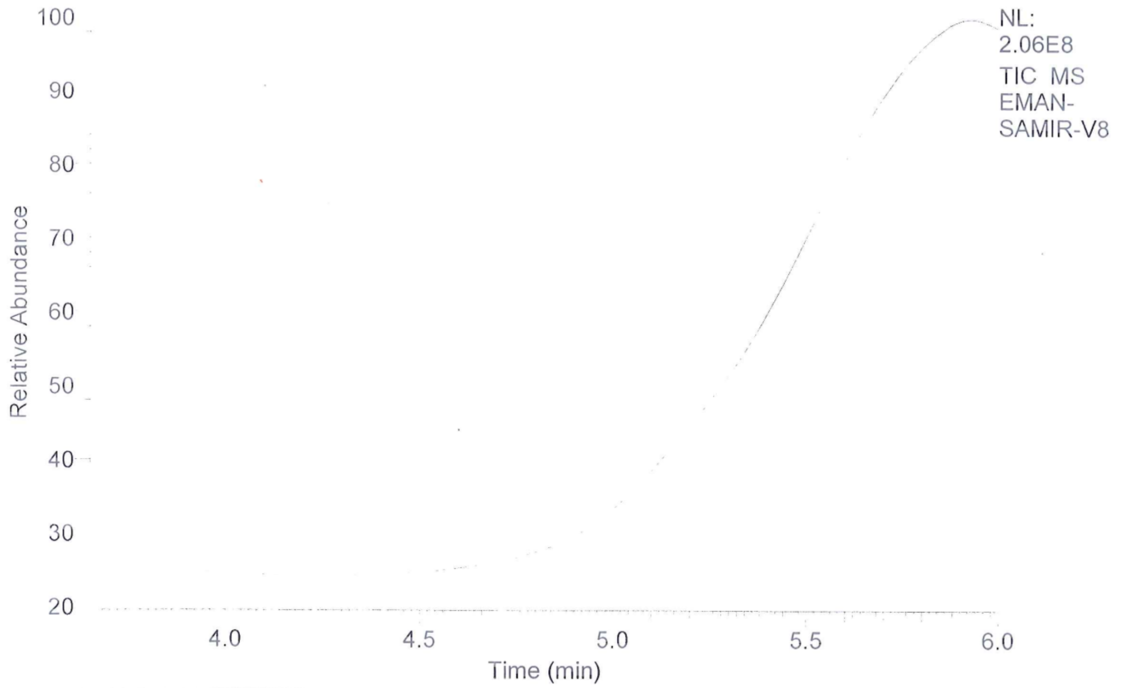

EMAN-SAMIR-V8 #350 RT: 5.87 AV: 1 SB: 2 5.62 , 5.67 NL: 3.02E6
T: {0,0} + c EI Full ms [40.00-1000.00]

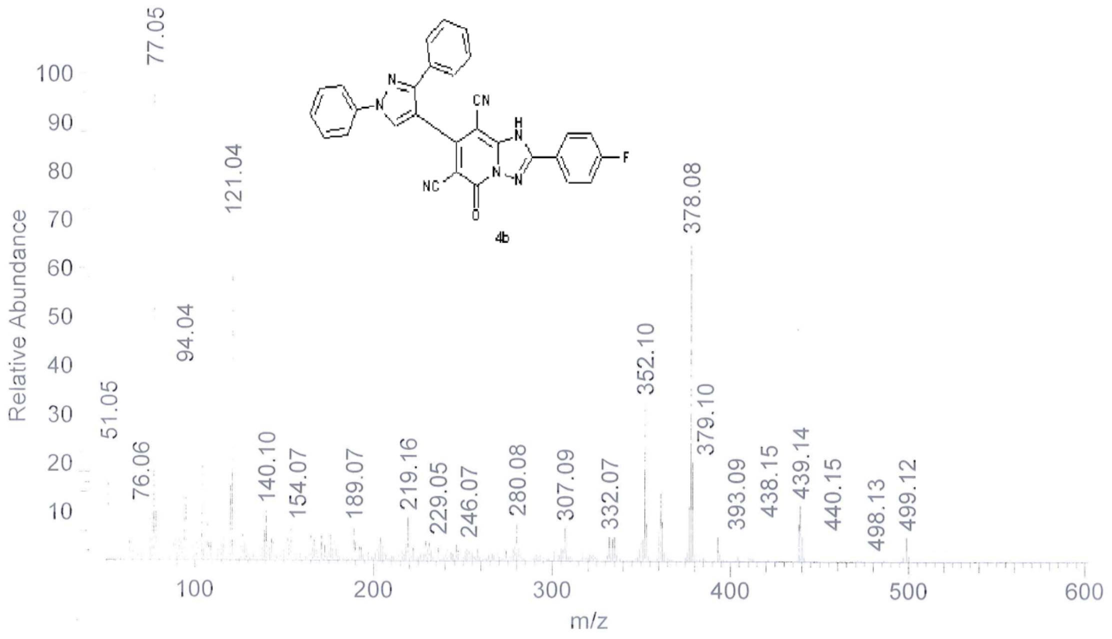

4b

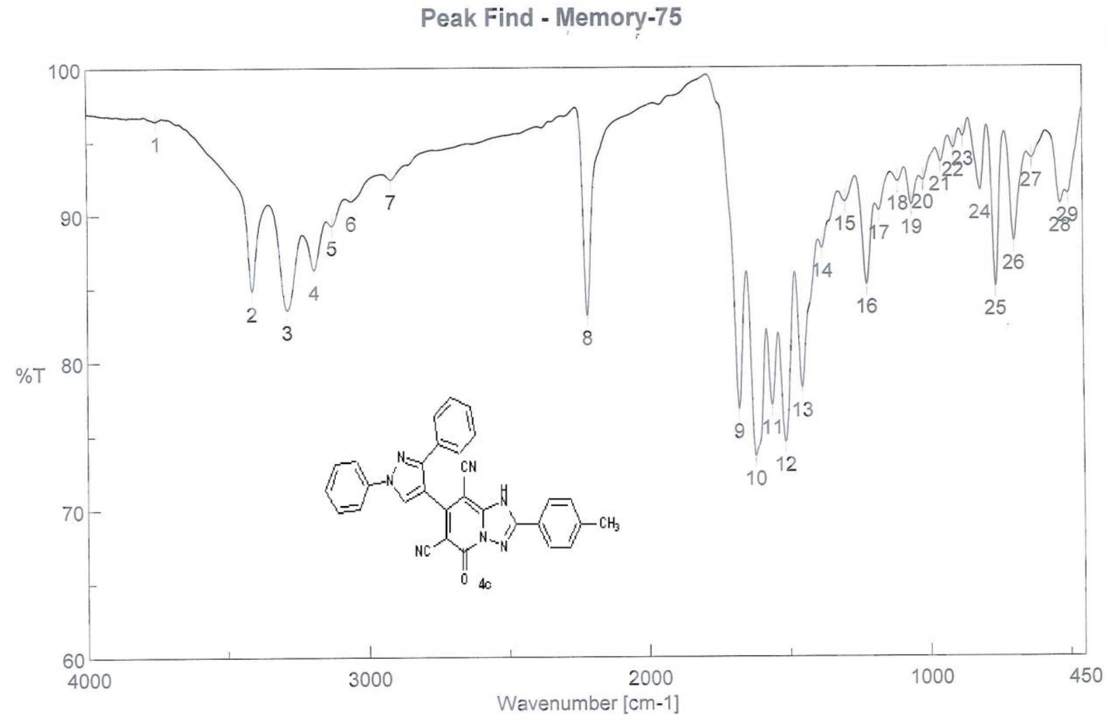

[Comments]

Sample name	V5
Comment	21/4/2016
User	IR
Division	IR
Company	MAC

[Result of Peak Picking]

No.	Position	Intensity	No.	Position	Intensity	No.	Position	Intensity
1	3751.83	96.4407	2	3412.42	84.8152	3	3288.04	83.5565
4	3192.58	86.2656	5	3129.9	89.2713	6	3059.51	90.9281
7	2918.73	92.4031	8	2216.77	83.1174	9	1677.77	76.7889
10	1619.91	73.5929	11	1561.09	77.0482	12	1513.85	74.5451
13	1454.06	78.2182	14	1381.75	87.6242	15	1298.82	90.8159
16	1222.65	85.2068	17	1178.29	90.1832	18	1110.8	92.1918
19	1061.62	90.5723	20	1020.16	92.2723	21	957.484	93.4834
22	911.201	94.463	23	876.488	95.2154	24	815.742	91.5639
25	761.744	85.056	26	696.177	88.1222	27	631.573	93.7541
28	530.328	90.6626	29	503.33	91.3407			

Micro Analytical Center

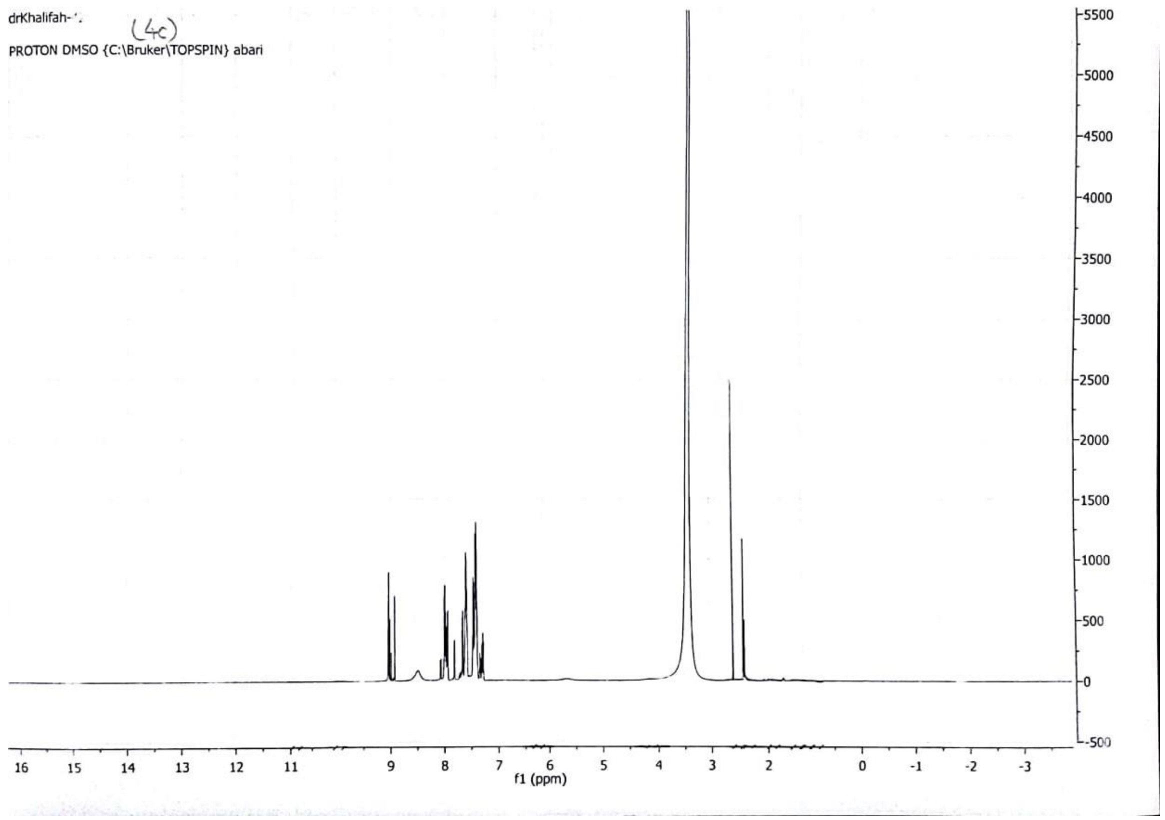
drKhalifah-
(4c)
PROTON DMSO {C:\Bruker\TOPSPIN} abari
f1 (ppm)

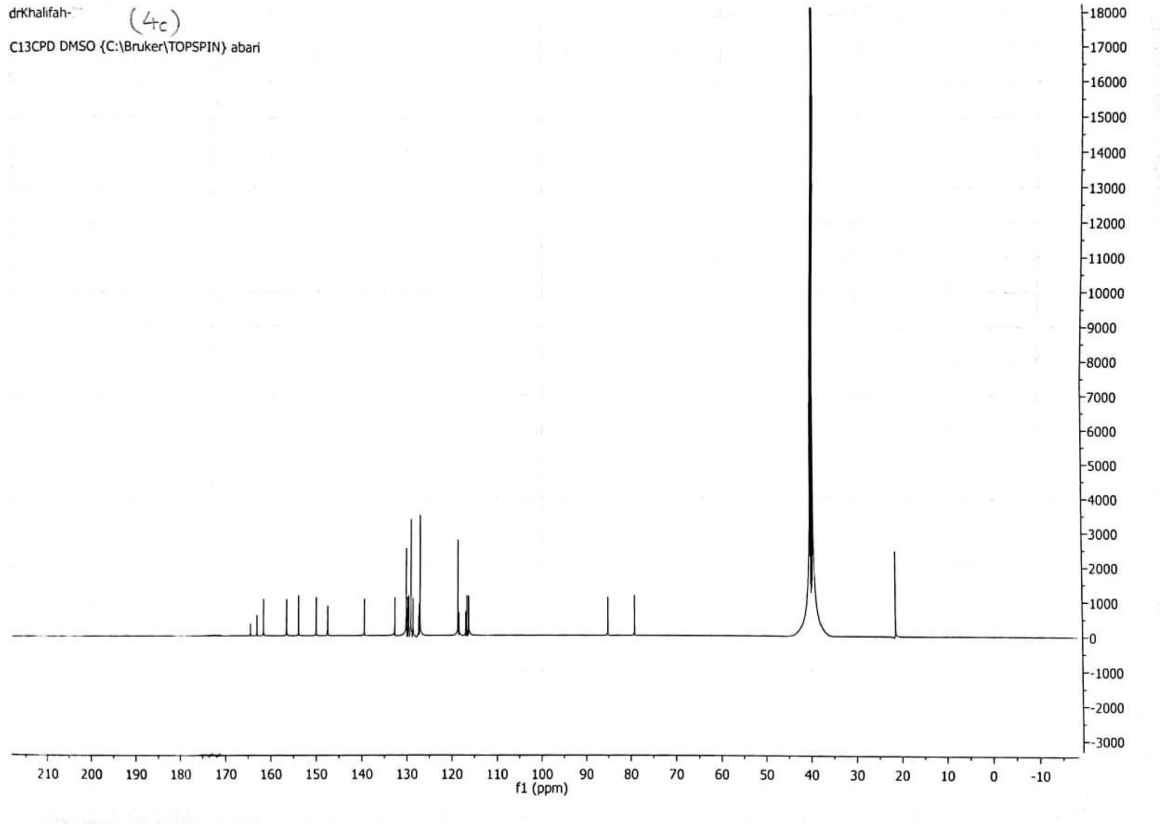
drKhalifah-
(4c)
C13CPD DMSO {C:\Bruker\TOPSPIN} abari
f1 (ppm)

Al-Azhar University
The Regional Center for Mycology & Biotechnology
C:\Xcalibur\data\S\EMAN-SAMIR-V5

2/16/2016 10:44:21 AM
C:\Xcalibur\methods\Mass DIP.meth

RT: 0.00 - 6.00 SM: 15B

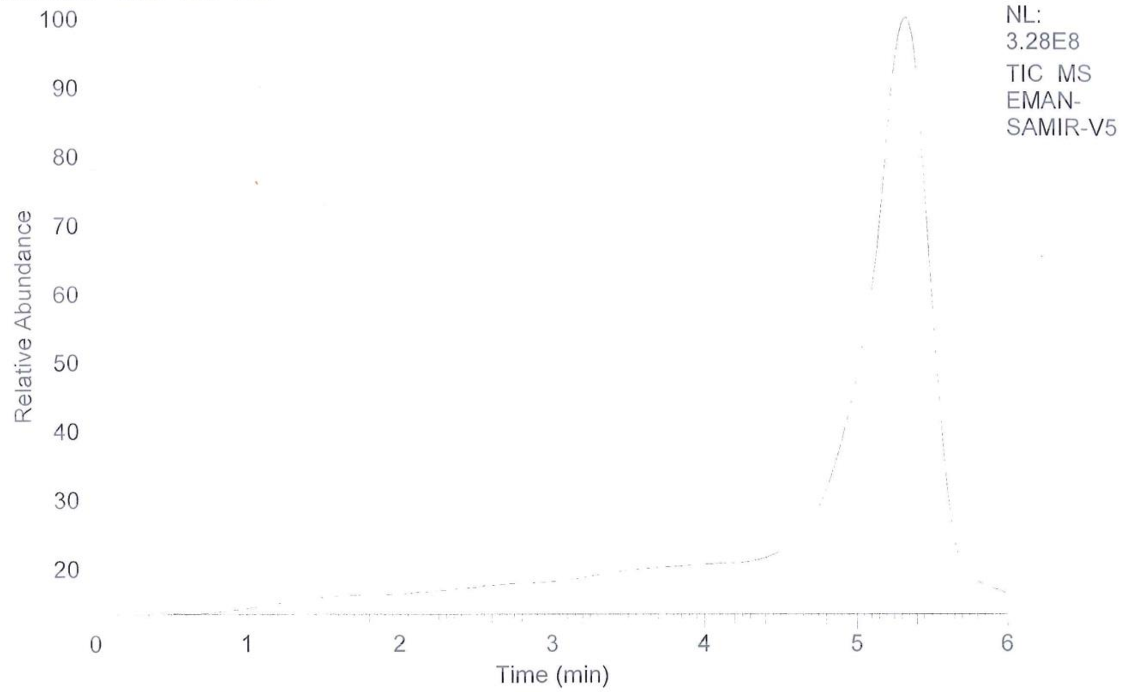

EMAN-SAMIR-V5 #337 RT: 5.66 AV: 1 SB: 2 5.72 , 5.71 NL: 1.12E6
T: {0,0} + c EI Full ms [40.00-1000.00]

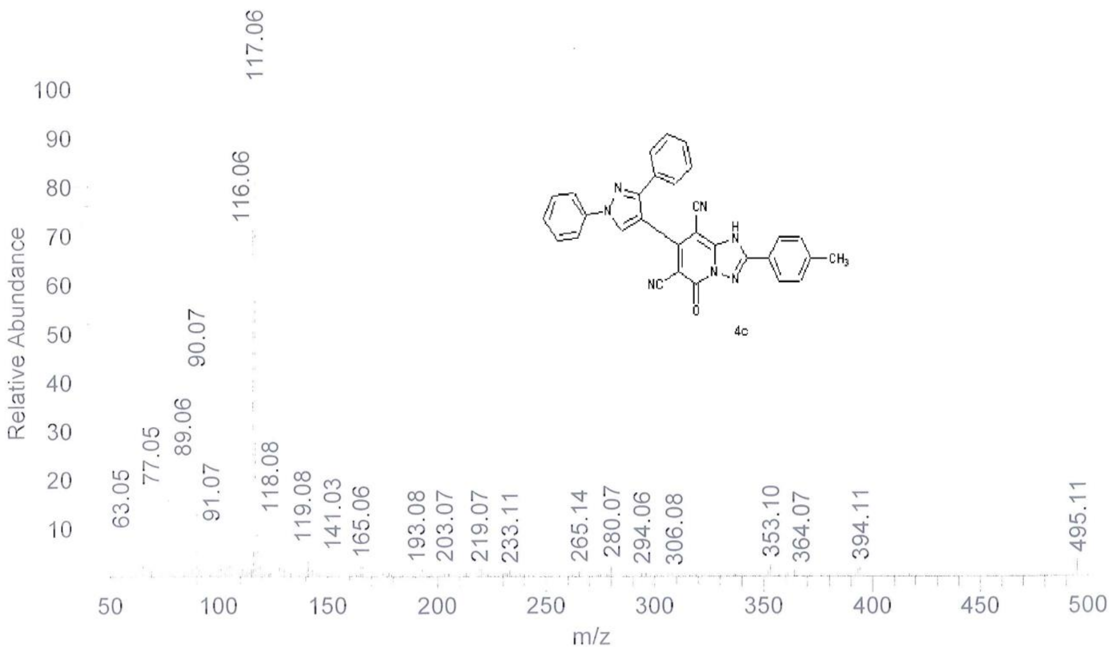

Peak Find - Memory-166

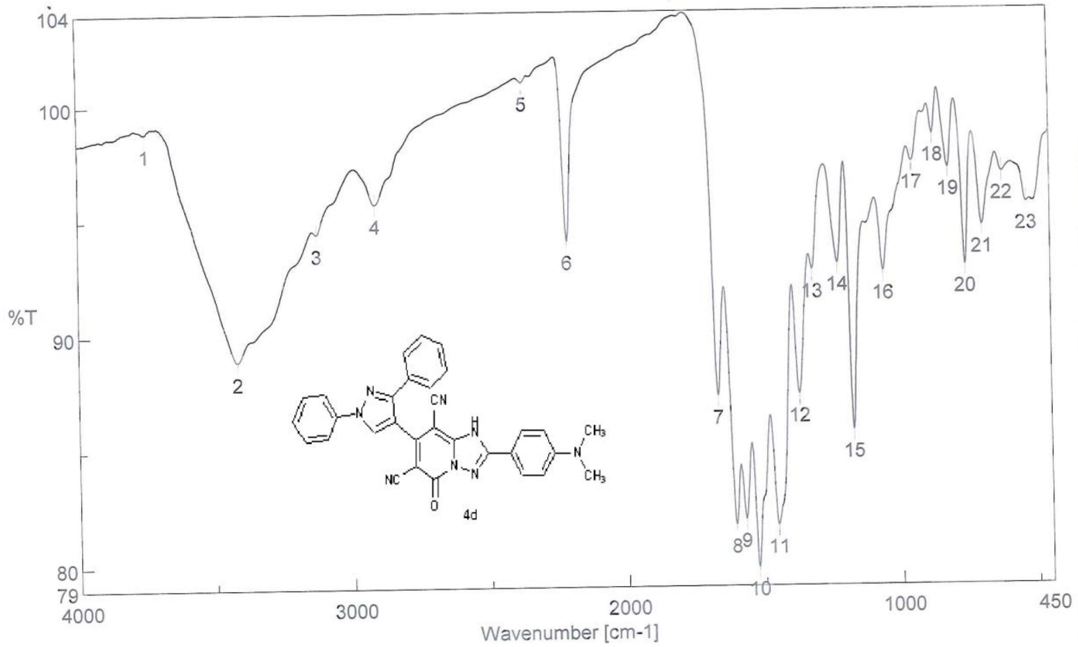

[Comments]

Sample name	V3
Comment	21/4/2016
User	IR
Division	IR
Company	MAC

[Result of Peak Picking]

No.	Position	Intensity	No.	Position	Intensity	No.	Position	Intensity
1	3752.8	98.8256	2	3420.14	88.889	3	3129.9	94.3897
4	2914.88	95.6619	5	2372.01	100.941	6	2212.92	93.9934
7	1668.12	87.2539	8	1607.38	81.6467	9	1571.7	81.8936
10	1527.35	79.7889	11	1455.03	81.6197	12	1372.1	87.3005
13	1321.96	92.6402	14	1229.4	92.937	15	1174.44	85.7382
16	1061.62	92.557	17	953.627	97.3232	18	875.524	98.5203
19	819.598	97.0329	20	759.816	92.8178	21	697.141	94.5193
22	622.895	96.8236	23	535.15	95.5007			

Micro Analytical Center
جامعة القاهرة
مركز التحاليل

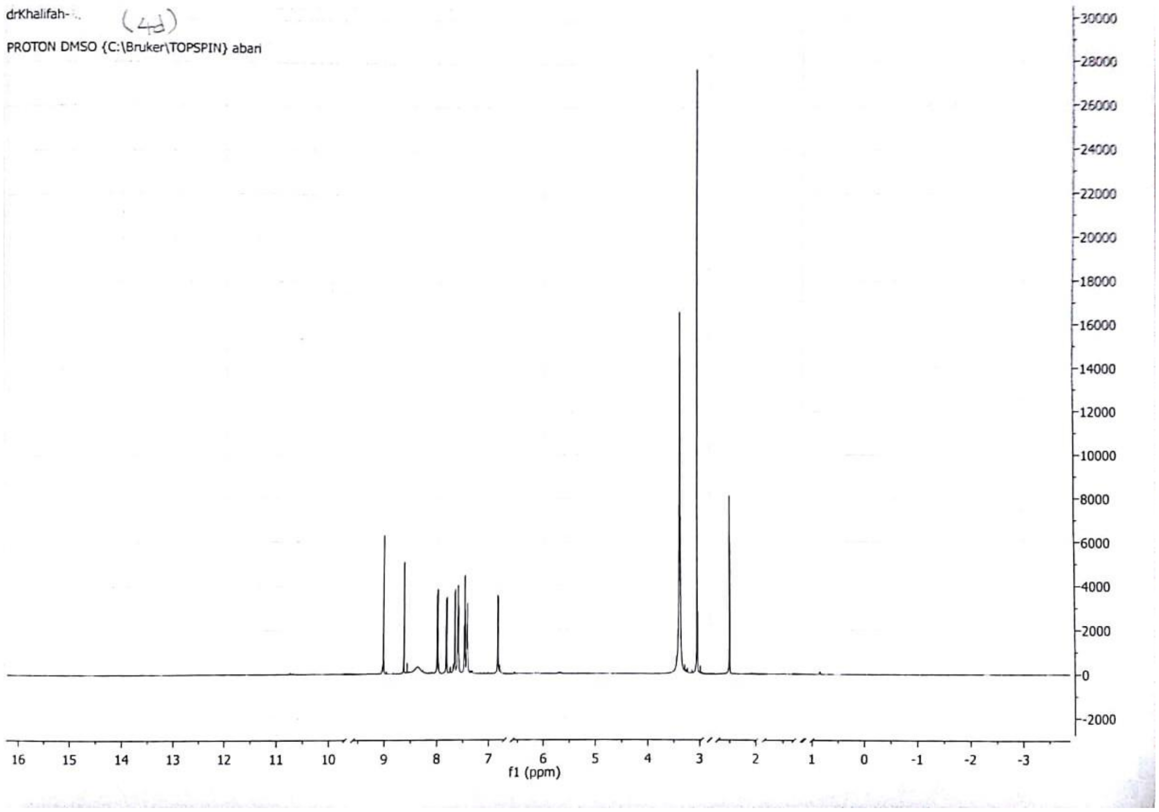
drKhalifah-
(4d)
PROTON DMSO {C:\Bruker\TOPSPIN} abari
f1 (ppm)

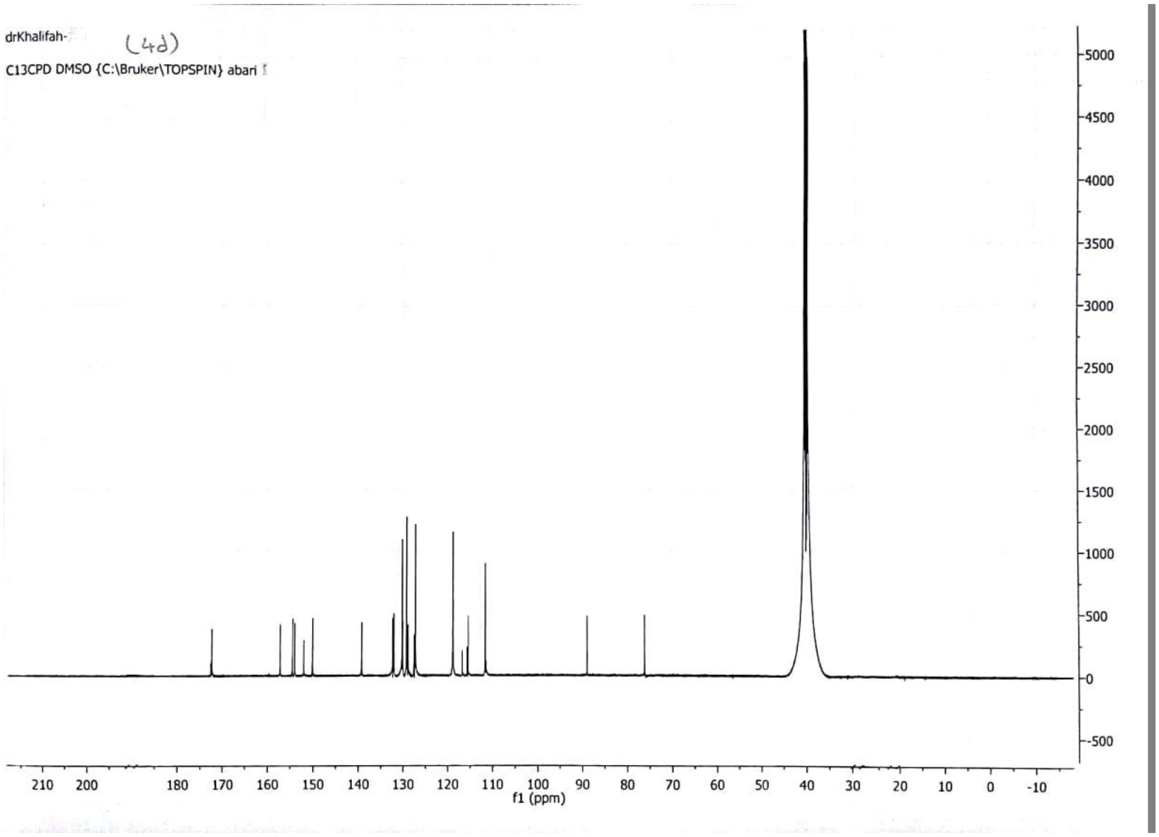
drKhalifah-
(4d)
C13CPD DMSO {C:\Bruker\TOPSPIN} abari
f1 (ppm)

Al-Azhar University
The Regional Center for Mycology & Biotechnology
C:\Xcalibur\data\S\EMAN-SAMIR-V3

2/16/2016 9:55:49 AM
C:\Xcalibur\methods\Mass DIP.meth

RT: 0.00 - 6.00 SM: 15B

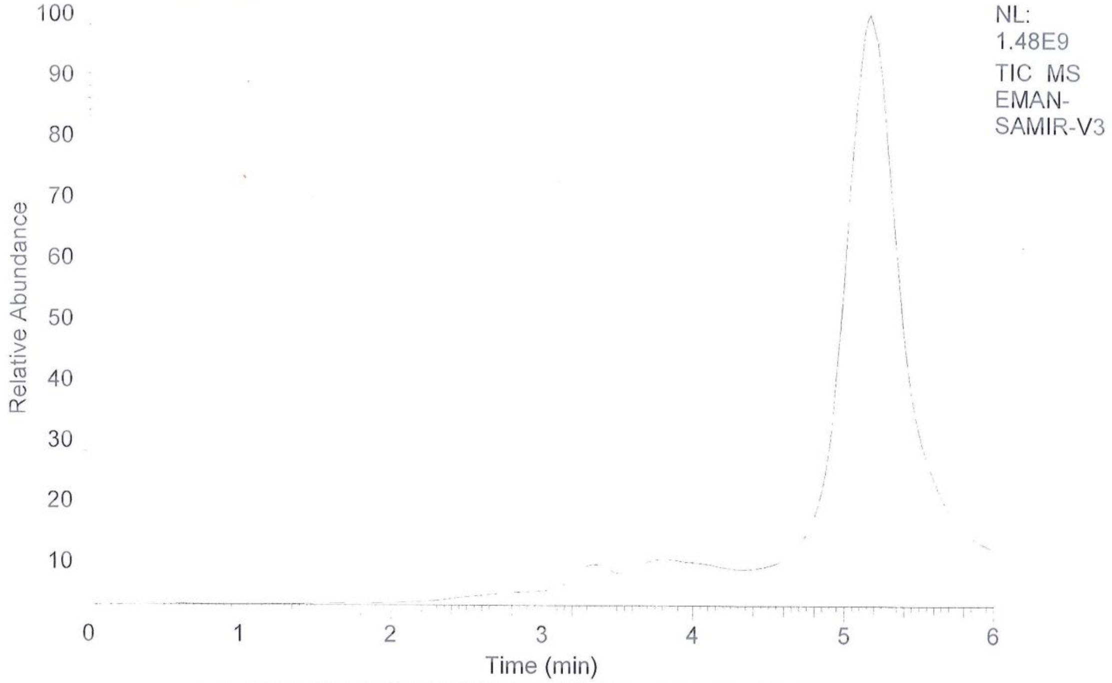

EMAN-SAMIR-V3 #342 RT: 5.74 AV: 1 SB: 2 5.74 , 5.69 NL: 1.17E5
T: {0,0} + c El Full ms [40.00-1000.00]

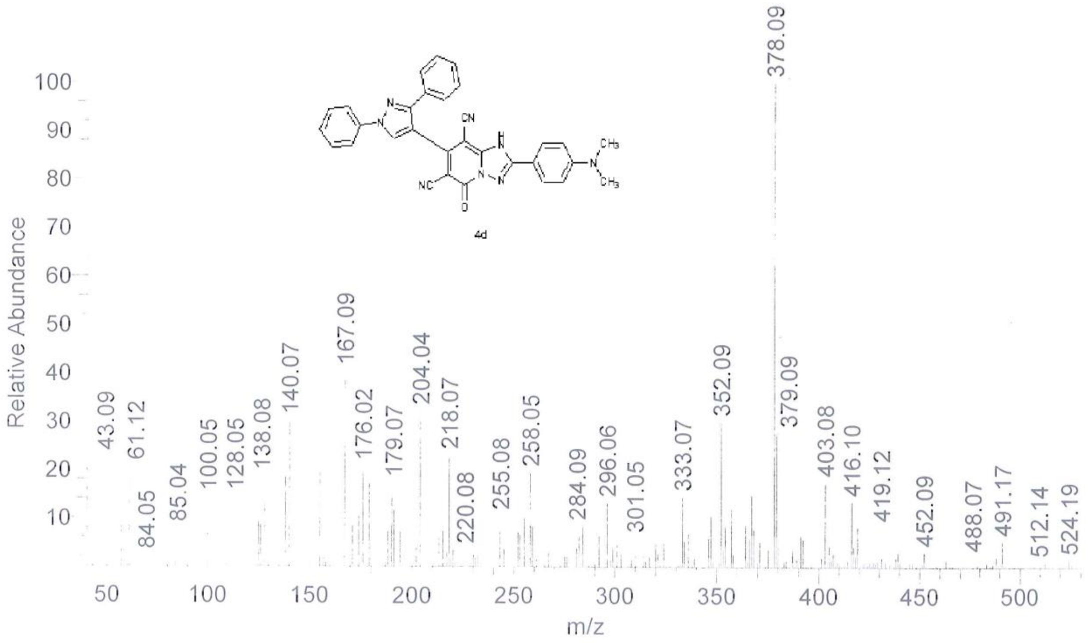

Peak Find - Memory-83

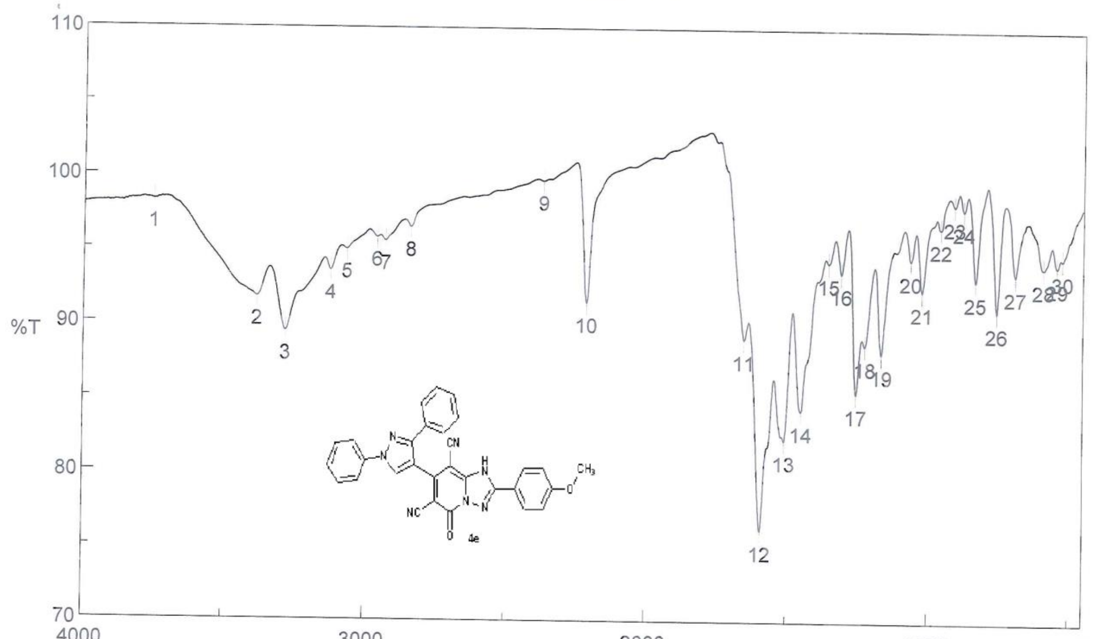

[Comments]

Sample name	V2
Comment	21/4/2016
User	IR
Division	IR
Company	MAC

[Result of Peak Picking]

No.	Position	Intensity	No.	Position	Intensity	No.	Position	Intensity
1	3752.8	98.2425	2	3383.5	91.7303	3	3283.21	89.4176
4	3124.12	93.5176	5	3067.23	94.9572	6	2959.23	95.7752
7	2929.34	95.5476	8	2839.67	96.4601	9	2368.16	99.6422
10	2212.92	91.4222	11	1652.7	89.0038	12	1592.91	76.1516
13	1509.99	82.2487	14	1450.21	84.2486	15	1355.71	94.192
16	1310.39	93.5207	17	1256.4	85.4358	18	1225.54	88.6356
19	1166.72	88.1039	20	1064.51	94.4234	21	1024.02	92.3392
22	957.484	96.5836	23	909.272	98.1144	24	876.488	97.8065
25	833.098	93.0488	26	756.923	90.963	27	691.355	93.4501
28	592.039	93.9612	29	543.828	94.047	30	524.543	94.551

Micro Analytical Center
جامعة القاهرة

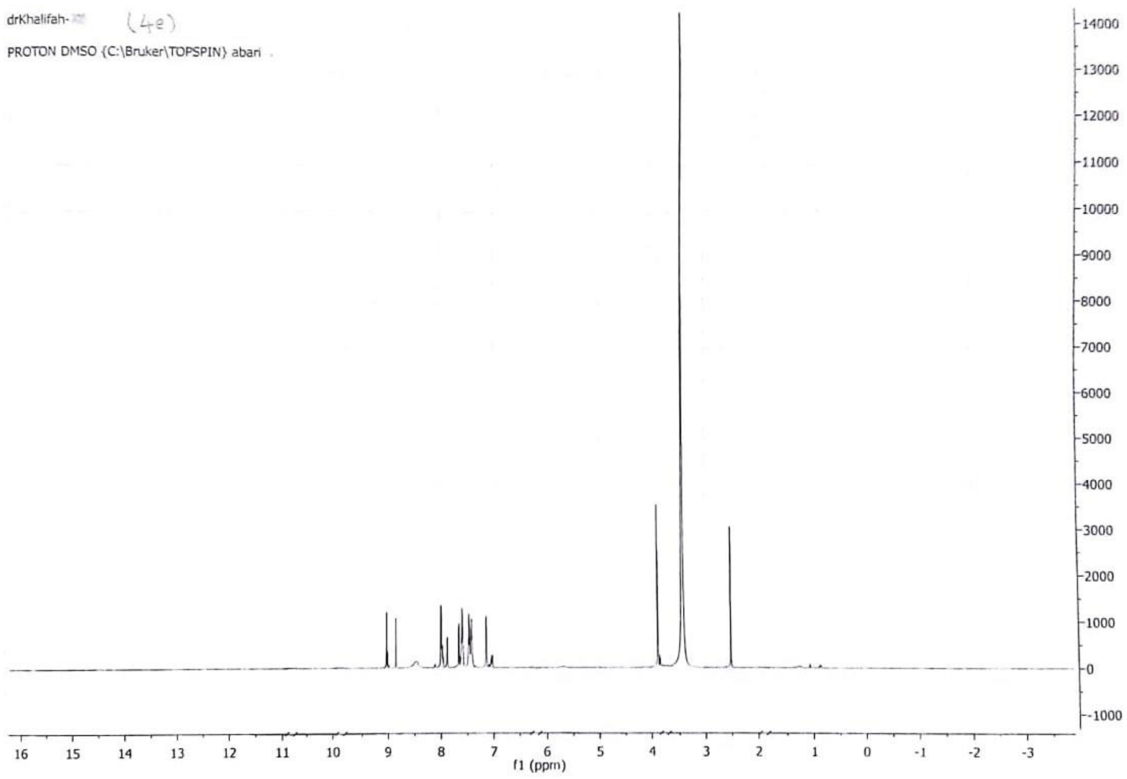
drKhalifah-
(4e)
PROTON DMSO {C:\Bruker\TOPSPIN} abari
f1 (ppm)

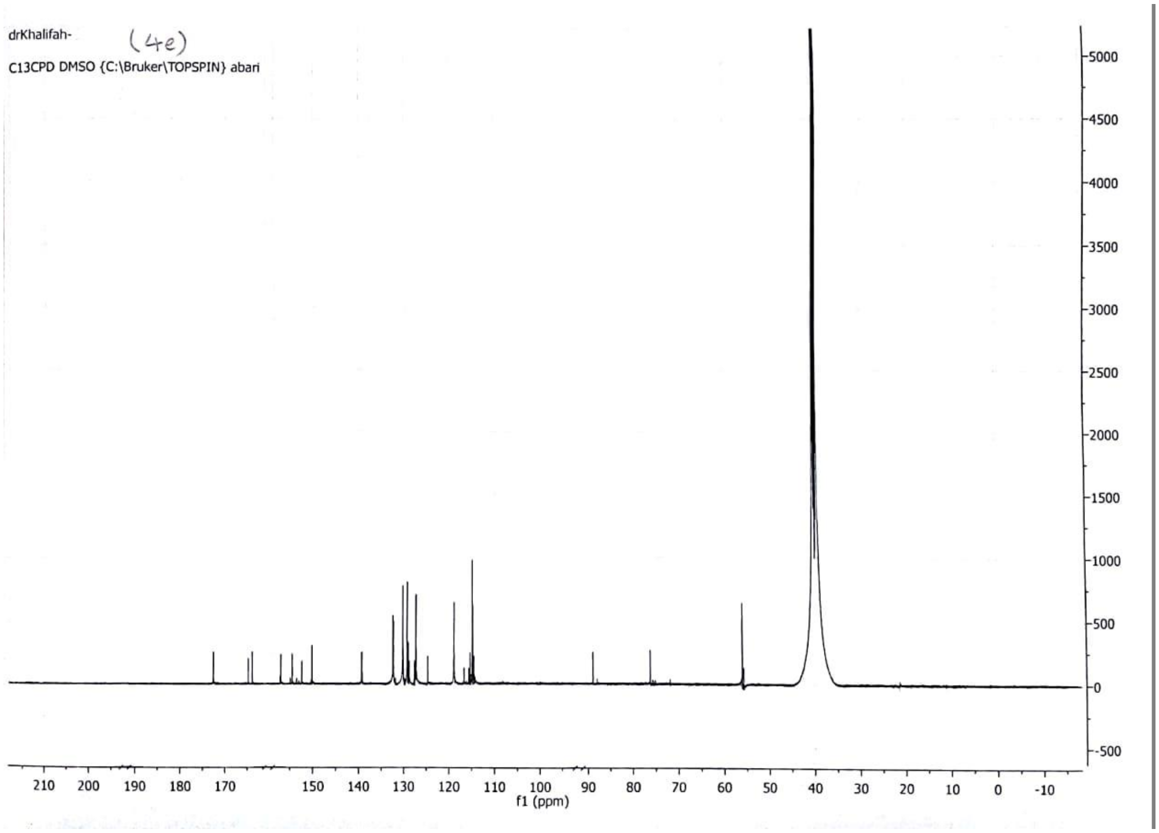
drKhalifah-
(4e)
C13CPD DMSO {C:\Bruker\TOPSPIN} abari
f1 (ppm)

Al-Azhar University
The Regional Center for Mycology & Biotechnology
C:\Xcalibur\data\S\EMAN-SAMIR-V2

2/16/2016 9:27:34 AM
C:\Xcalibur\methods\Mass DIP.meth

RT: 0.35 - 4.57 SM: 15B

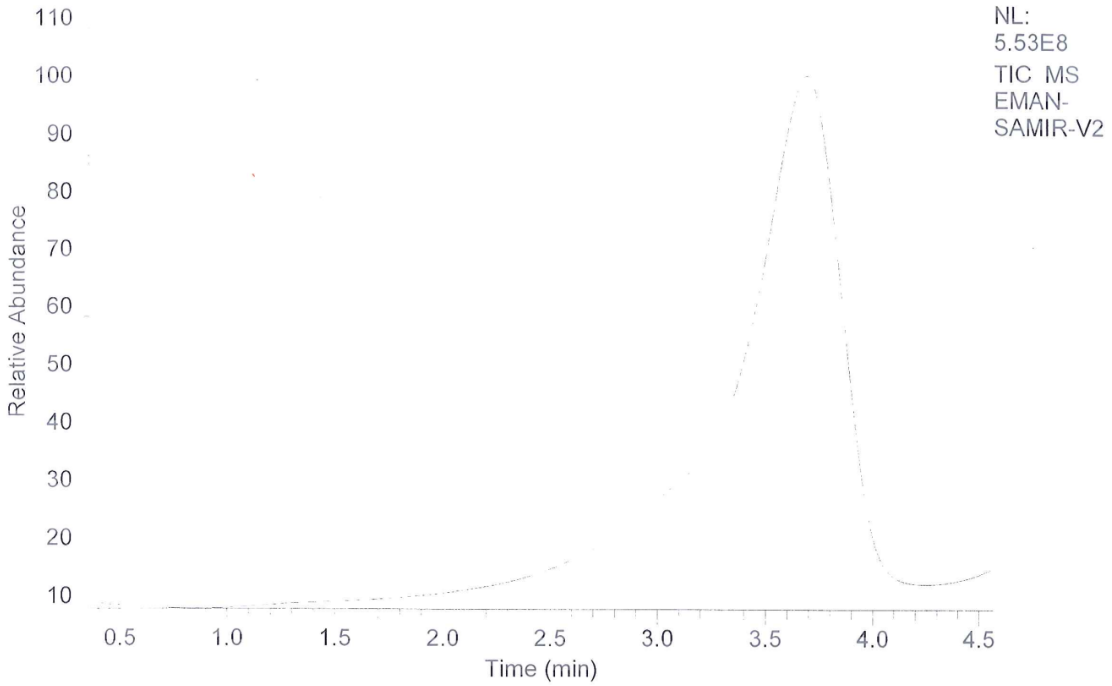

EMAN-SAMIR-V2 #349 RT: 5.86 AV: 1 SB: 2 5.87 , 5.89 NL: 4.85E5
T: {0,0} + c EI Full ms [40.00-1000.00]

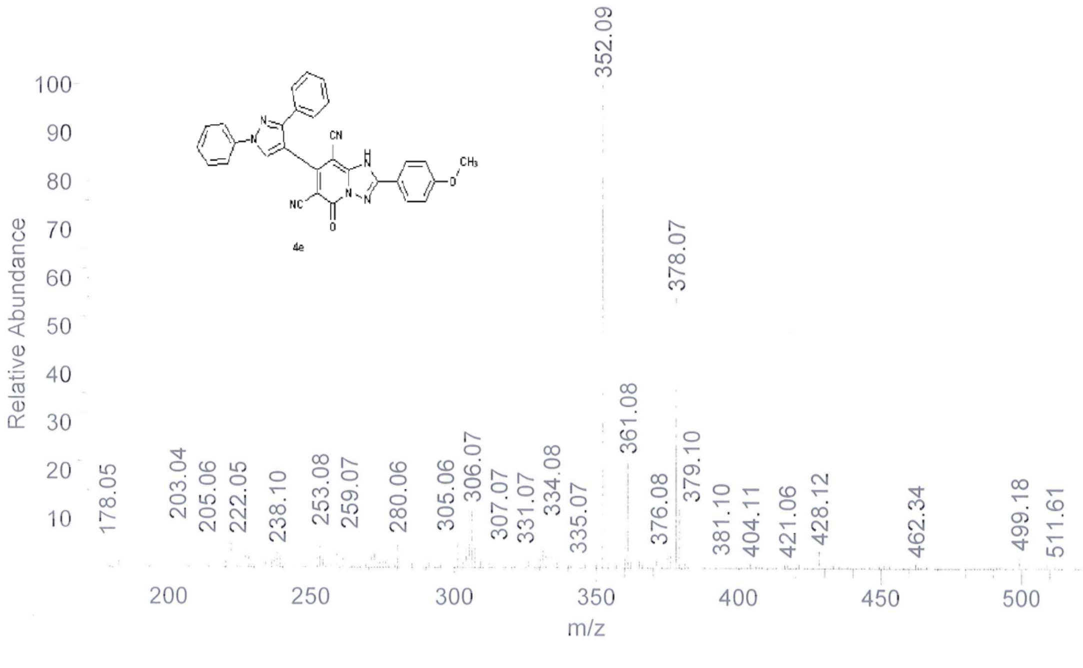

Peak Find - Memory-146

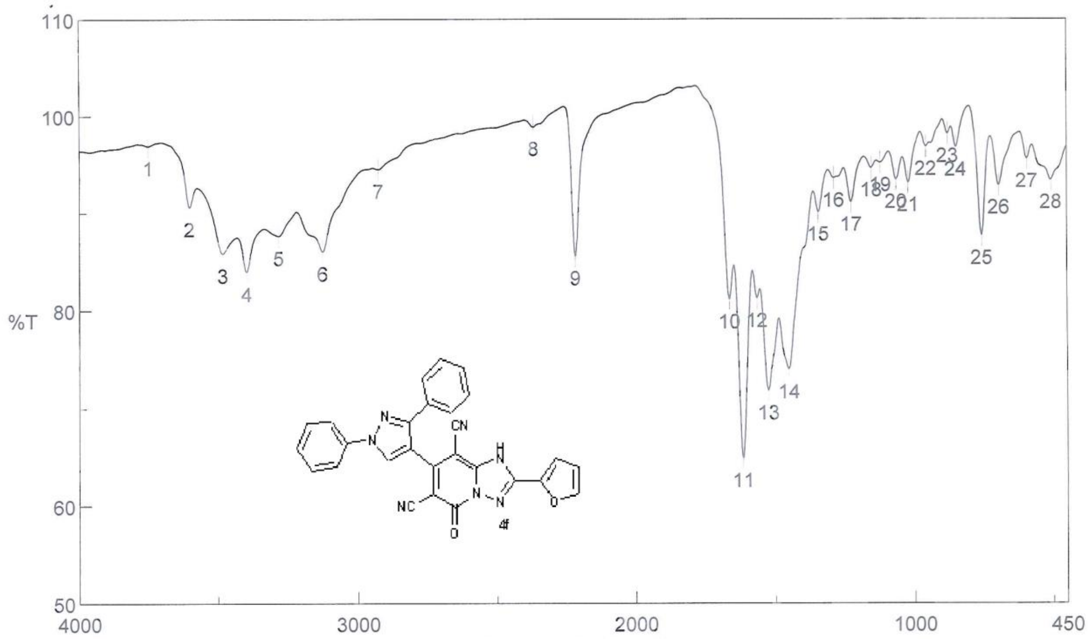

[Comments]
Sample name V7
Comment 21/4/2016
User IR
Division IR
Company MAC

[Result of Peak Picking]

No.	Position	Intensity	No.	Position	Intensity	No.	Position	Intensity
1	3751.83	96.8801	2	3602.38	90.5856	3	3483.78	85.8297
4	3398.92	83.9347	5	3284.18	87.6042	6	3126.04	86.0249
7	2926.45	94.4632	8	2366.23	98.8299	9	2215.81	85.5495
10	1663.3	81.1049	11	1616.06	64.883	12	1564.95	81.2385
13	1524.45	71.8292	14	1451.17	73.9846	15	1344.14	90.0559
16	1289.18	93.5517	17	1226.5	91.0857	18	1154.19	94.603
19	1121.4	95.1339	20	1064.51	93.3897	21	1020.16	93.0888
22	956.52	96.7845	23	878.417	98.1178	24	848.525	96.7064
25	755.959	87.6408	26	694.248	92.8085	27	593.004	95.5088
28	507.187	93.3419						

Analytical Center
جامعة القاهرة
مركز التحاليل الدقيقة

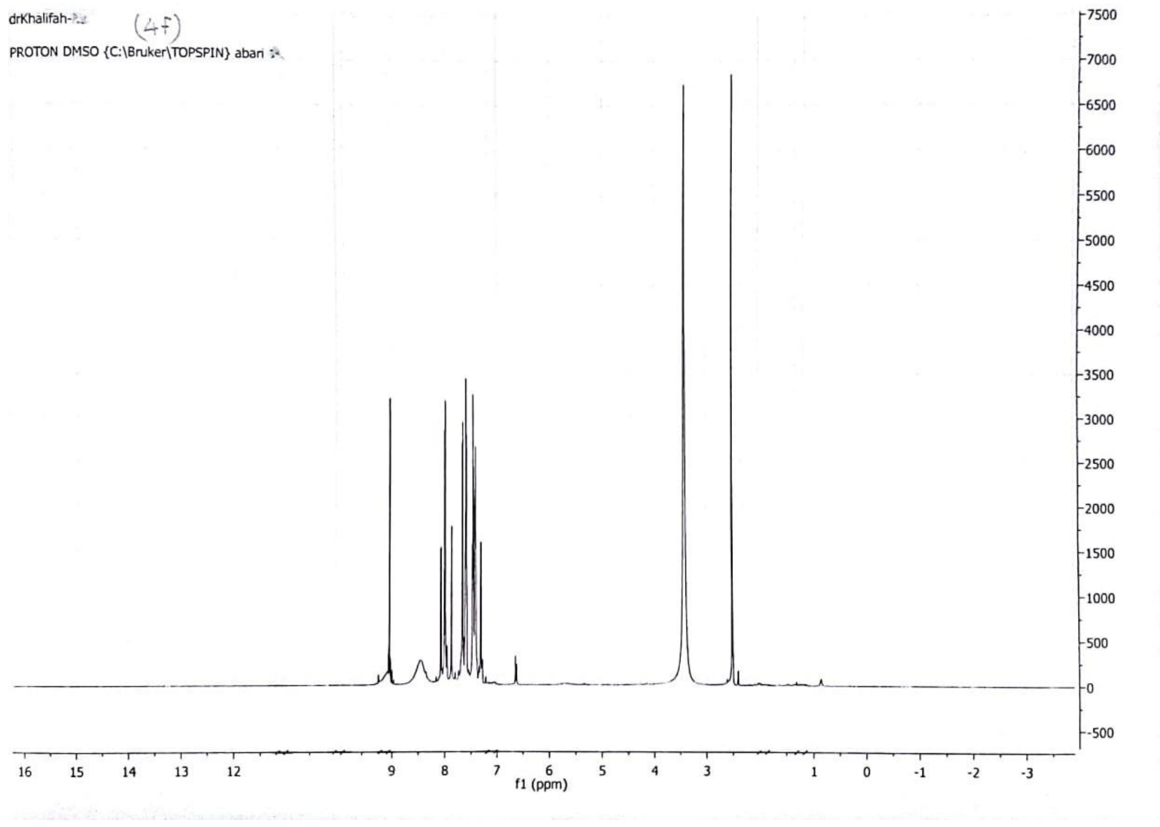
drKhalifah-
(4f)
PROTON DMSO {C:\Bruker\TOPSPIN} abari
f1 (ppm)

Peak Find - Memory-132

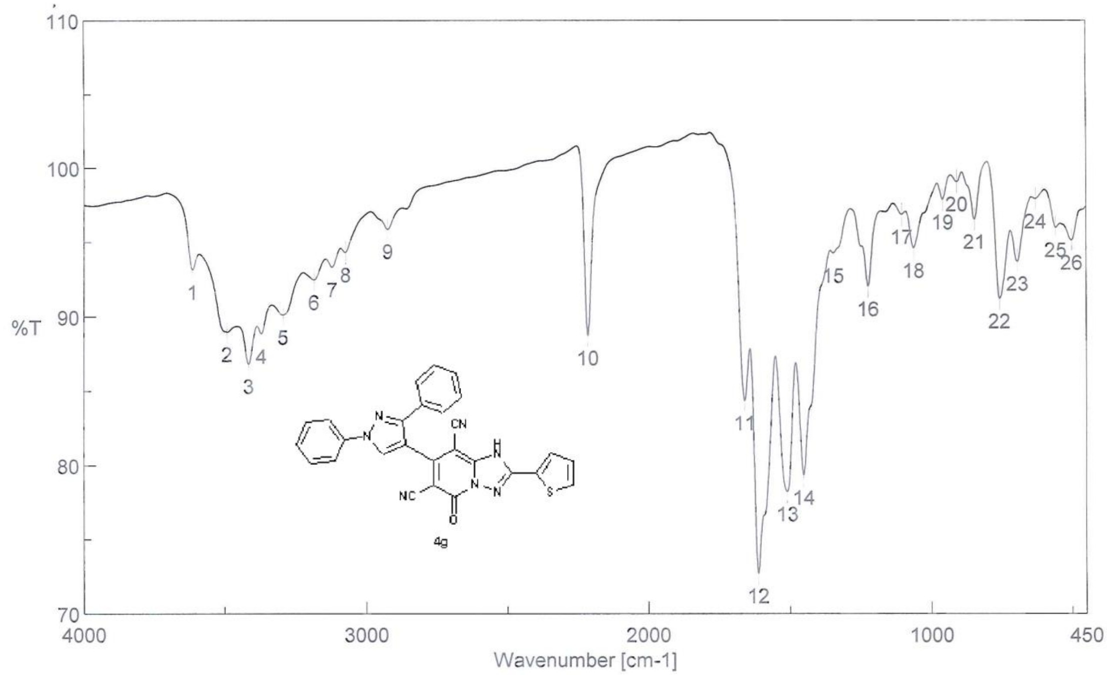

[Comments]

Sample name	V9
Comment	21/4/2016
User	IR
Division	IR
Company	MAC

[Result of Peak Picking]

No.	Position	Intensity	No.	Position	Intensity	No.	Position	Intensity
1	3614.91	93.1463	2	3492.45	88.9847	3	3416.28	86.823
4	3371.92	88.8497	5	3294.79	90.1284	6	3185.83	92.468
7	3122.19	93.3126	8	3074.94	94.3055	9	2924.52	95.8622
10	2213.88	88.725	11	1659.45	84.3218	12	1613.16	72.7009
13	1509.99	78.2114	14	1452.14	79.318	15	1347.03	94.2343
16	1223.61	92.0072	17	1105.01	96.8431	18	1062.59	94.5773
19	959.412	97.8438	20	908.308	99.05	21	845.633	96.5255
22	756.923	91.2037	23	693.284	93.6961	24	629.644	97.908
25	557.327	95.972	26	501.401	95.119			

Analytical Center
التحاليل

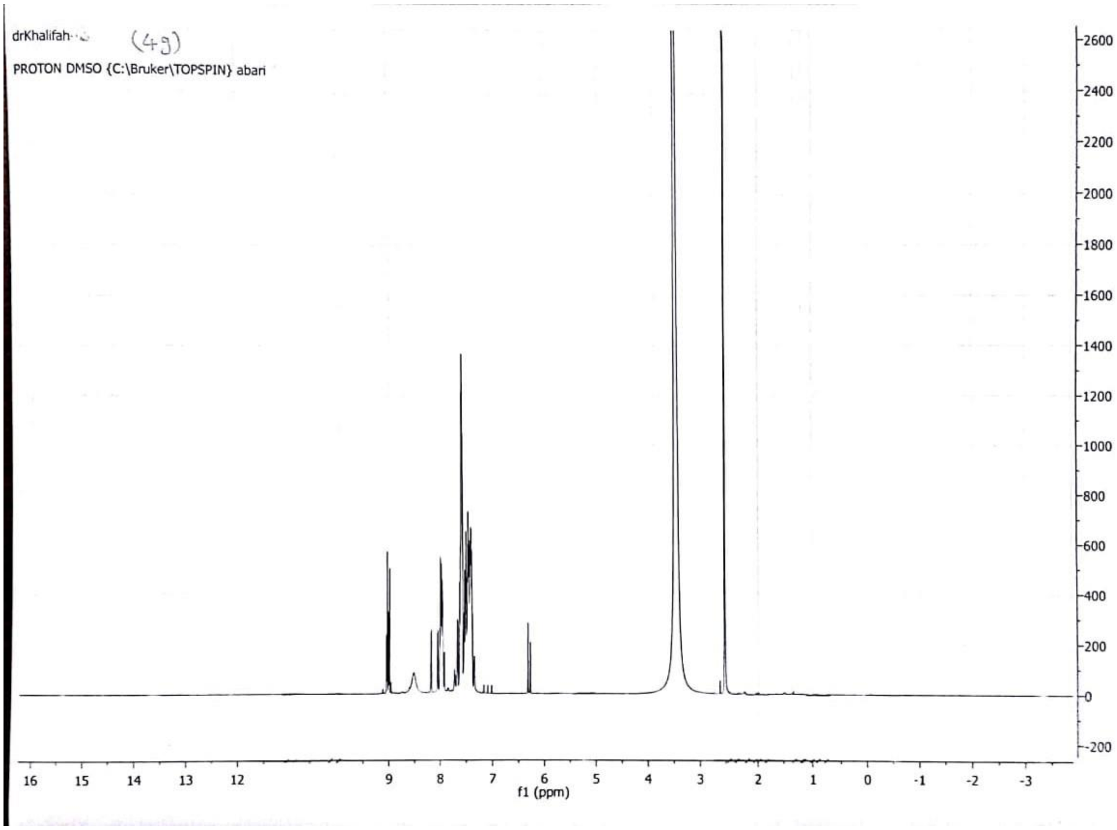
drKhalifah-
(4g)
PROTON DMSO {C:\Bruker\TOPSPIN} abari
f1 (ppm)

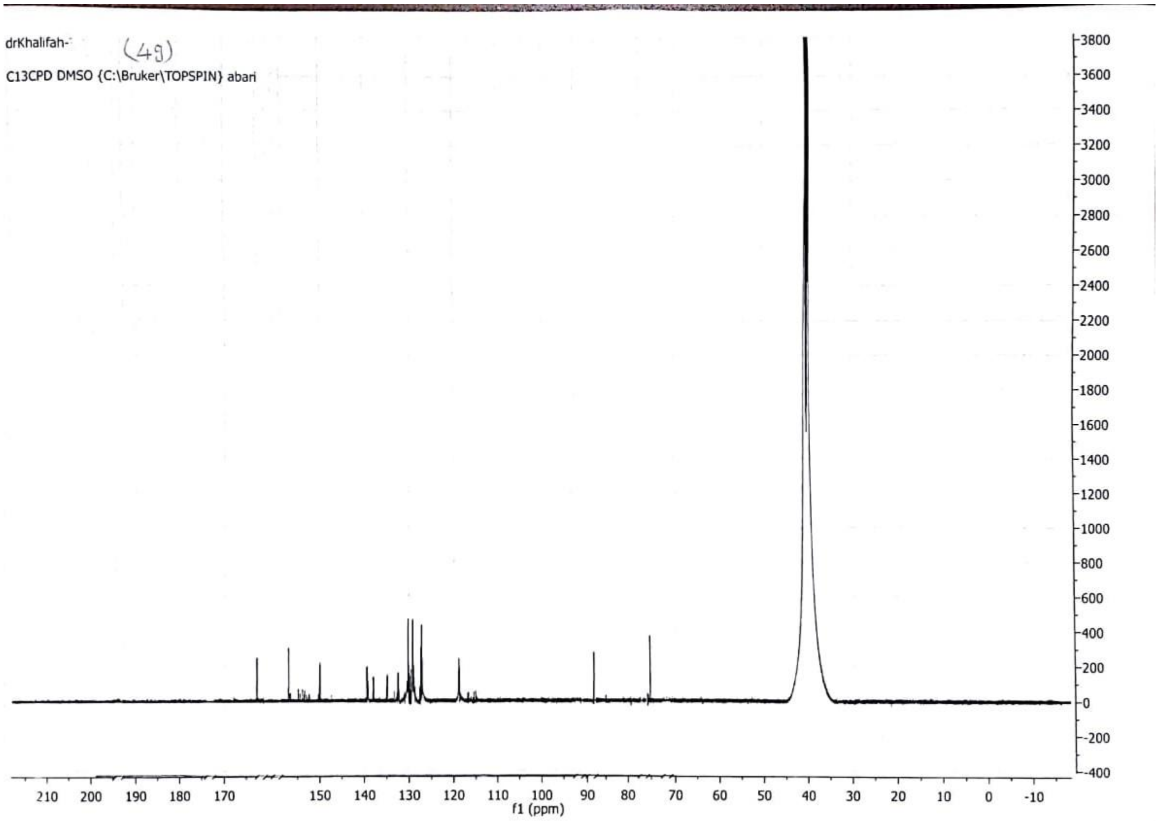
drKhalifah-
(4g)
C13CPD DMSO {C:\Bruker\TOPSPIN} abari
f1 (ppm)

Al-Azhar University
The Regional Center for Mycology & Biotechnology
C:\Xcalibur\data\S\EMAN-SAMIR-V9

2/15/2016 12:18:37 PM
C:\Xcalibur\methods\Mass DIP.meth

RT: 0.00 - 6.00 SM: 15B

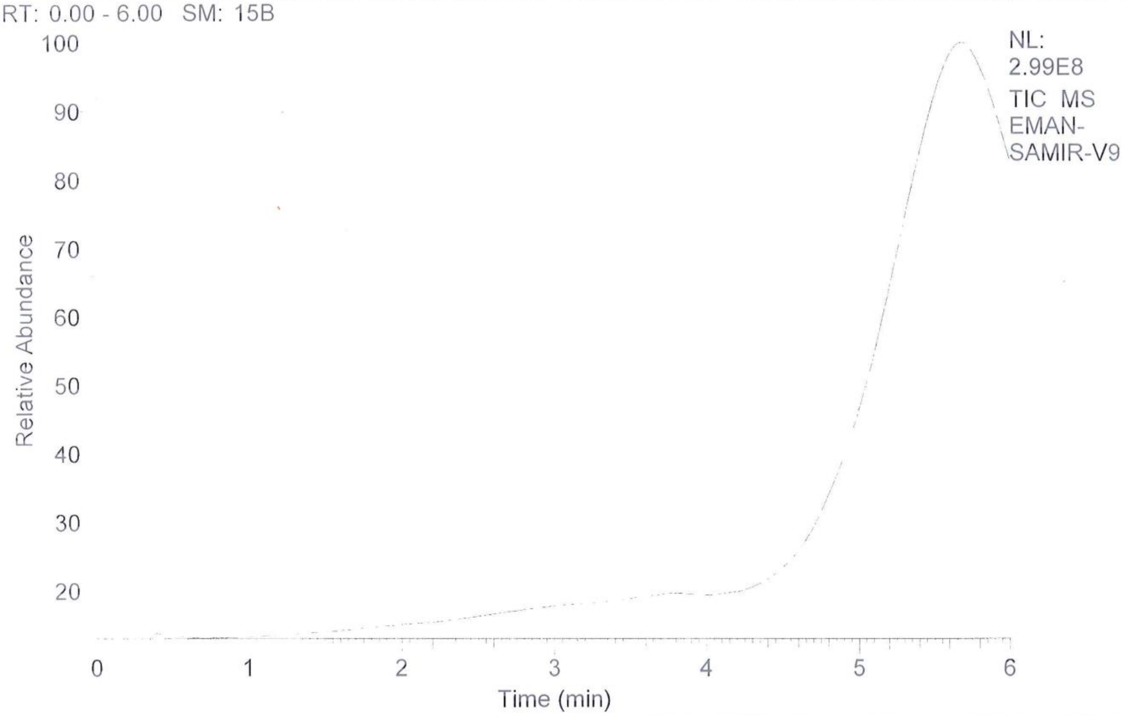

EMAN-SAMIR-V9 #364 RT: 6.11 AV: 1 NL: 2.17E7
T: {0,0} + c EI Full ms [40.00-1000.00]

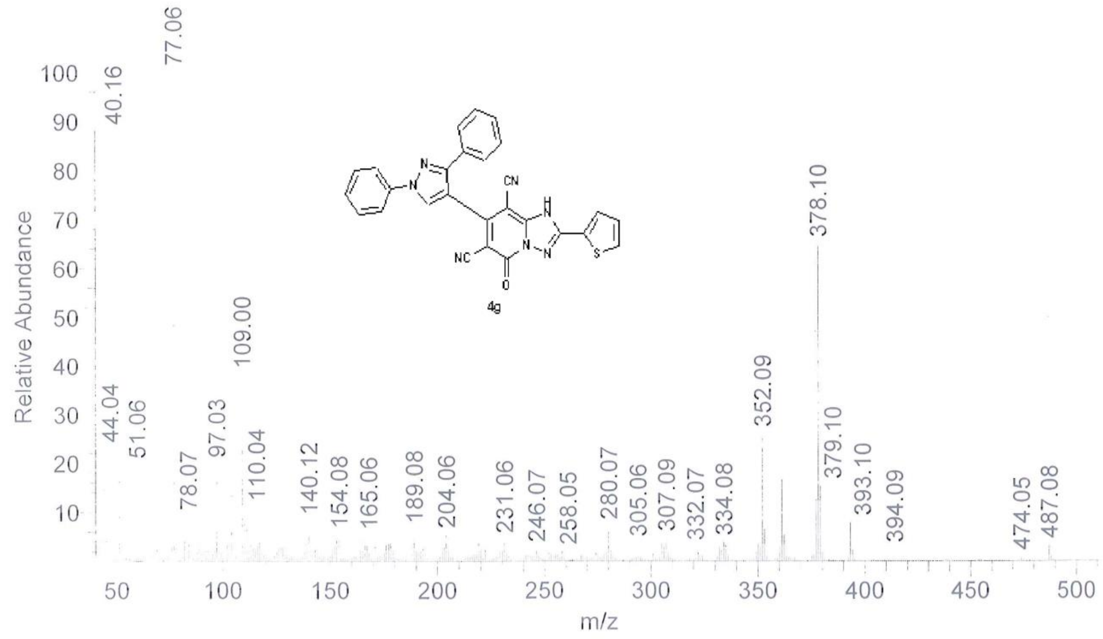

Peak Find - Memory-105

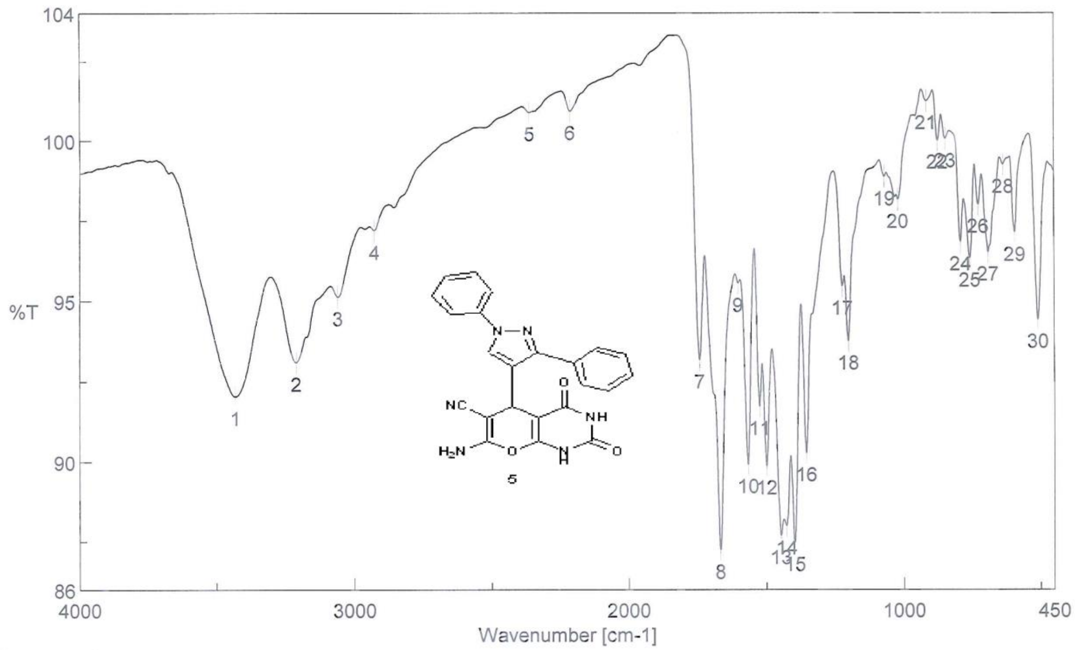

[Comments]

Sample name	G12
Comment	21/4/2016
User	IR
Division	IR
Company	MAC

[Result of Peak Picking]

No.	Position	Intensity	No.	Position	Intensity	No.	Position	Intensity
1	3432.67	92.0263	2	3211.86	93.0937	3	3060.48	95.1261
4	2927.41	97.1889	5	2360.44	100.883	6	2210.99	100.919
7	1741.41	93.1718	8	1667.16	87.2312	9	1602.56	95.5524
10	1566.88	89.9075	11	1524.45	91.7129	12	1499.38	89.8574
13	1448.28	87.6804	14	1428.03	87.9813	15	1398.14	87.464
16	1355.71	90.2735	17	1223.61	95.4643	18	1201.43	93.7594
19	1069.33	98.8579	20	1019.19	98.1515	21	915.058	101.241
22	873.596	99.9985	23	845.633	100.044	24	790.671	96.8366
25	756.923	96.3315	26	726.068	97.9993	27	690.391	96.5187
28	635.43	99.2373	29	593.004	97.1263	30	509.115	94.4356

Micro Analytical Center

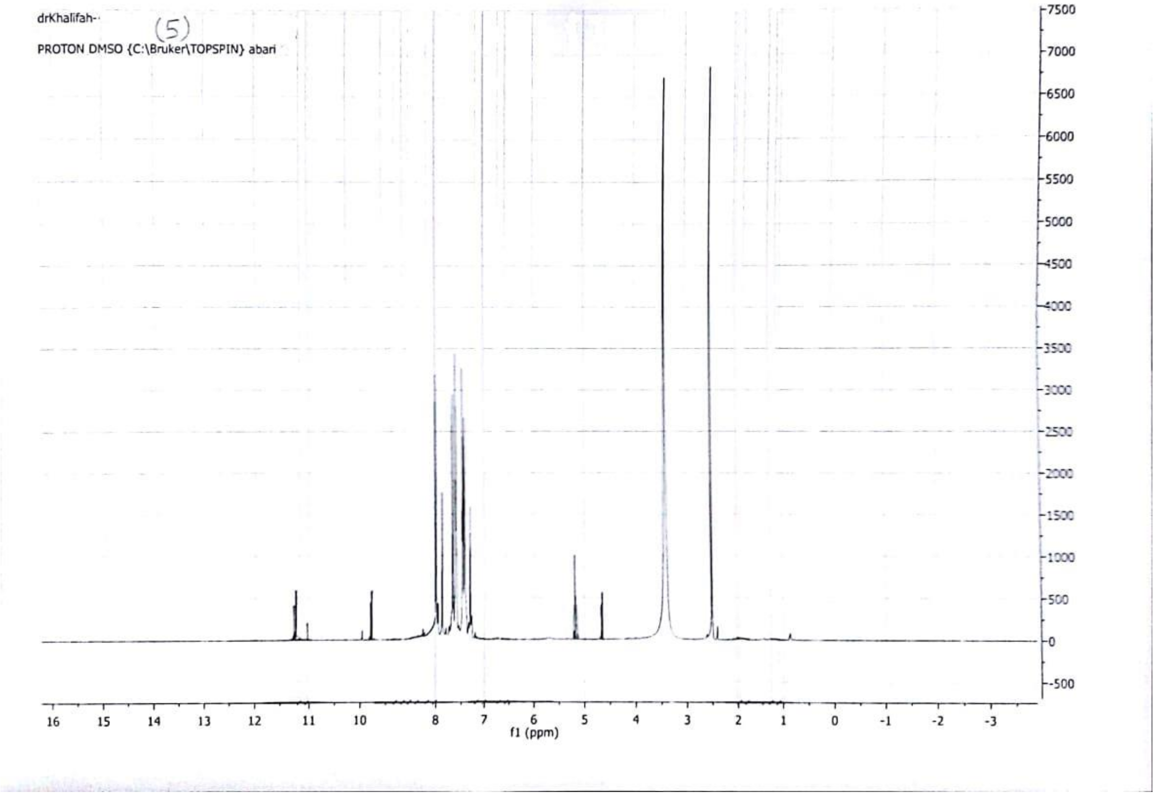
drKhalifah-
(5)
PROTON DMSO {C:\Bruker\TOPSPIN} abari
f1 (ppm)

Al-Azhar University
The Regional Center for Mycology & Biotechnology
C:\Xcalibur\data\S\EMAN-SAMIR-G12

2/15/2016 11:57:39 AM

C:\Xcalibur\methods\Mass DIP.meth

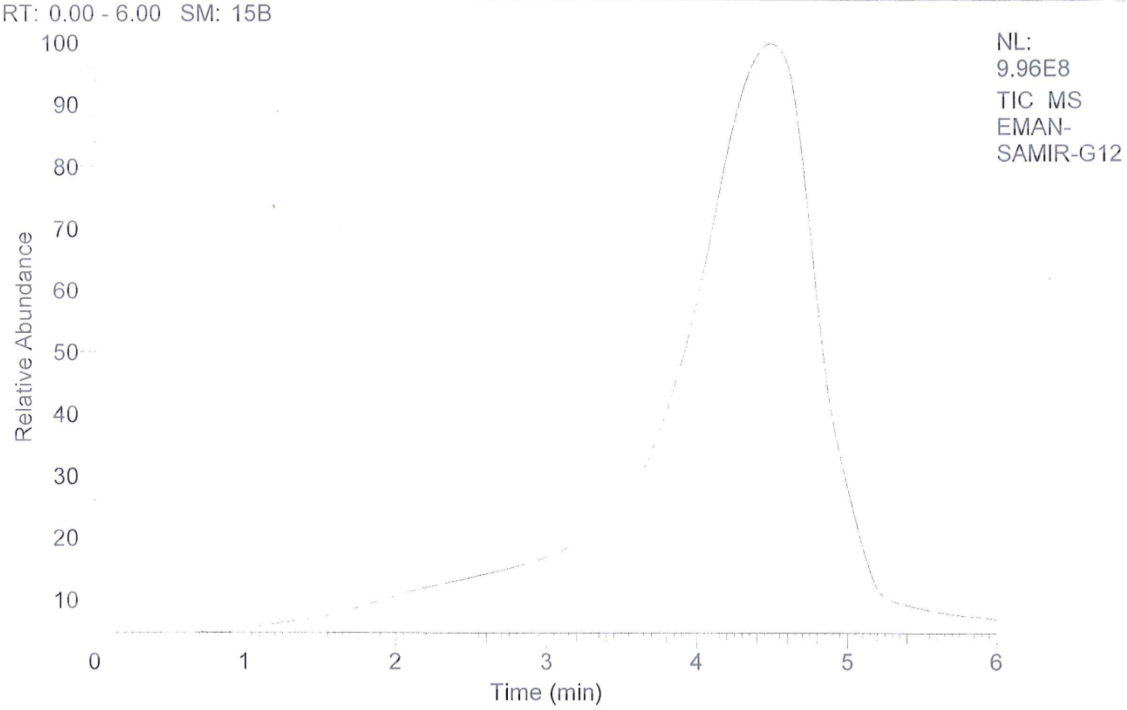

EMAN-SAMIR-G12 #201 RT: 3.38 AV: 1 SB: 18 3.30-3.40 , 3.26-3.43 NL: 8.93E5
T: {0,0} + c EI Full ms [40.00-1000.00]

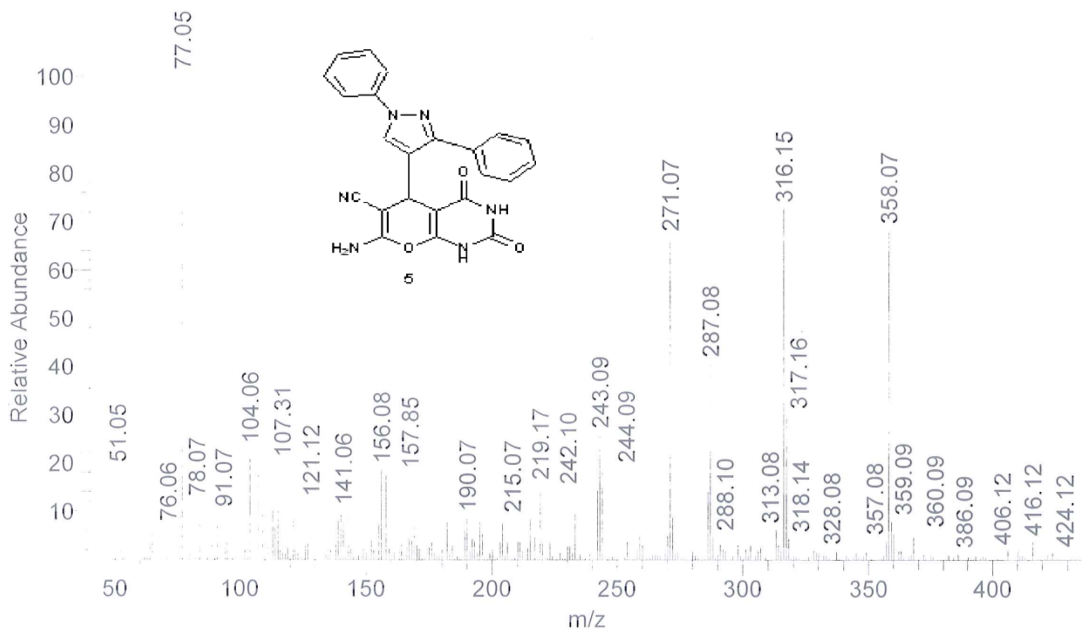

Peak Find - Memory-174

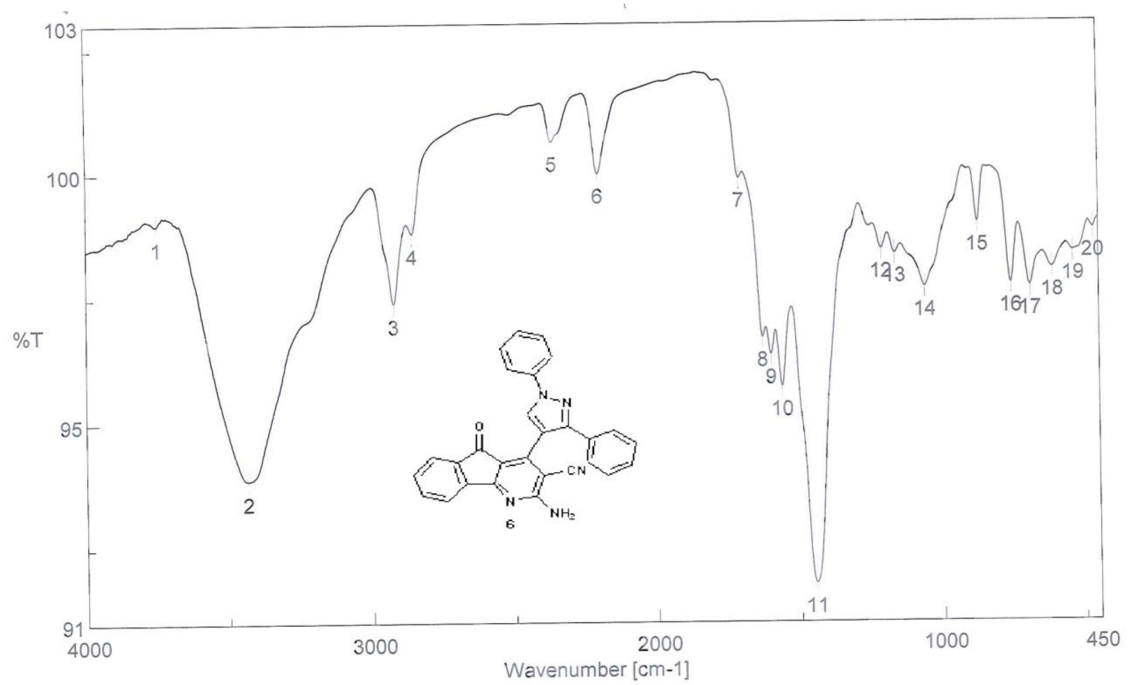

[Comments]

Sample name	G10
Comment	21/4/2016
User	IR
Division	IR
Company	MAC

[Result of Peak Picking]

No.	Position	Intensity	No.	Position	Intensity	No.	Position	Intensity
1	3751.83	98.9668	2	3434.6	93.8693	3	2923.56	97.3875
4	2857.99	98.7835	5	2363.34	100.619	6	2202.31	99.9702
7	1708.62	99.8701	8	1629.55	96.6876	9	1600.63	96.3423
10	1562.06	95.7027	11	1448.28	91.7633	12	1213.01	98.4419
13	1165.76	98.3489	14	1060.66	97.6774	15	874.56	98.965
16	757.888	97.743	17	690.391	97.688	18	613.252	98.0502
19	539.971	98.3815	20	468.617	98.8338			

Micro Analytical Center

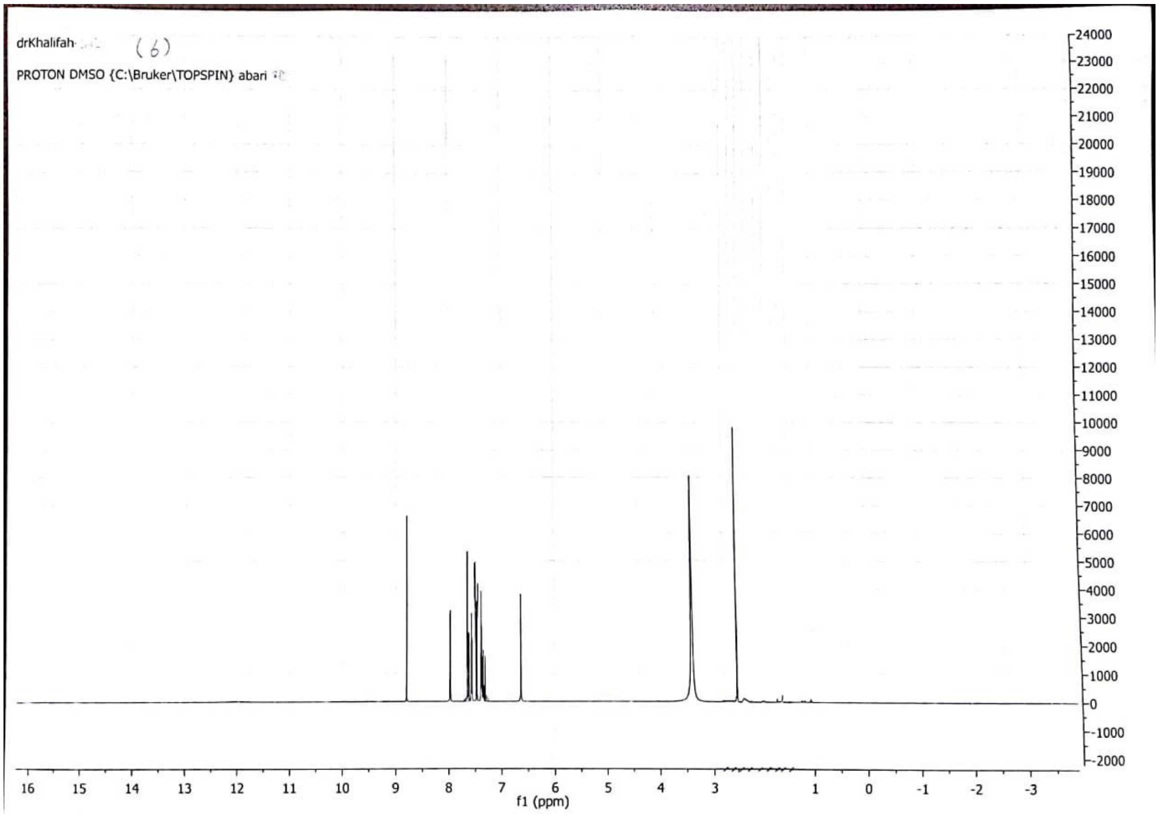
drKhalifah
(6)
PROTON DMSO {C:\Bruker\TOPSPIN} abari
f1 (ppm)

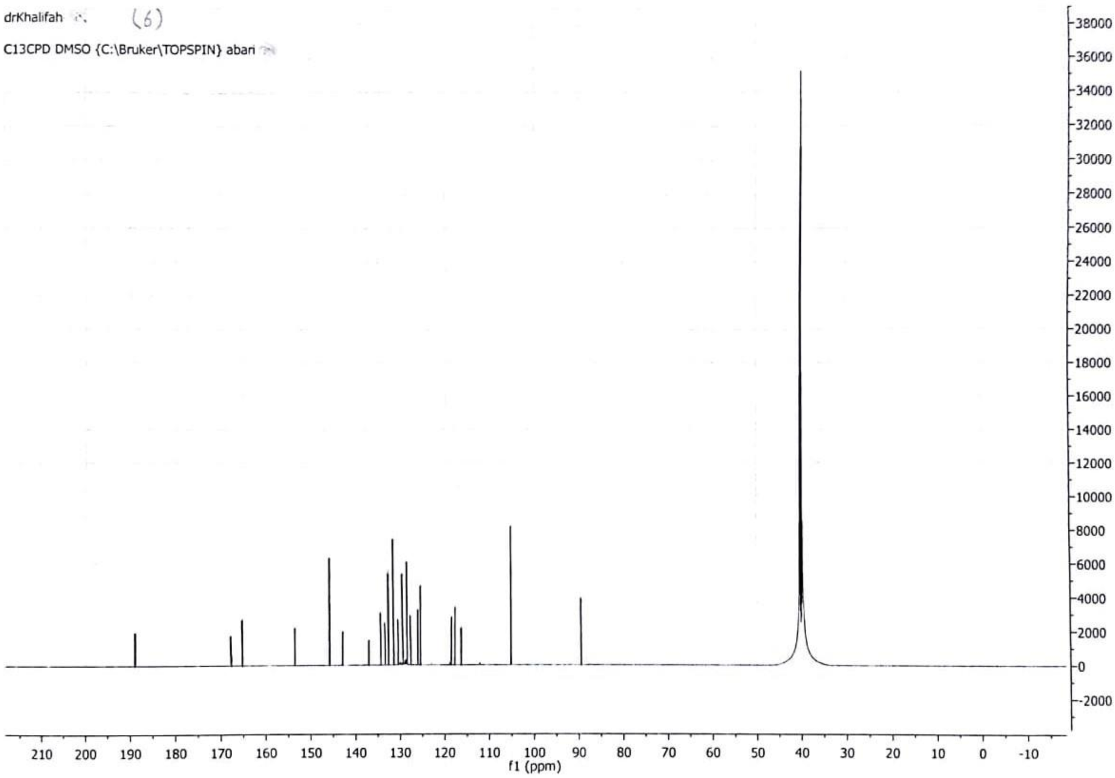
drKhalifah
(6)
C13CPD DMSO {C:\Bruker\TOPSPIN} abari
f1 (ppm)

Al-Azhar University
The Regional Center for Mycology & Biotechnology
C:\Xcalibur\data\S\EMAN-SAMIR-G10

2/15/2016 12:46:31 PM
C:\Xcalibur\methods\Mass DIP.meth

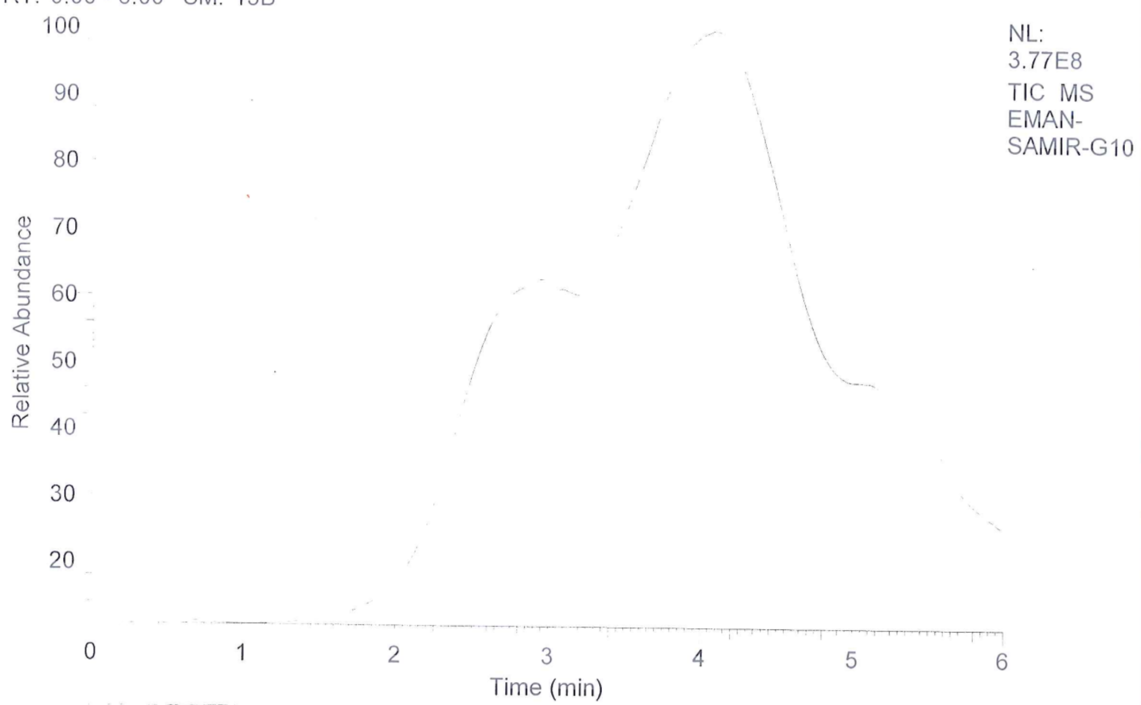

EMAN-SAMIR-G10 #294 RT: 4.94 AV: 1 SB: 2 1.86 , 1.84 NL: 1.27E7
T: {0,0} + c EI Full ms [40.00-1000.00]

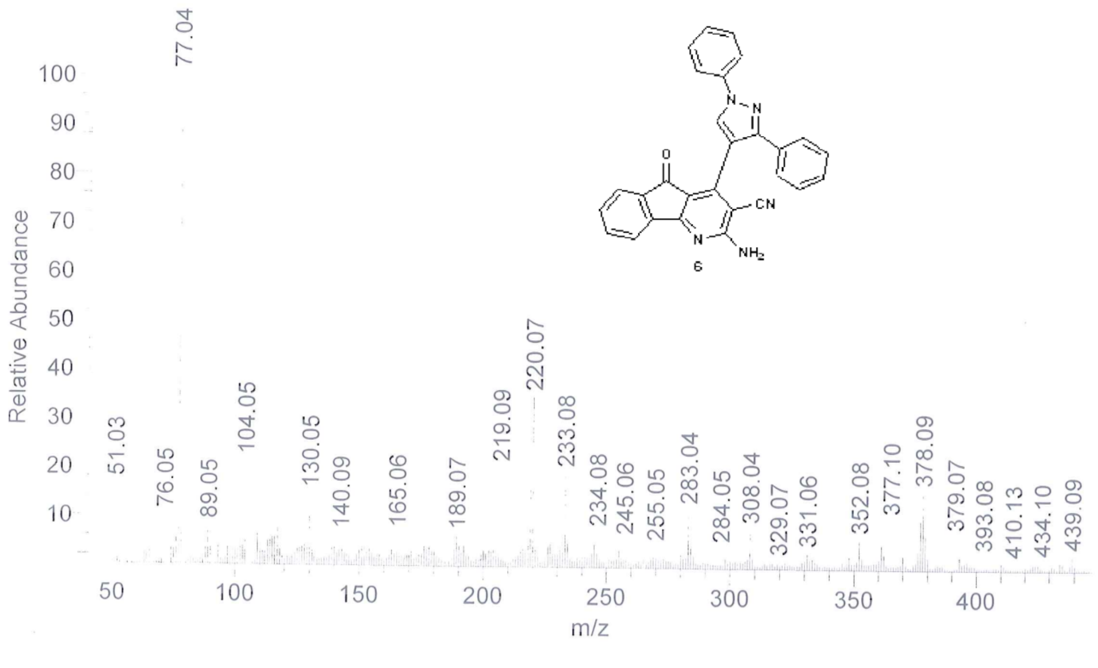

Peak Find - Memory-98

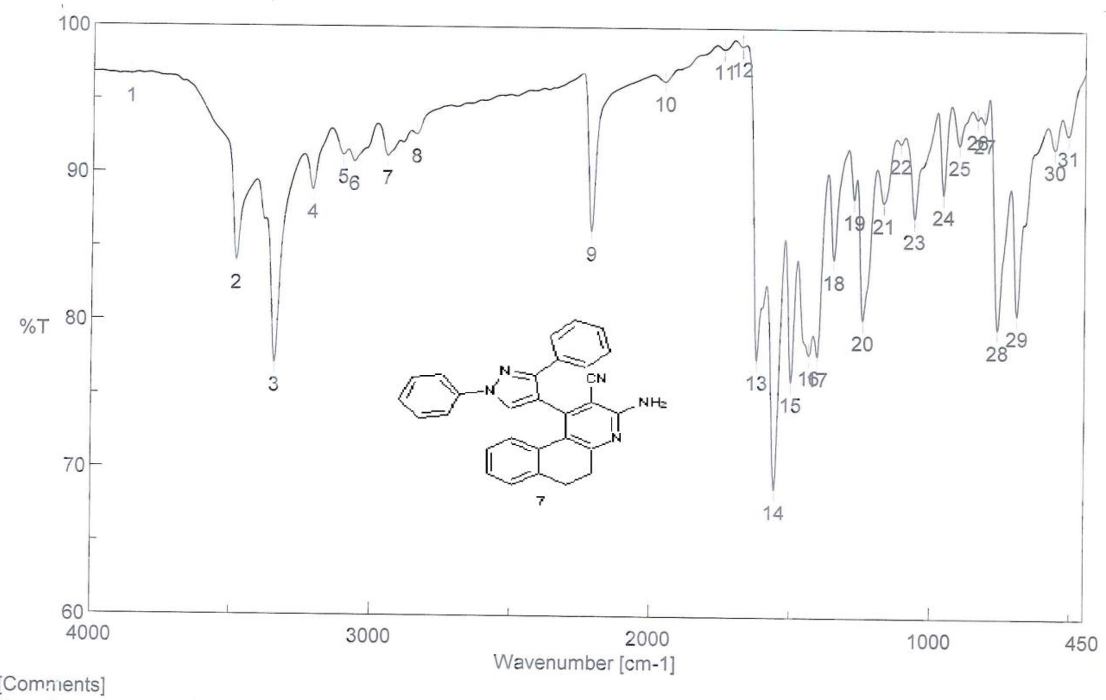

[Comments]

Sample name	G4
Comment	21/4/2016
User	IR
Division	IR
Company	MAC

[Result of Peak Picking]

No.	Position	Intensity	No.	Position	Intensity	No.	Position	Intensity
1	3864.65	96.6661	2	3483.78	84.0282	3	3345.89	77.0871
4	3212.83	88.8385	5	3104.83	91.2271	6	3065.3	90.7622
7	2946.7	91.171	8	2842.56	92.6674	9	2212.92	86.1183
10	1951.61	96.3723	11	1740.44	98.5882	12	1676.8	98.7744
13	1620.88	77.4644	14	1557.24	68.6535	15	1499.38	75.9925
16	1435.74	77.8142	17	1405.85	77.7317	18	1348	84.3082
19	1276.65	88.3956	20	1243.86	80.2168	21	1170.58	88.1689
22	1109.83	92.2325	23	1060.66	87.1371	24	956.52	88.7874
25	899.63	92.1635	26	835.026	93.9671	27	809.956	93.7017
28	760.78	79.6361	29	691.355	80.617	30	558.291	91.9191
31	511.044	92.882						

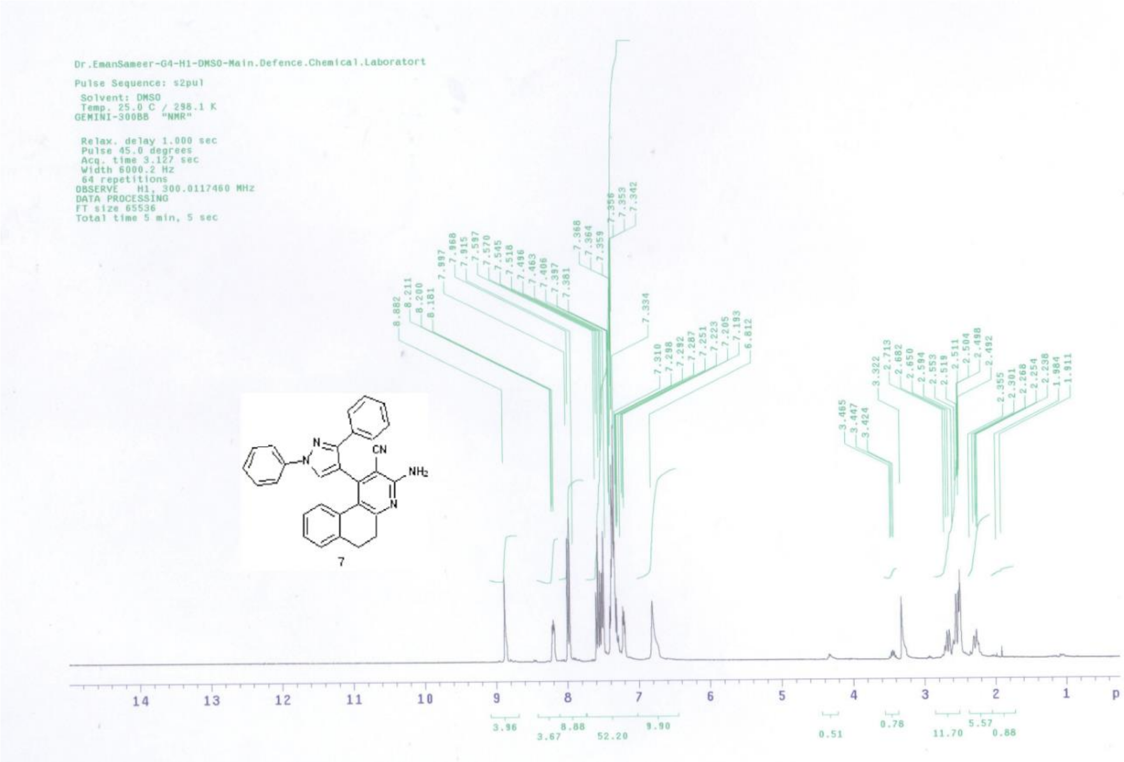
Dr.EmanSameer-G4-H1-DMSO-Main.Defence.Chemical.Laboratort
Pulse Sequence: s2pul
Solvent: DMSO
Temp. 25.0 C / 298.1 K
GEMINI-300BB "NMR"
Relax. delay 1.000 sec
Pulse 45.0 degrees
Acq. time 3.127 sec
Width 6000.2 Hz
64 repetitions
OBSERVE H1, 300.0117460 MHz
DATA PROCESSING
FT size 65536
Total time 5 min, 5 sec
CN
NH2
N
7
14
13
12
11
10
9
8
7
6
5
4
3
2
1

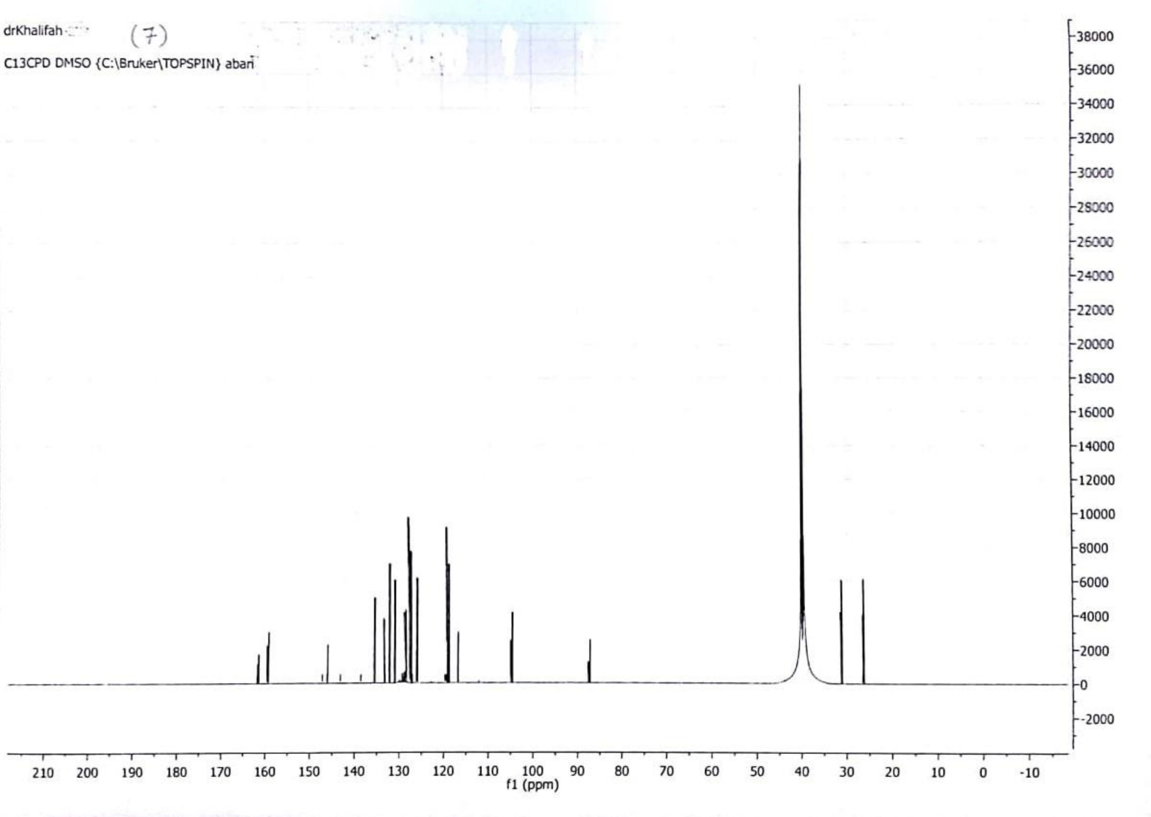
drKhalifah
(7)
C13CPD DMSO {C:\Bruker\TOPSPIN} abari
f1 (ppm)

Al-Azhar University
The Regional Center for Mycology & Biotechnology
C:\Xcalibur\data\S\EMAN-SAMIR-G4

2/15/2016 10:46:21 AM
C:\Xcalibur\methods\Mass DIP.meth

RT: 0.00 - 6.00 SM: 15B

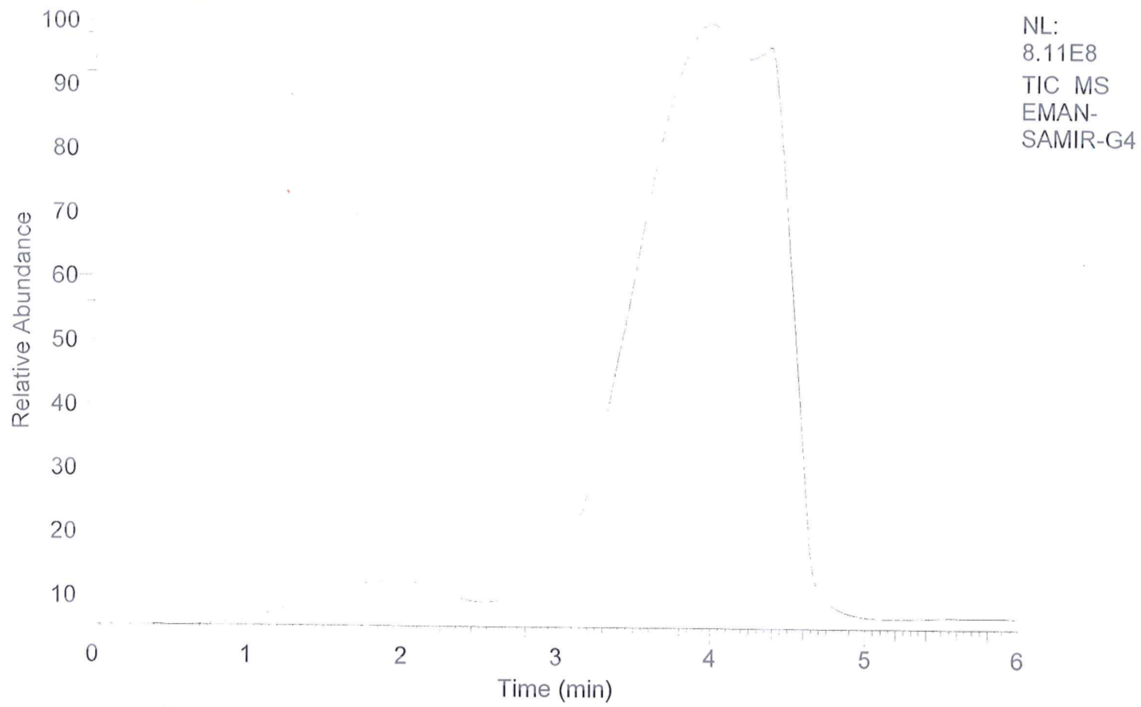

EMAN-SAMIR-G4 #270 RT: 4.54 AV: 1 NL: 2.23E7
T: {0,0} + c EI Full ms [40.00-1000.00]

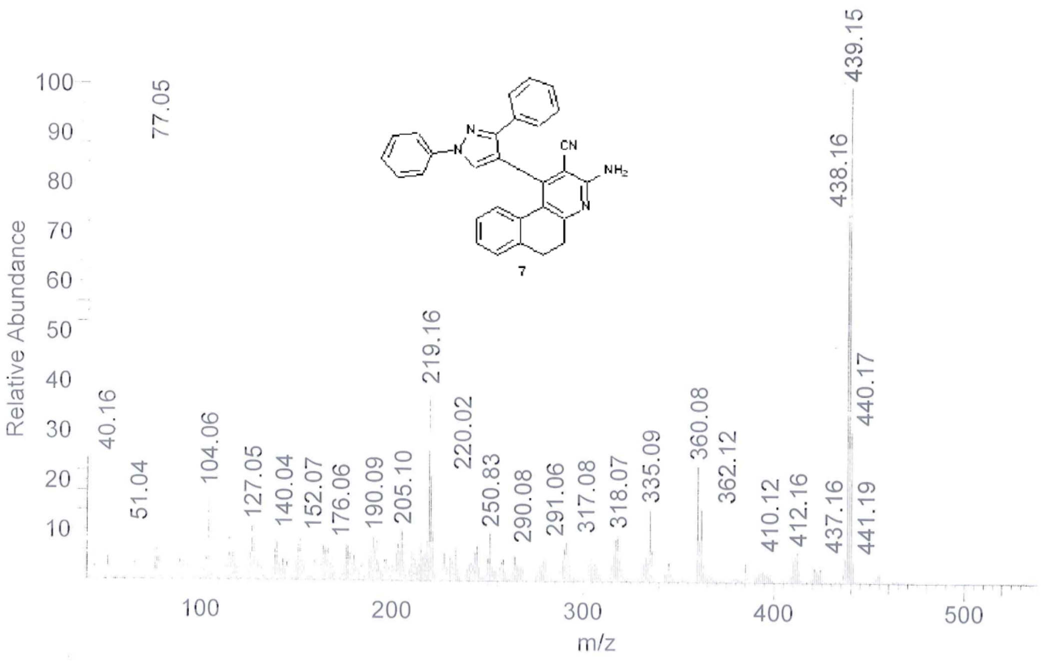

Peak Find - Memory-102

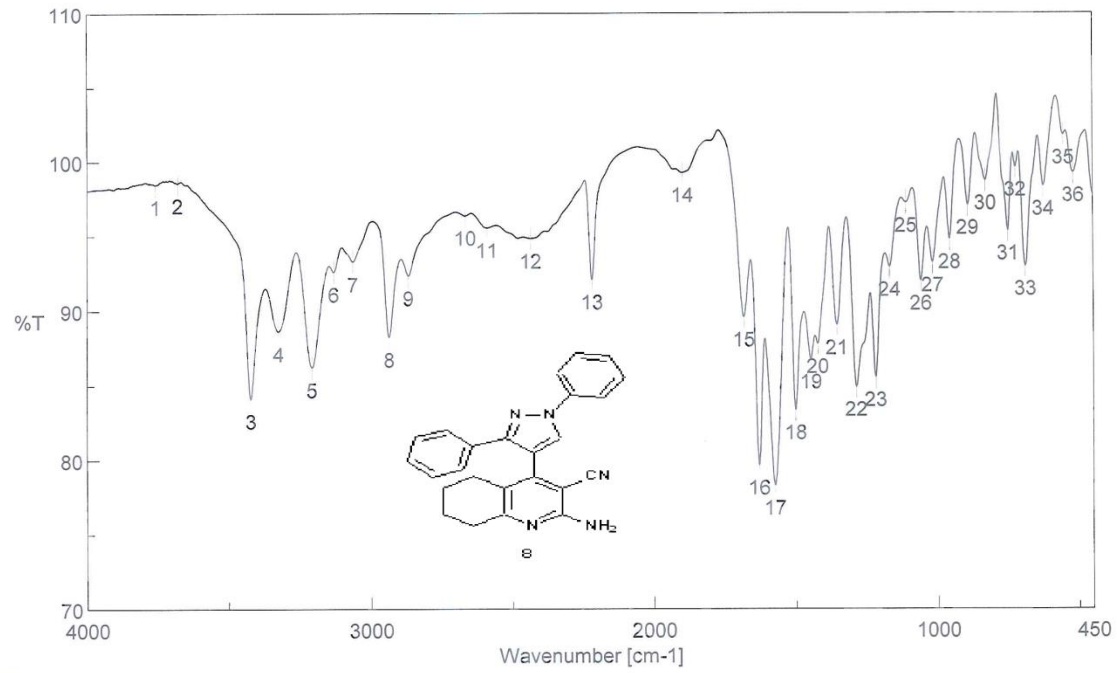

[Comments]

Sample name	G7
Comment	21/4/2016
User	IR
Division	IR
Company	MAC

[Result of Peak Picking]

No.	Position	Intensity	No.	Position	Intensity	No.	Position	Intensity
1	3757.62	98.4505	2	3678.55	98.5578	3	3423.03	84.1216
4	3325.64	88.6178	5	3208	86.2213	6	3130.87	92.6135
7	3063.37	93.287	8	2935.13	88.2266	9	2866.67	92.3558
10	2665.14	96.3623	11	2588.97	95.5373	12	2433.73	94.8338
13	2216.77	92.0893	14	1896.65	99.2388	15	1682.59	89.5624
16	1629.55	79.6279	17	1574.59	78.2739	18	1501.31	83.3392
19	1447.31	86.7582	20	1423.21	87.7842	21	1355.71	89.0326
22	1287.25	84.9004	23	1218.79	85.5534	24	1169.62	92.9238
25	1110.8	97.2065	26	1058.73	92.016	27	1017.27	93.225
28	956.52	94.7747	29	891.916	97.0533	30	829.241	98.7019
31	750.174	95.3494	32	723.175	99.5927	33	688.463	92.9855
34	624.823	98.3283	35	553.47	101.768	36	518.758	99.2302

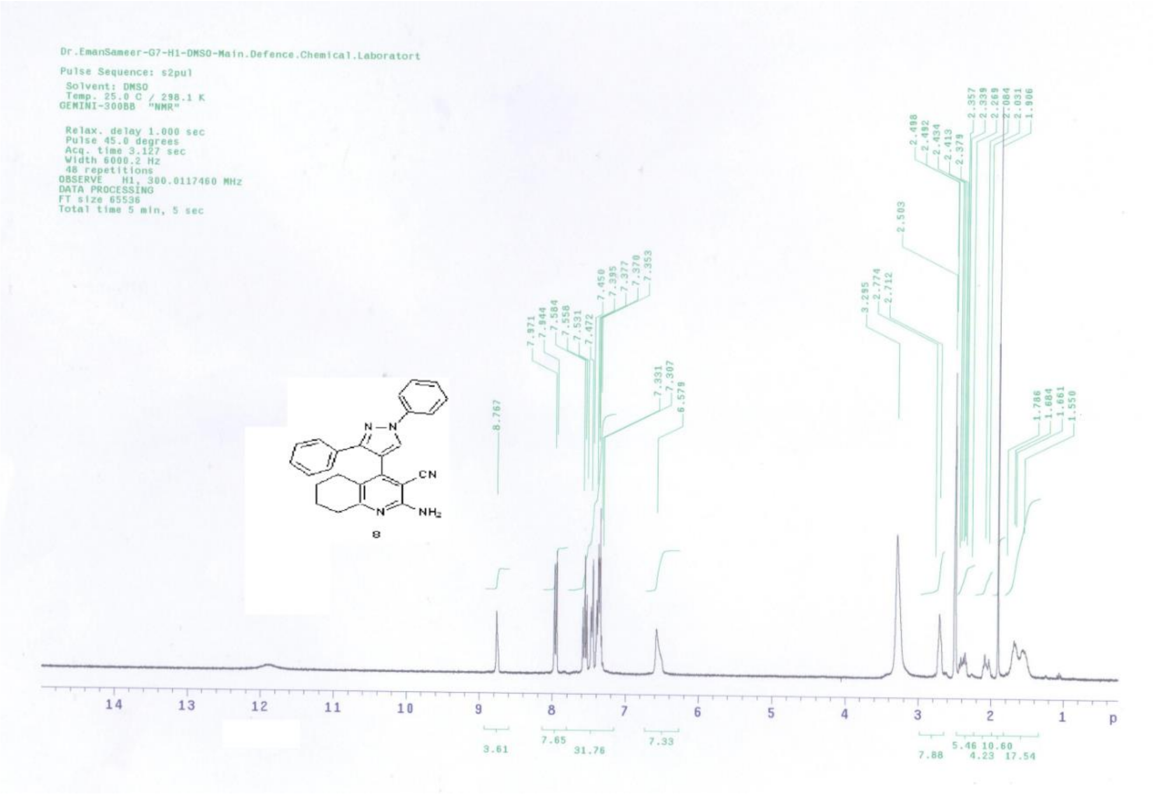
Dr.EmanSameer-G7-H1-DMSO-Main.Defence.Chemical.Laboratort
Pulse Sequence: s2pul
Solvent: DMSO
Temp. 25.0 C / 298.1 K
GEMINI-300BB "NMR"
Relax. delay 1.000 sec
Pulse 45.0 degrees
Acq. time 3.127 sec
Width 6000.2 Hz
48 repetitions
OBSERVE H1, 300.0117460 MHz
DATA PROCESSING
FT size 65536
Total time 5 min, 5 sec
8.767
7.971
7.944
7.584
7.558
7.531
7.472
7.450
7.395
7.377
7.370
7.353
7.331
7.307
6.578
3.295
2.774
2.712
2.503
2.498
2.492
2.434
2.413
2.379
2.357
2.339
2.269
2.084
2.031
1.906
1.786
1.684
1.661
1.559
CN
NH2
N
N-N
8
3.61
7.65
31.76
7.33
7.88
5.46
10.60
4.23
17.54

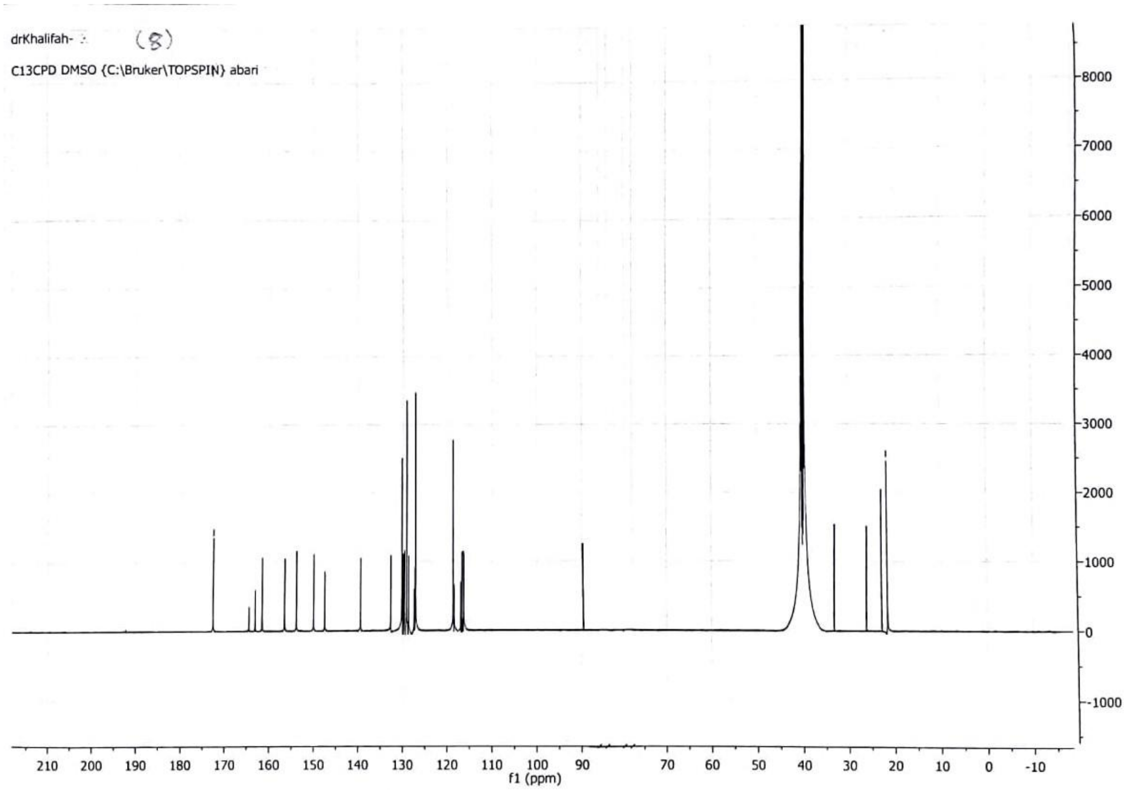
drKhalifah-
(8)
C13CPD DMSO {C:\Bruker\TOPSPIN} abari
f1 (ppm)

Al-Azhar University
The Regional Center for Mycology & Biotechnology
C:\Xcalibur\data\S\EMAN-SAMIR-G7

2/15/2016 3:02:13 PM
C:\Xcalibur\methods\Mass DIP.meth

RT: 0.00 - 6.00 SM: 15B

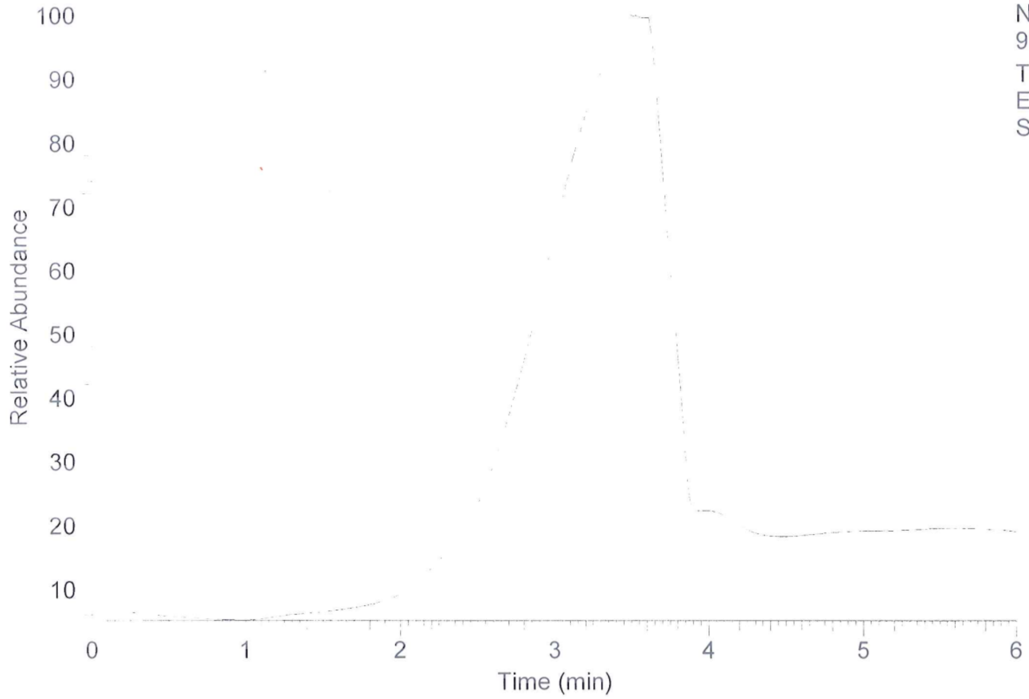

EMAN-SAMIR-G7 #211 RT: 3.55 AV: 1 NL: 1.71E8
T: {0,0} + c EI Full ms [40.00-1000.00]

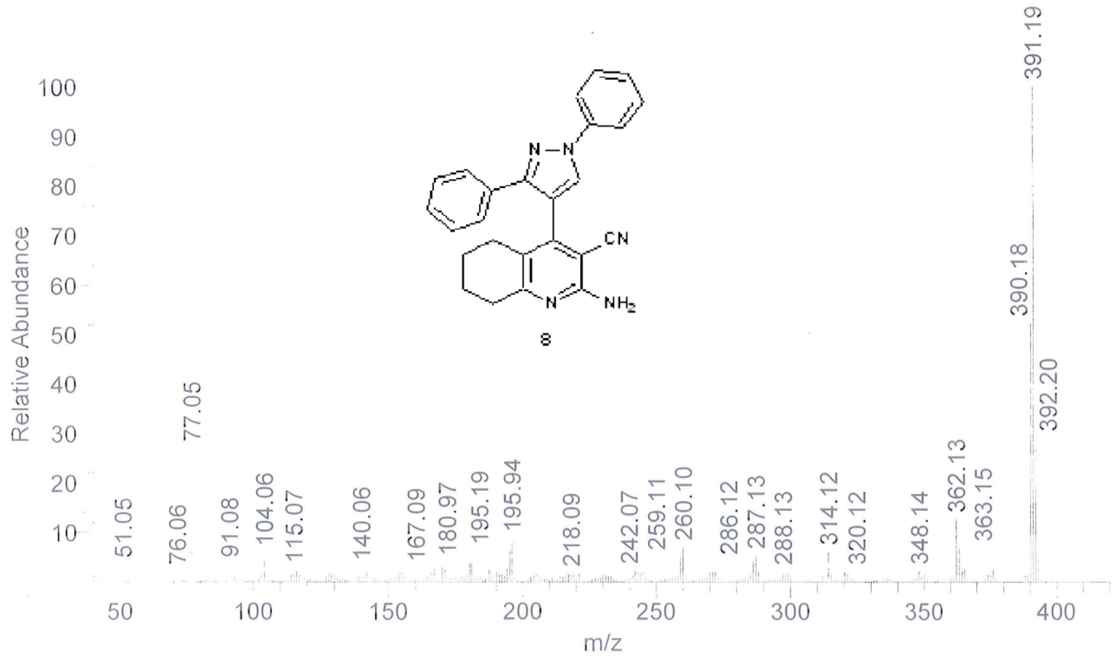

Peak Find - Memory-141

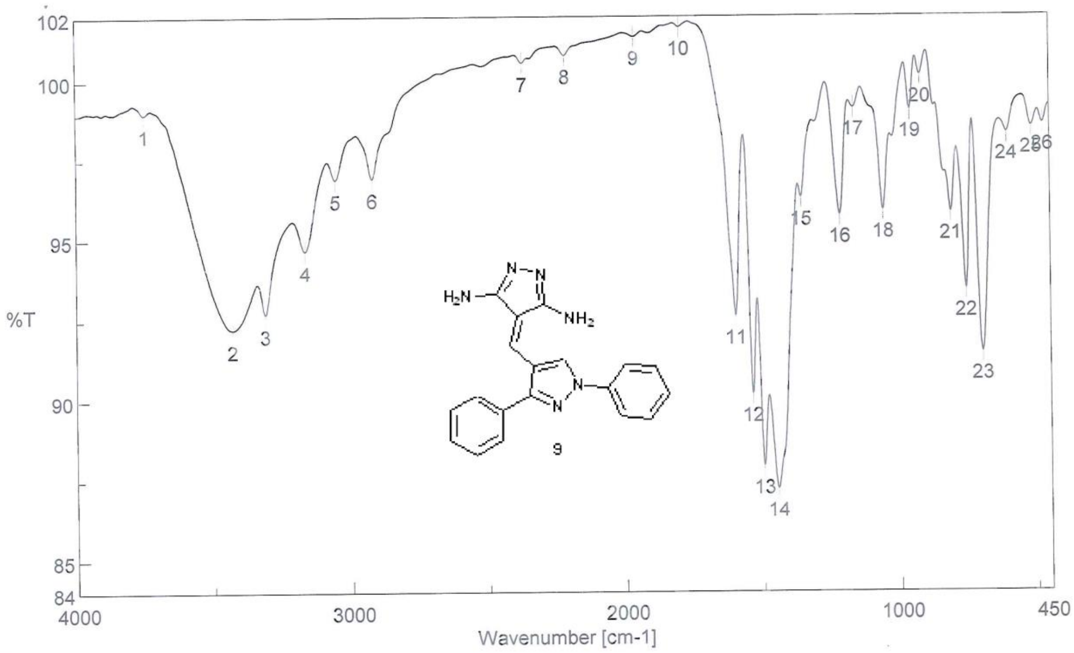

[Comments]

Sample name	G1
Comment	21/4/2016
User	IR
Division	IR
Company	MAC

[Result of Peak Picking]

No.	Position	Intensity	No.	Position	Intensity	No.	Position	Intensity
1	3750.87	98.9651	2	3431.71	92.2306	3	3313.11	92.7043
4	3167.51	94.6648	5	3056.62	96.8899	6	2921.63	96.9141
7	2370.09	100.549	8	2213.88	100.785	9	1961.25	101.361
10	1796.37	101.665	11	1597.73	92.6184	12	1536.99	90.1901
13	1498.42	87.9359	14	1448.28	87.2197	15	1358.6	96.2877
16	1216.86	95.7459	17	1165.76	99.105	18	1058.73	95.8928
19	958.448	99.0415	20	920.843	100.129	21	810.92	95.8327
22	756.923	93.4397	23	697.141	91.4827	24	605.539	98.309
25	515.865	98.503	26	475.367	98.5915			

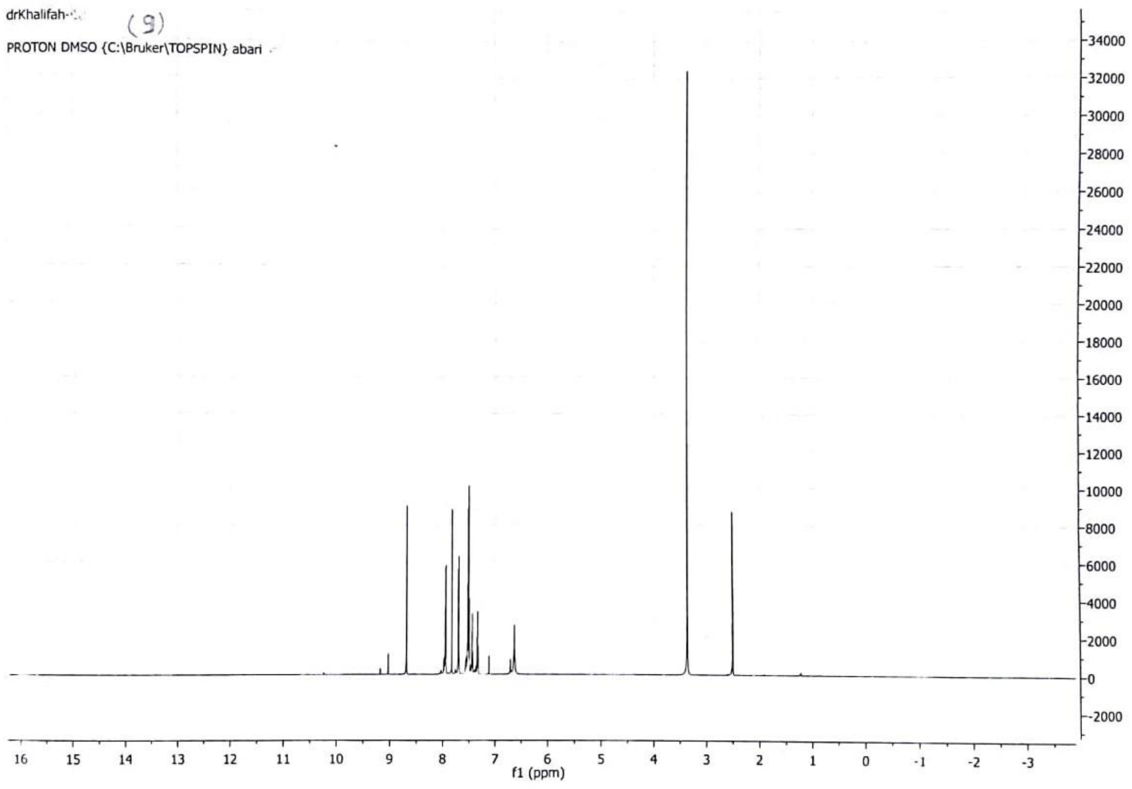
drKhalifah-9
(9)
PROTON DMSO {C:\Bruker\TOPSPIN} abari
f1 (ppm)

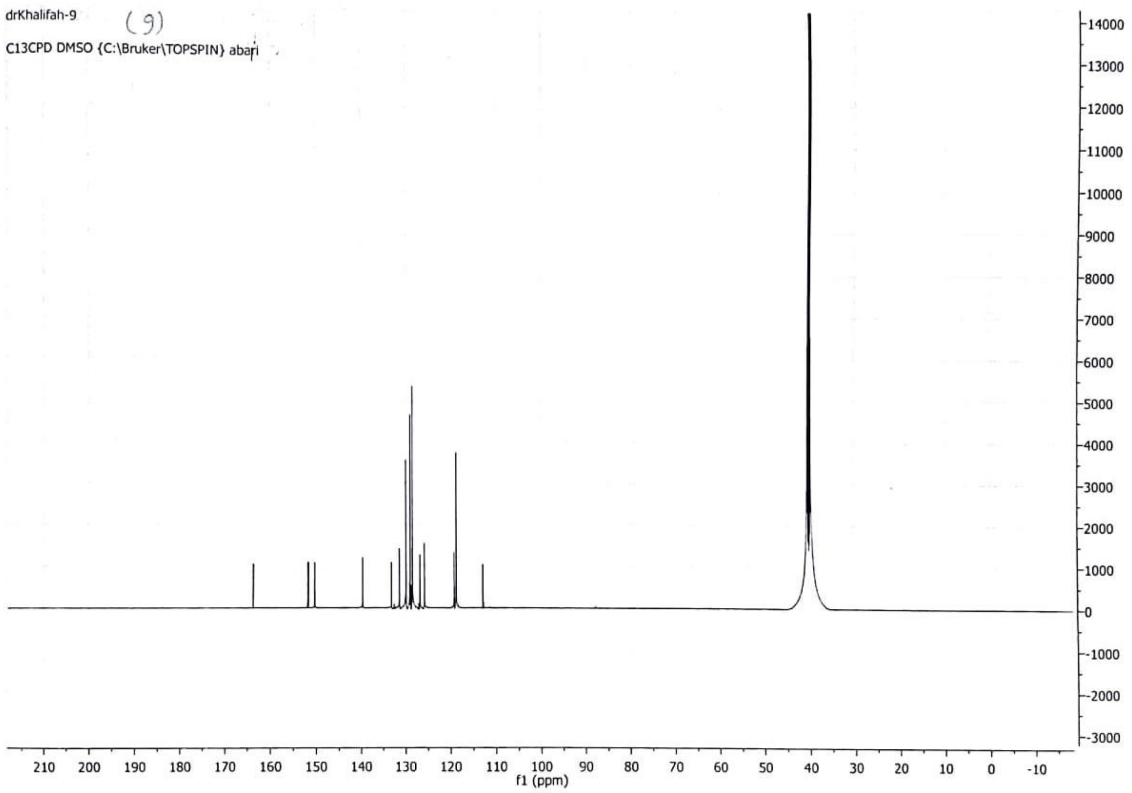
drKhalifah-9
(9)
C13CPD DMSO {C:\Bruker\TOPSPIN} abari
f1 (ppm)

Al-Azhar University
The Regional Center for Mycology & Biotechnology
C:\Xcalibur\data\S\EMAN-SAMIR-G1

2/16/2016 9:17:45 AM
C:\Xcalibur\methods\Mass DIP.meth

RT: 0.00 - 6.00 SM: 15B

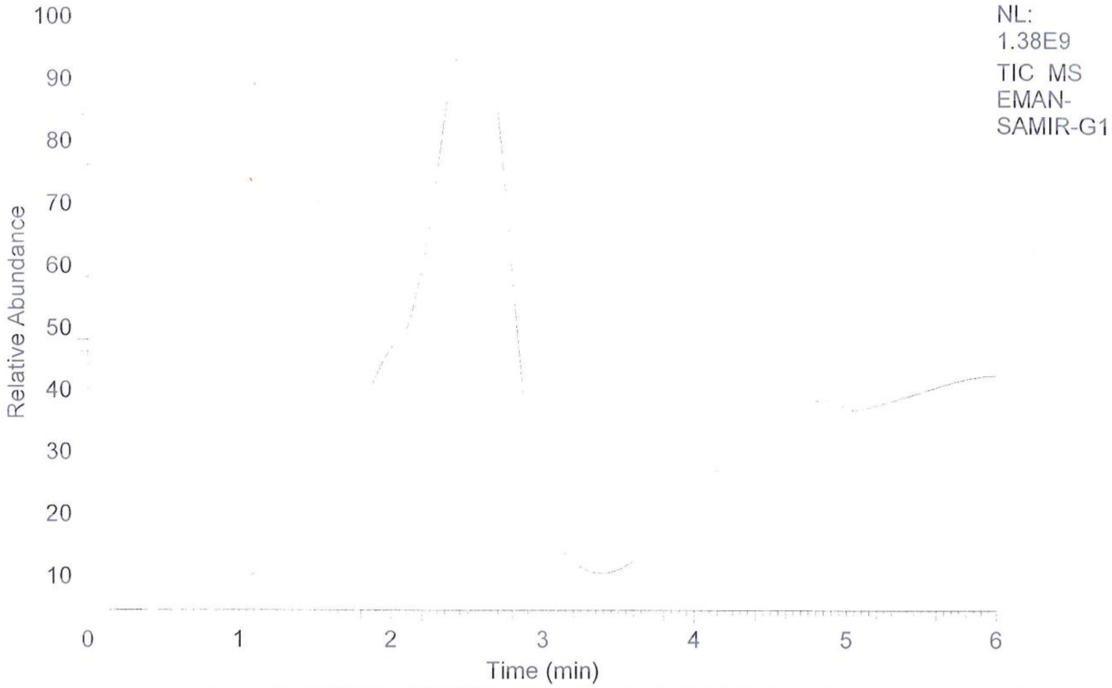

EMAN-SAMIR-G1 #326 RT: 5.47 AV: 1 SB: 2 5.44 , 5.44 NL: 1.45E6
T: {0,0} + c EI Full ms [40.00-1000.00]

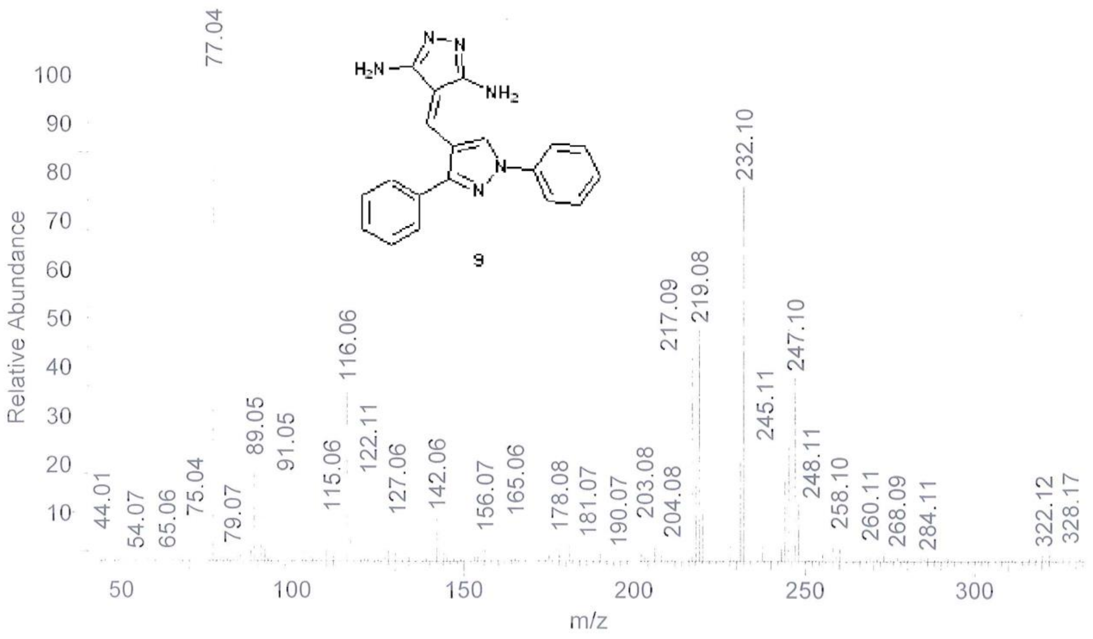

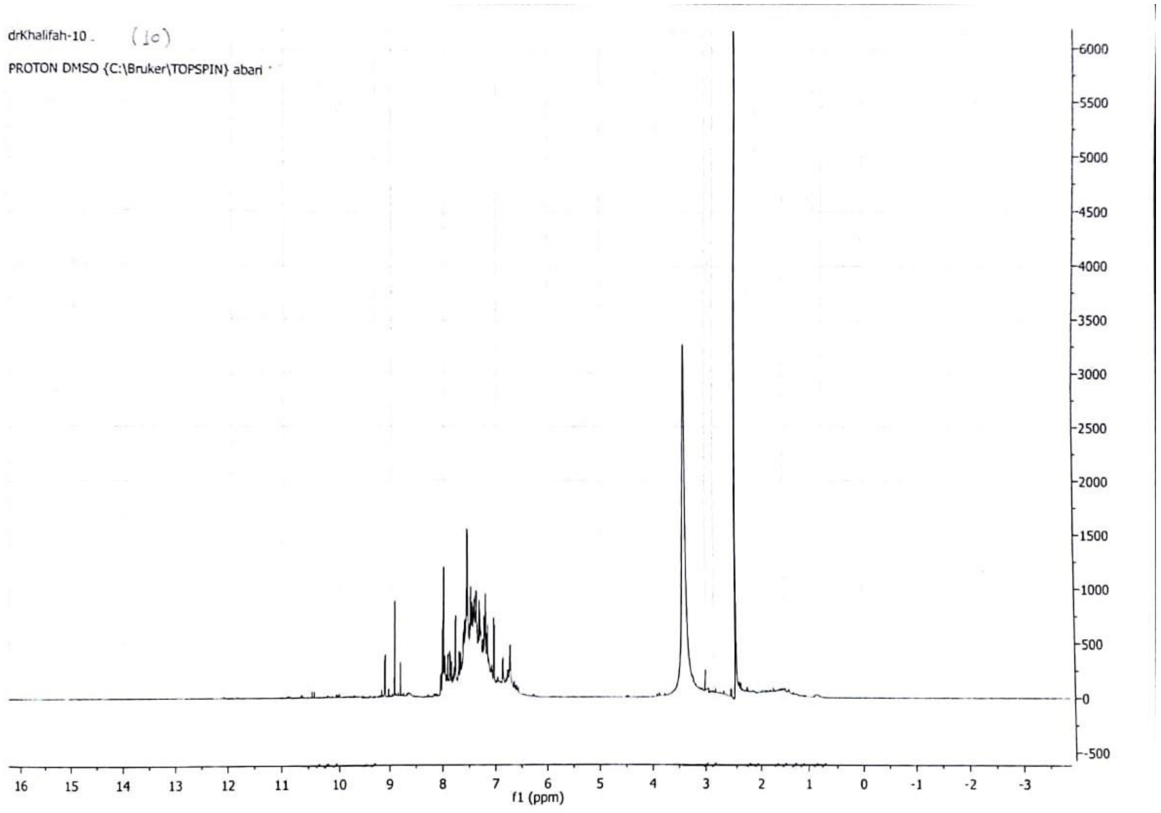
drKhalifah-10
(10)
PROTON DMSO {C:\Bruker\TOPSPIN} abari
f1 (ppm)

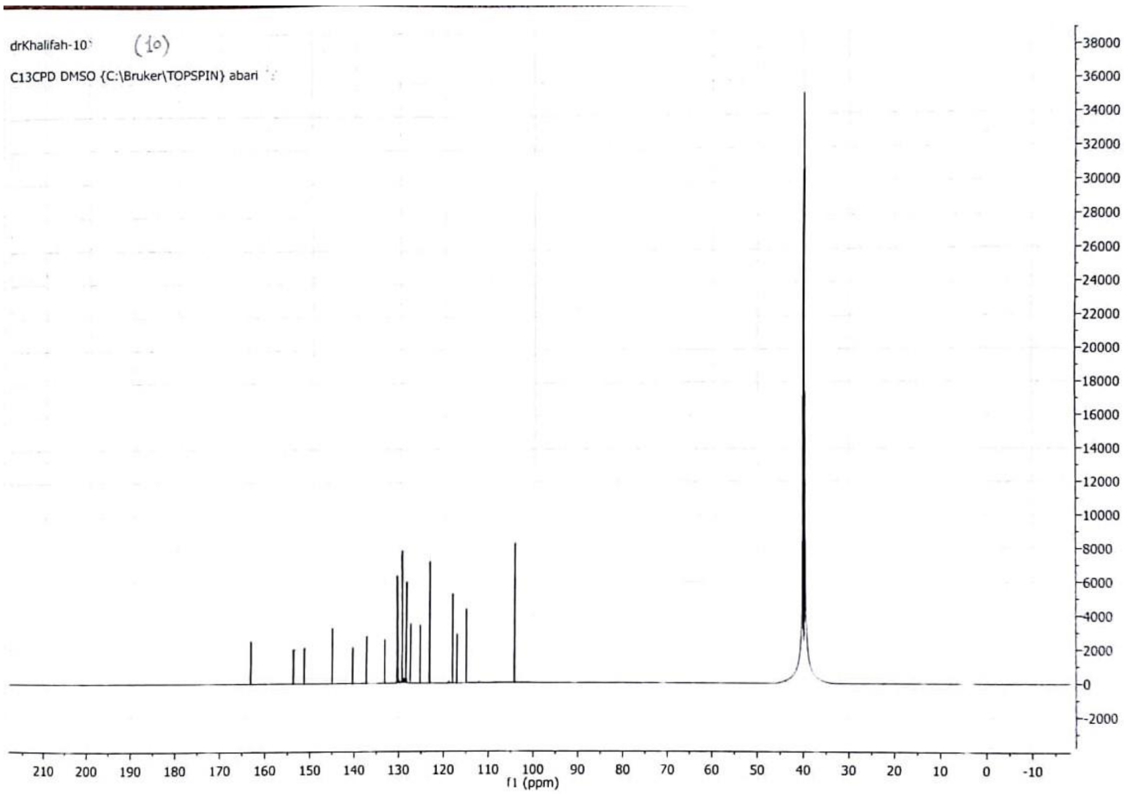
drKhalifah-10
(10)
C13CPD DMSO {C:\Bruker\TOPSPIN} abari
f1 (ppm)

Peak Find - Memory-149

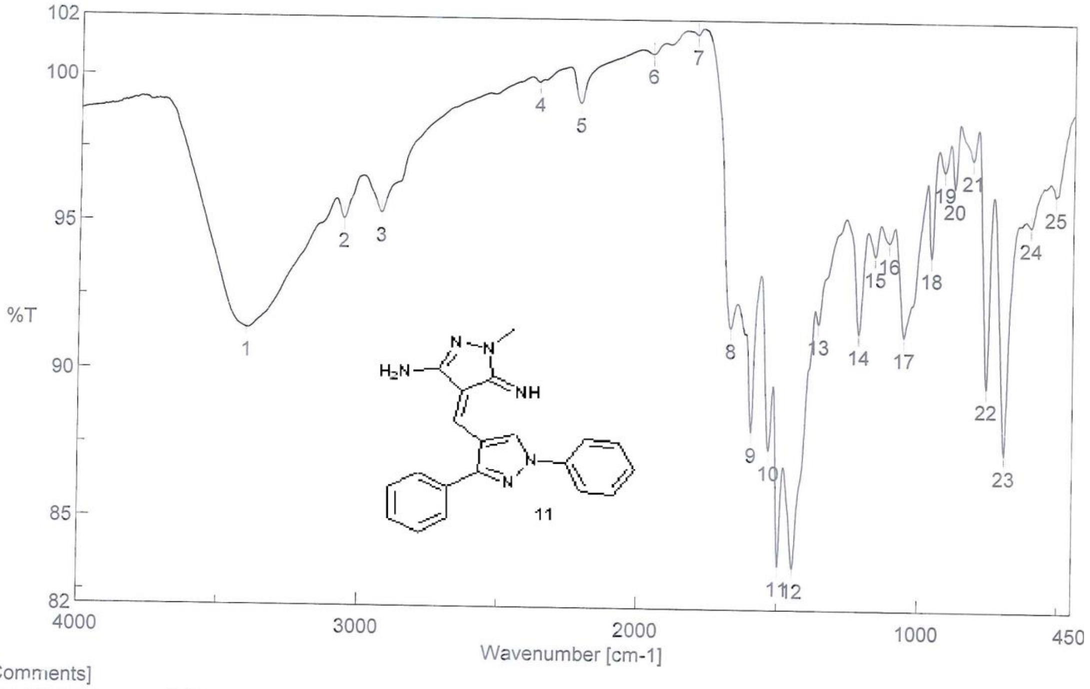

[Comments]

Sample name	G11
Comment	21/4/2016
User	IR
Division	IR
Company	MAC

[Result of Peak Picking]

No.	Position	Intensity	No.	Position	Intensity	No.	Position	Intensity
1	3401.82	91.3924	2	3058.55	95.1081	3	2925.48	95.3173
4	2361.41	99.8349	5	2213.88	99.1432	6	1956.43	100.842
7	1798.3	101.511	8	1671.02	91.5335	9	1596.77	88.0316
10	1534.1	87.4182	11	1498.42	83.4907	12	1447.31	83.4253
13	1359.57	91.7101	14	1215.9	91.3849	15	1159.01	94.0159
16	1108.87	94.4742	17	1053.91	91.2938	18	957.484	93.9936
19	913.129	96.9038	20	876.488	96.3404	21	810.92	97.3115
22	757.888	89.598	23	693.284	87.3399	24	601.682	95.0513
25	514.901	96.109						

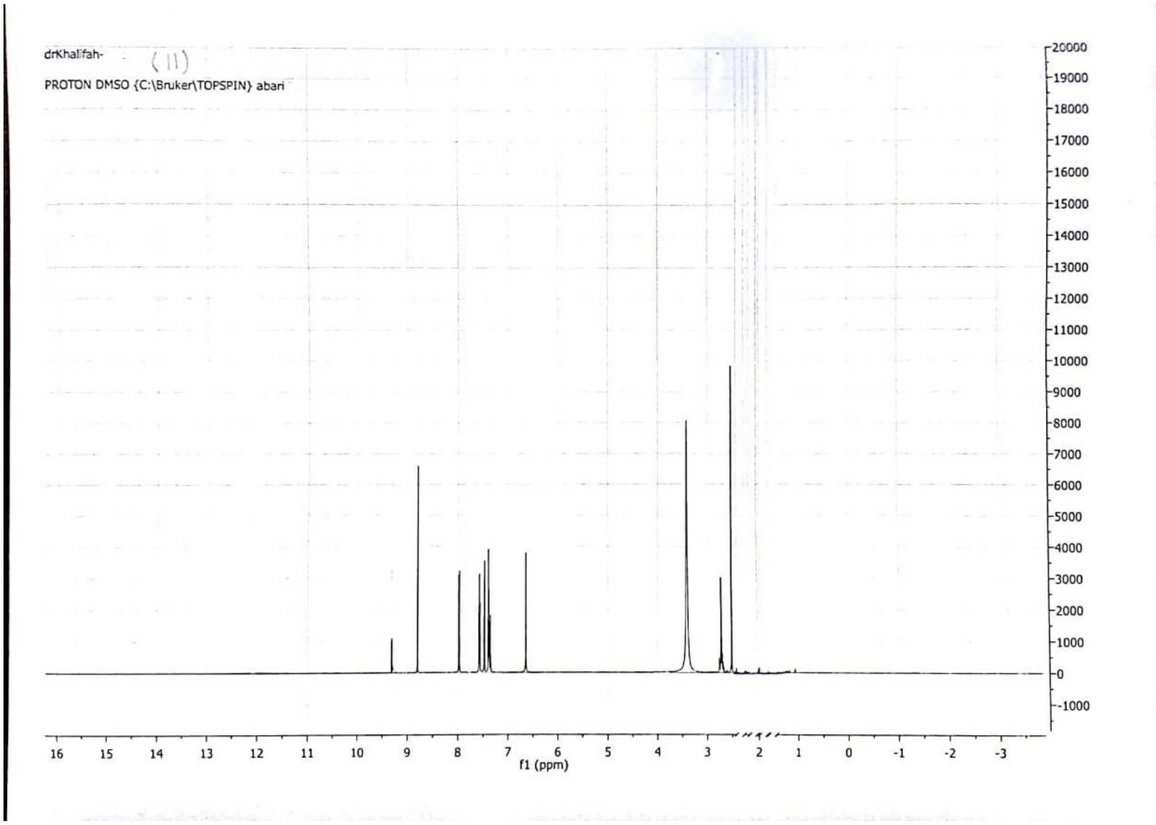
drKhalifah-
(11)
PROTON DMSO {C:\Bruker\TOPSPIN} abari
f1 (ppm)

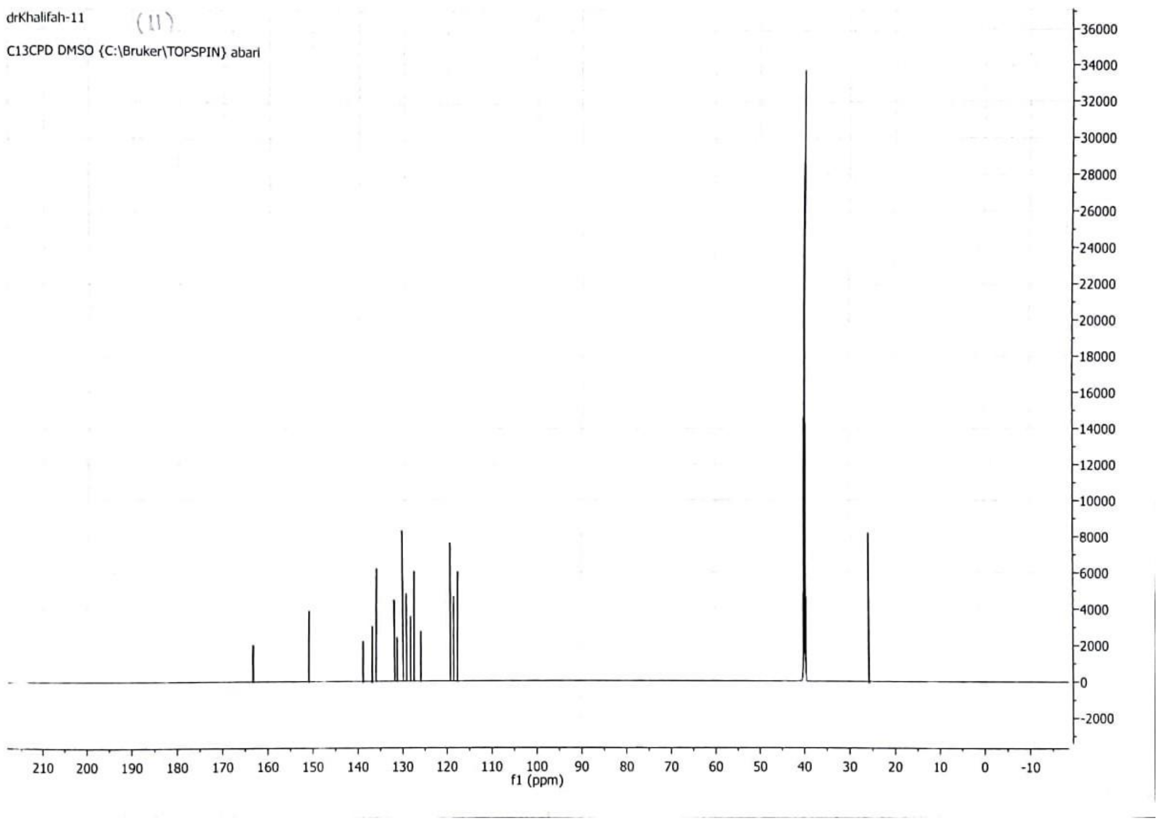
drKhalifah-11
(11)
C13CPD DMSO {C:\Bruker\TOPSPIN} abari
f1 (ppm)

Peak Find - Memory-136

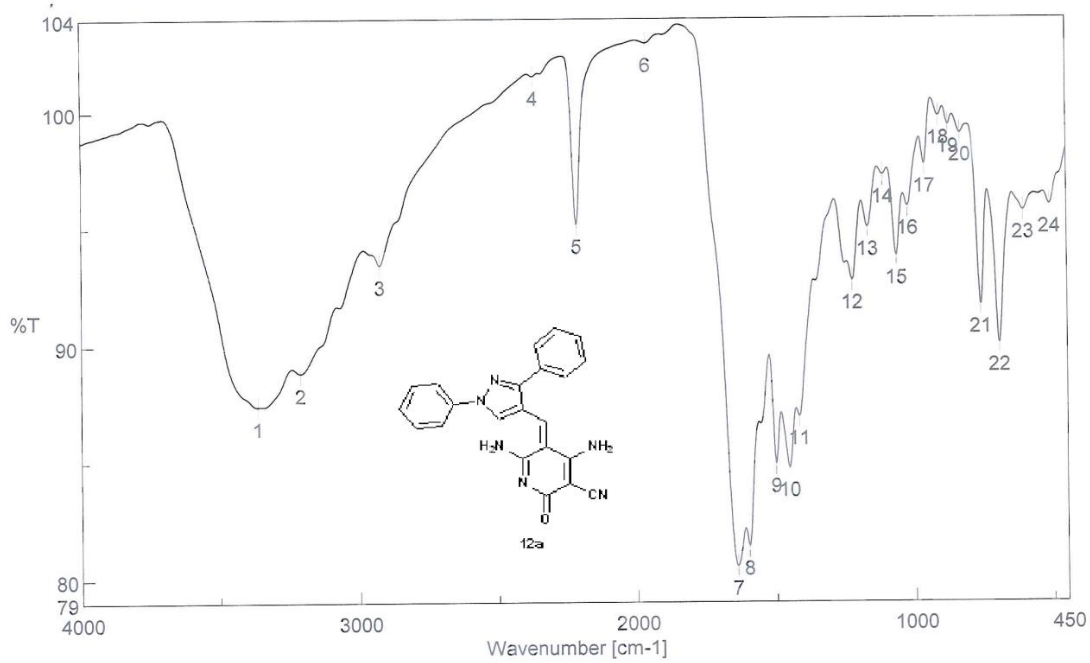

[Comments]

Sample name	G3
Comment	21/4/2016
User	IR
Division	IR
Company	MAC

[Result of Peak Picking]

No.	Position	Intensity	No.	Position	Intensity	No.	Position	Intensity
1	3366.14	87.4095	2	3211.86	88.831	3	2925.48	93.426
4	2368.16	101.517	5	2213.88	95.151	6	1959.32	102.941
7	1641.13	80.526	8	1599.66	81.3683	9	1501.31	84.896
10	1453.1	84.7202	11	1417.42	86.9229	12	1224.58	92.7274
13	1167.69	94.999	14	1112.73	97.2351	15	1064.51	93.7724
16	1023.05	95.8862	17	961.341	97.6838	18	909.272	99.7589
19	874.56	99.3832	20	831.169	99.0174	21	759.816	91.6832
22	693.284	90.0501	23	605.539	95.6931	24	511.044	95.9461

Micro Analytical Center

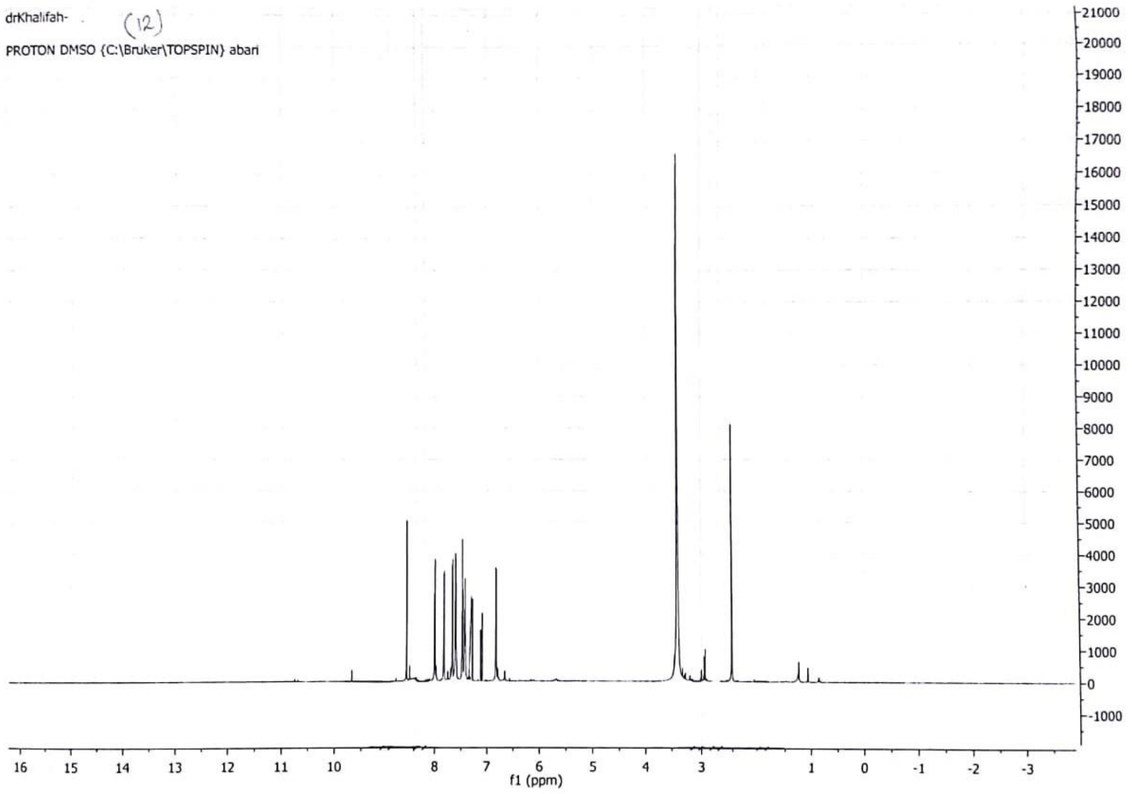
drKhalifah- (12)
PROTON DMSO {C:\Bruker\TOPSPIN} abari
f1 (ppm)

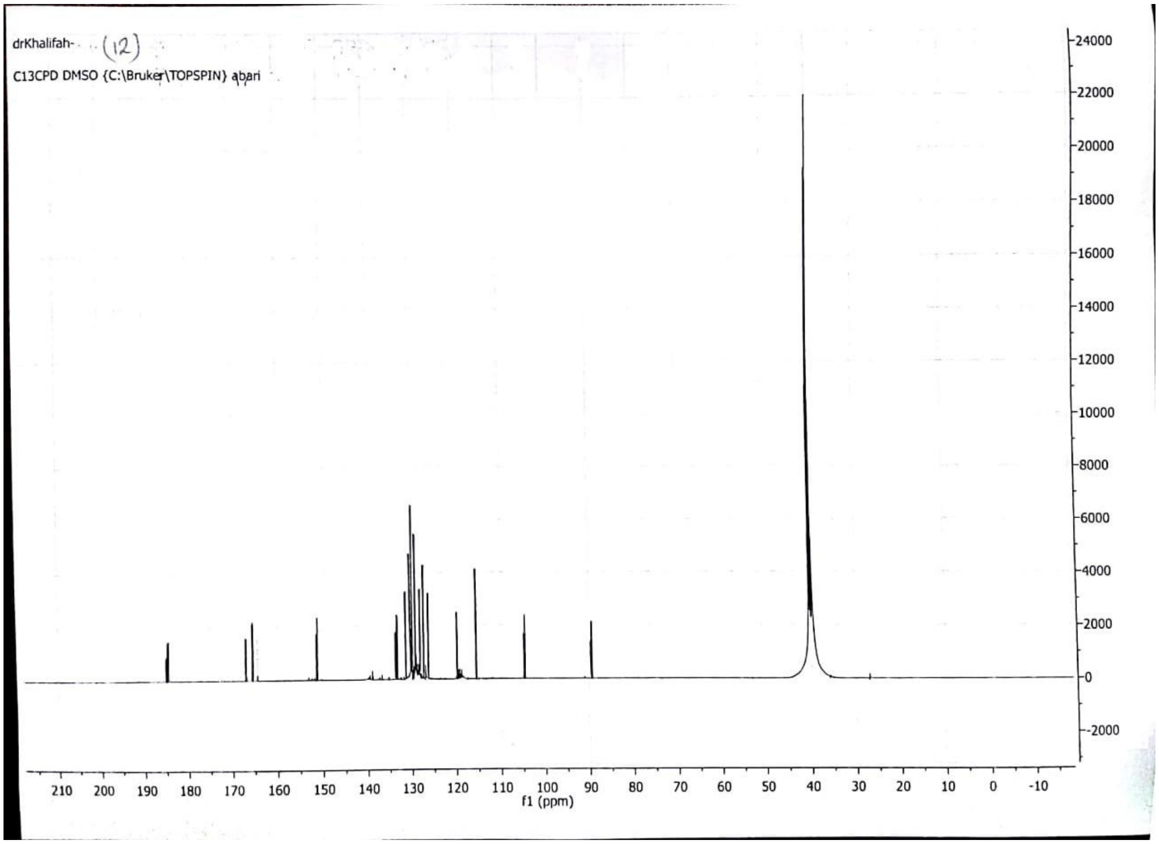
drKhalifah- (12)
C13CPD DMSO {C:\Bruker\TOPSPIN} abari
f1 (ppm)

Al-Azhar University
The Regional Center for Mycology & Biotechnology
C:\Xcalibur\data\S\EMAN-SAMIR-G3

2/15/2016 12:36:27 PM
C:\Xcalibur\methods\Mass DIP.meth

RT: 0.00 - 6.00 SM: 15B

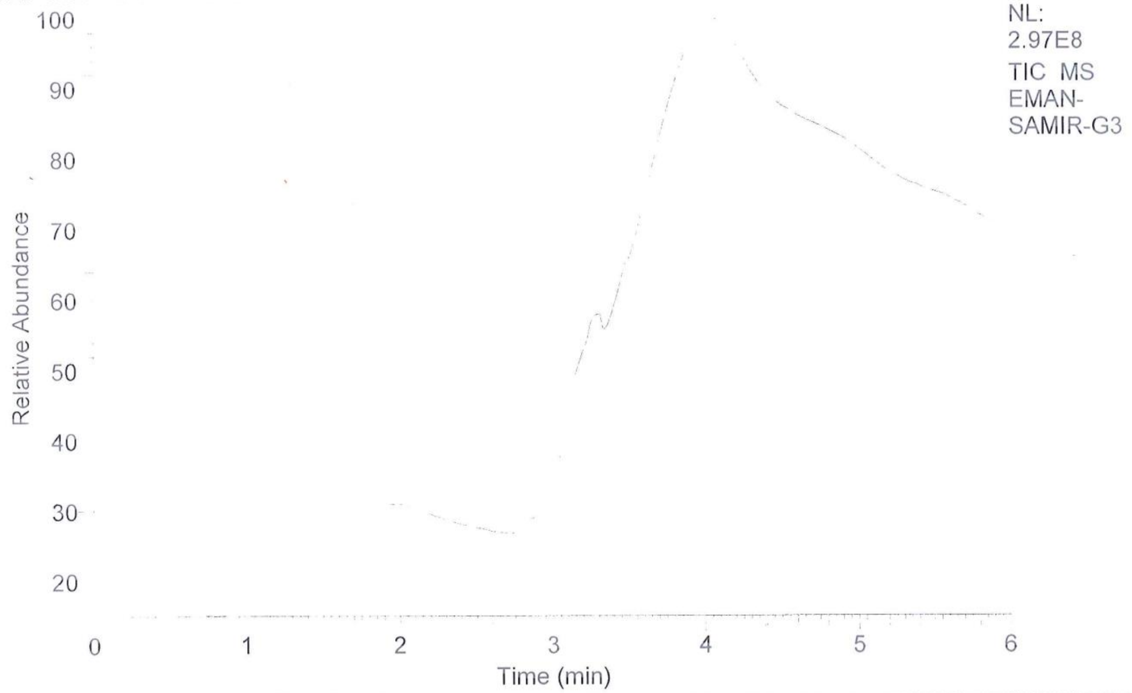

EMAN-SAMIR-G3 #336 RT: 5.64 AV: 1 SB: 6 4.85 , 4.85-4.92 NL: 1.60E6
T: {0,0} + c EI Full ms [40.00-1000.00]

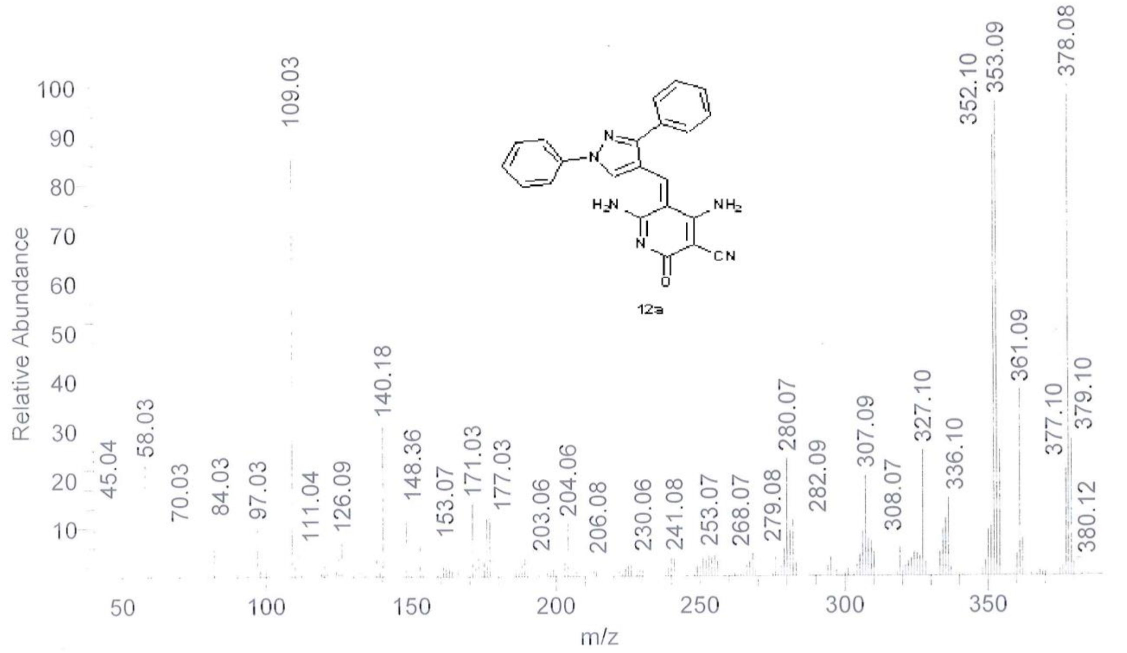

Al-Azhar University
The Regional Center for Mycology & Biotechnology
C:\Xcalibur\data\S\EMAN-SAMIR-G6

2/15/2016 1:43:58 PM
C:\Xcalibur\methods\Mass DIP.meth

RT: 0.00 - 6.00 SM: 15B

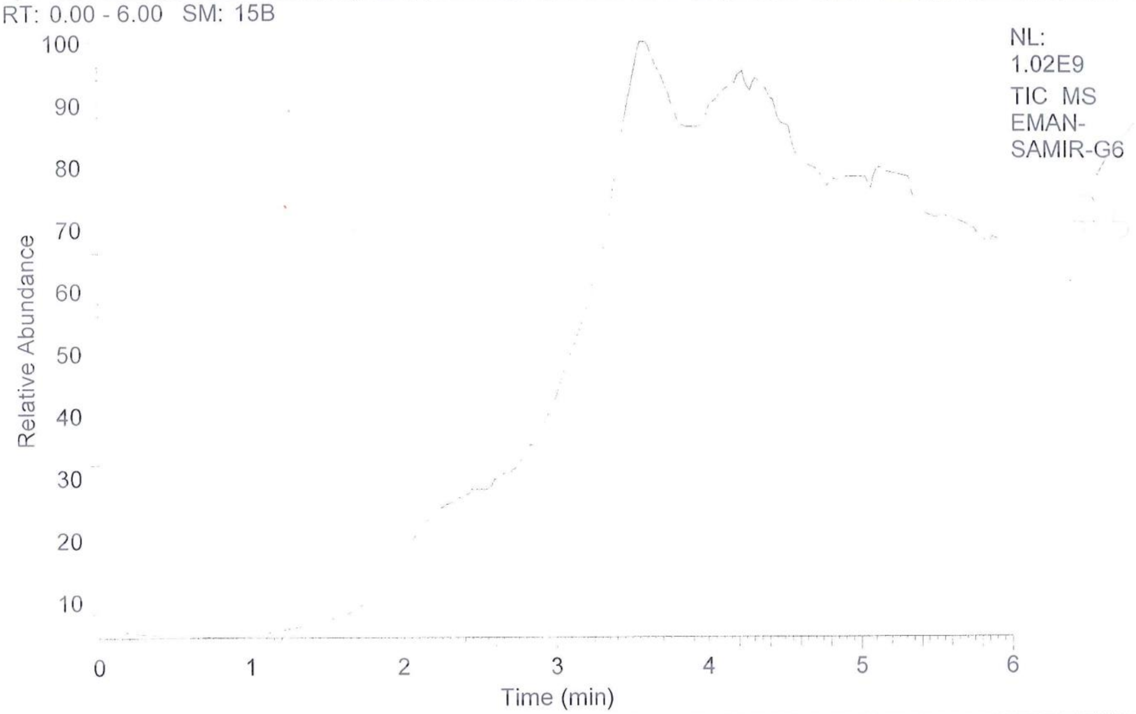

EMAN-SAMIR-G6 #285 RT: 4.79 AV: 1 SB: 2 5.15 , 5.15 NL: 8.12E6
T: {0,0} + c EI Full ms [40.00-1000.00]

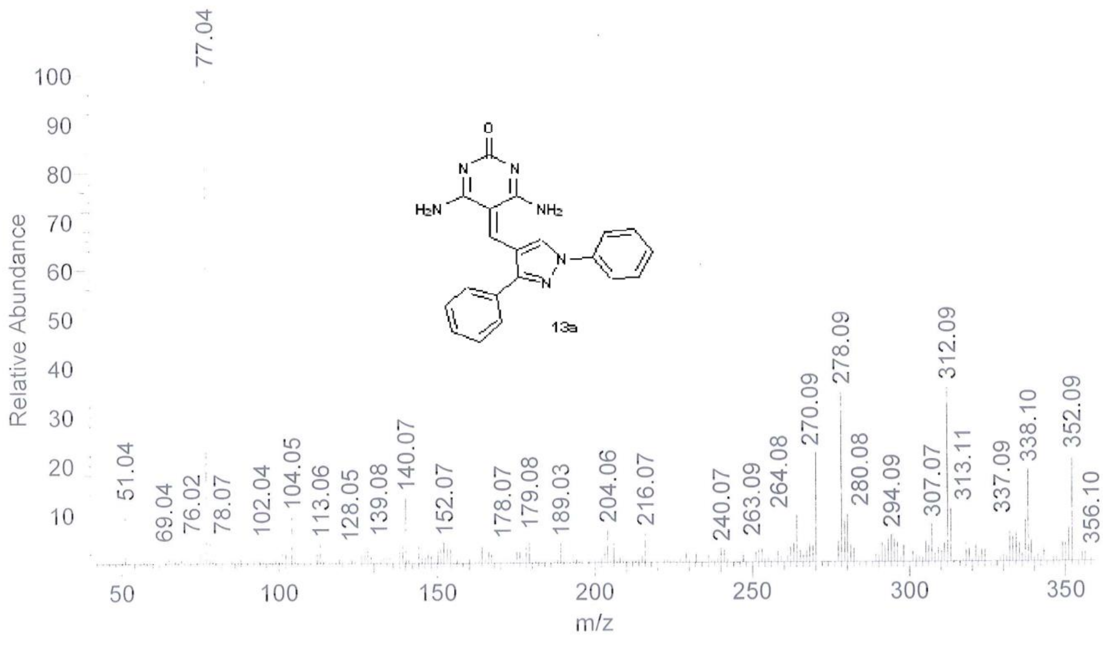

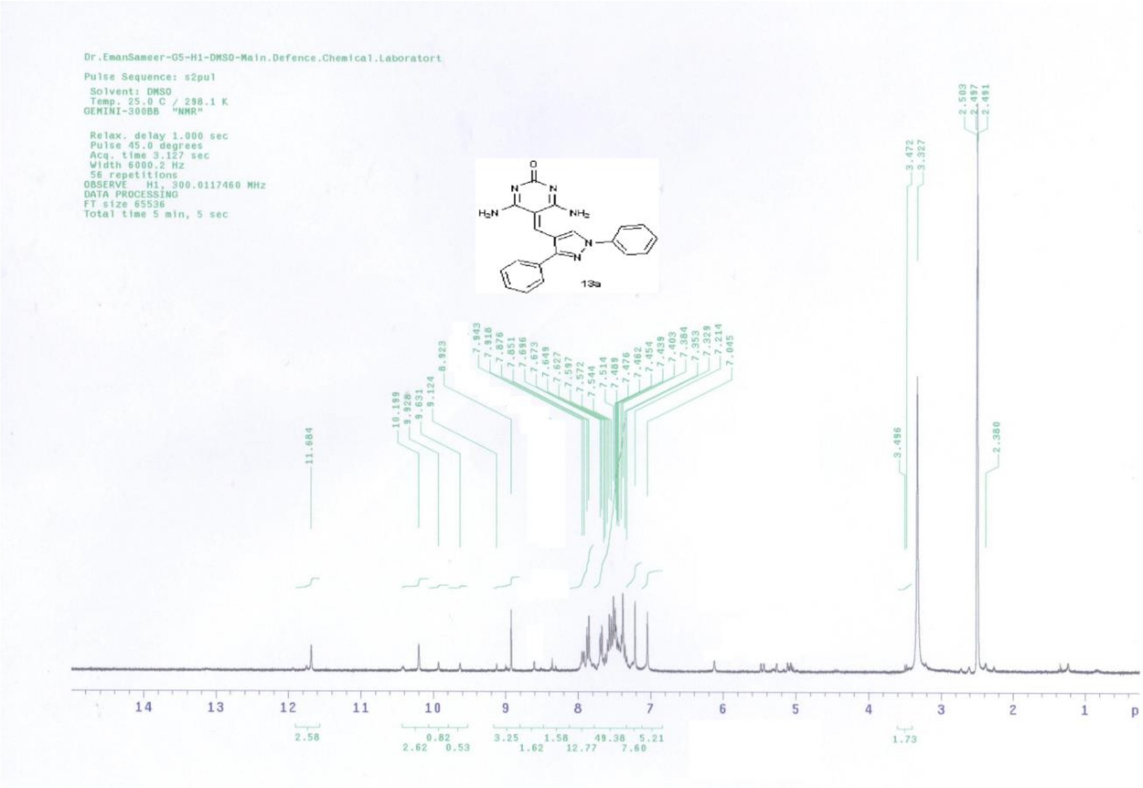
Dr.EmanSameer-G5-H1-DMSO-Main.Defence.Chemical.Laboratort
Pulse Sequence: s2pul
Solvent: DMSO
Temp. 25.0 C / 298.1 K
GEMINI-300BB "NMR"
Relax. delay 1.000 sec
Pulse 45.0 degrees
Acq. time 3.127 sec
Width 6000.2 Hz
56 repetitions
OBSERVE H1, 300.0117460 MHz
DATA PROCESSING
FT size 65536
Total time 5 min, 5 sec
13a
11.684
10.199
9.928
9.631
9.124
8.923
3.496
3.472
3.327
2.503
2.497
2.491
2.380
2.58
2.62
0.82
0.53
3.25
1.62
1.58
12.77
49.38
7.60
5.21
1.73

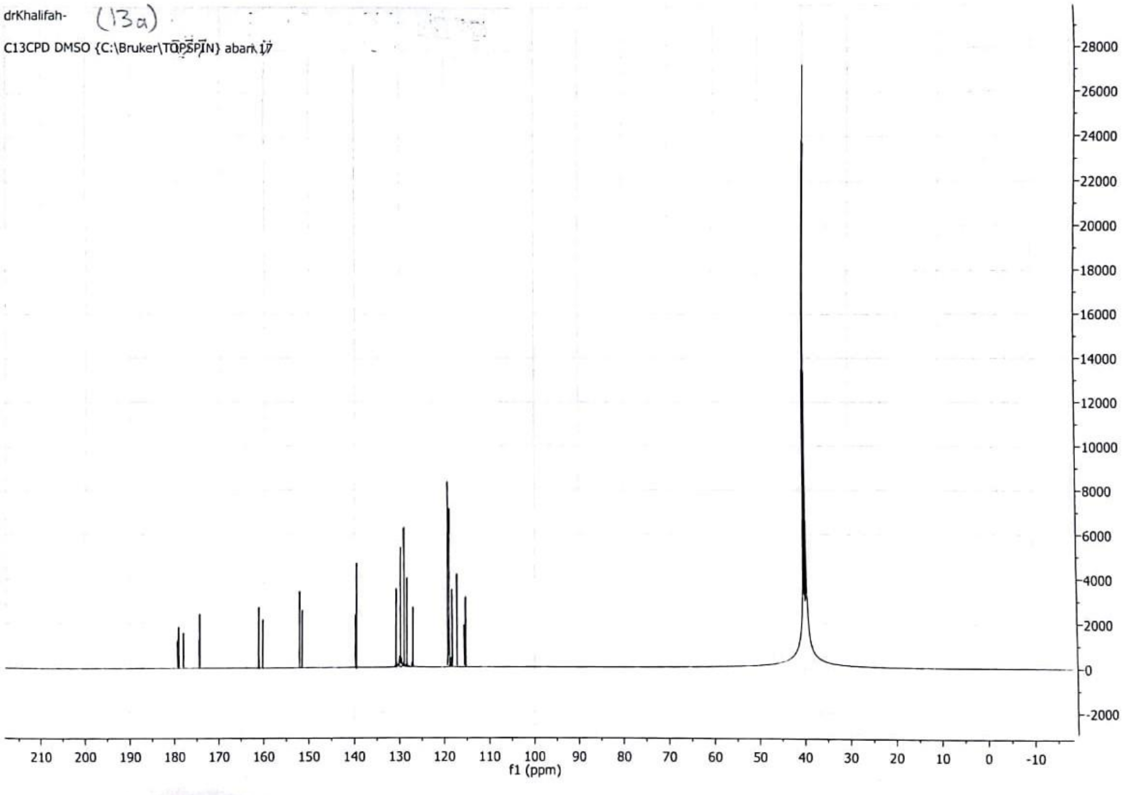
drKhalifah- (13a)
C13CPD DMSO {C:\Bruker\TOPSPIN} abar 17
f1 (ppm)

Al-Azhar University
The Regional Center for Mycology & Biotechnology
C:\Xcalibur\data\S\EMAN-SAMIR-G6

2/15/2016 1:43:58 PM
C:\Xcalibur\methods\Mass DIP.meth

RT: 0.00 - 6.00 SM: 15B

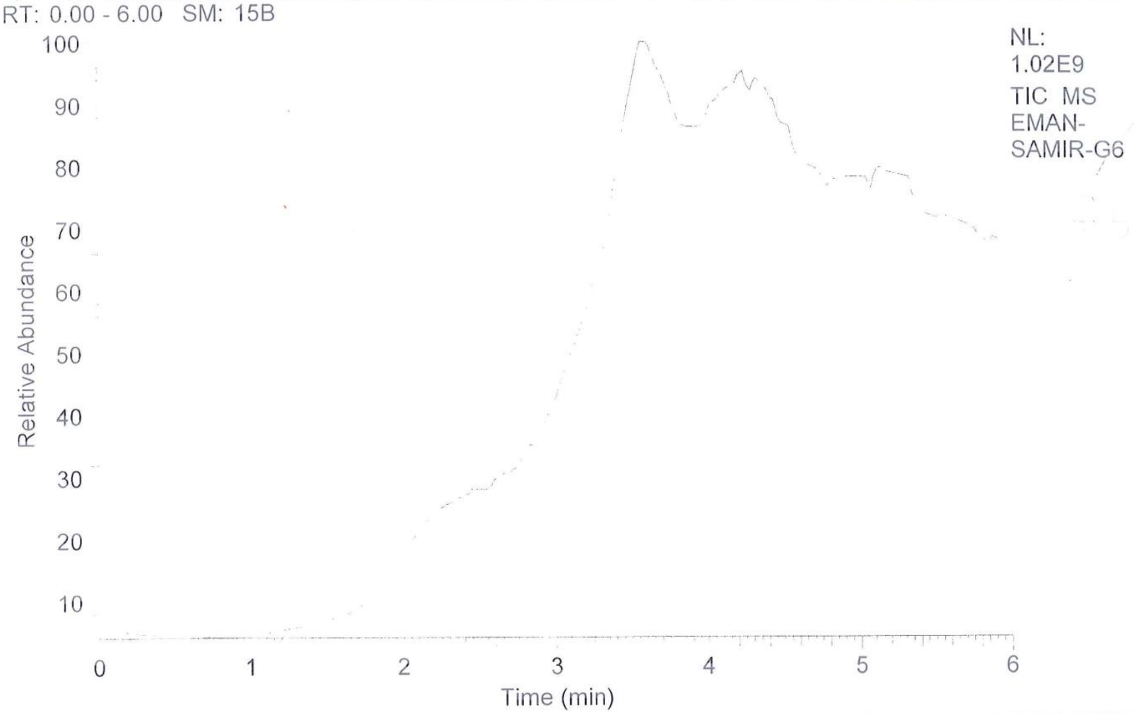

EMAN-SAMIR-G6 #285 RT: 4.79 AV: 1 SB: 2 5.15 , 5.15 NL: 8.12E6
T: {0,0} + c EI Full ms [40.00-1000.00]

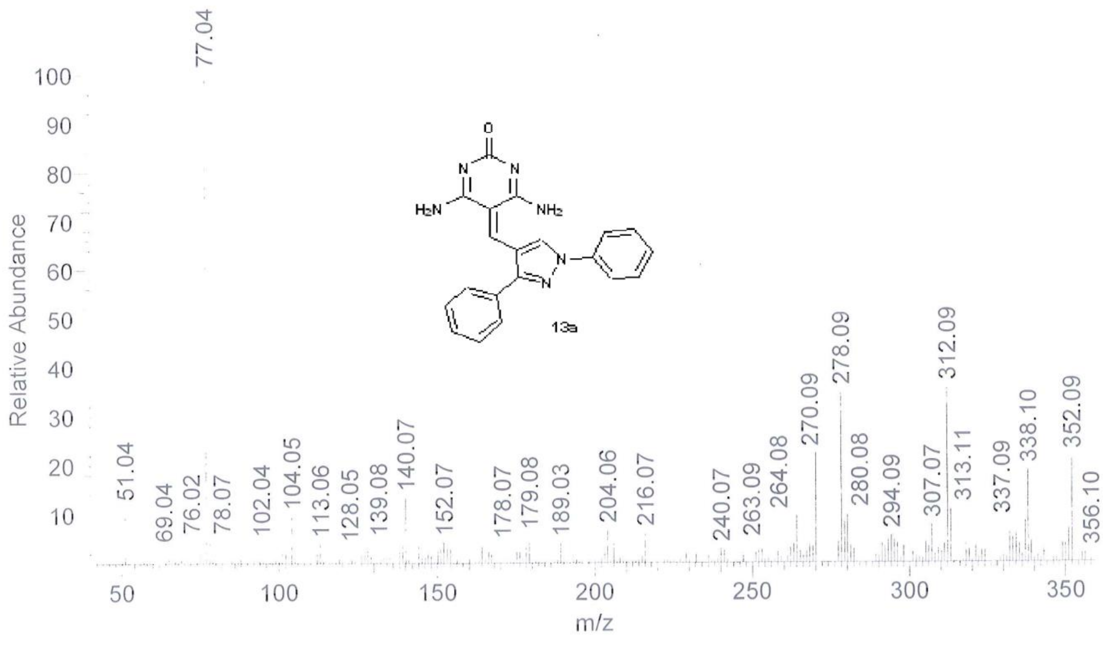

Peak Find - Memory-156

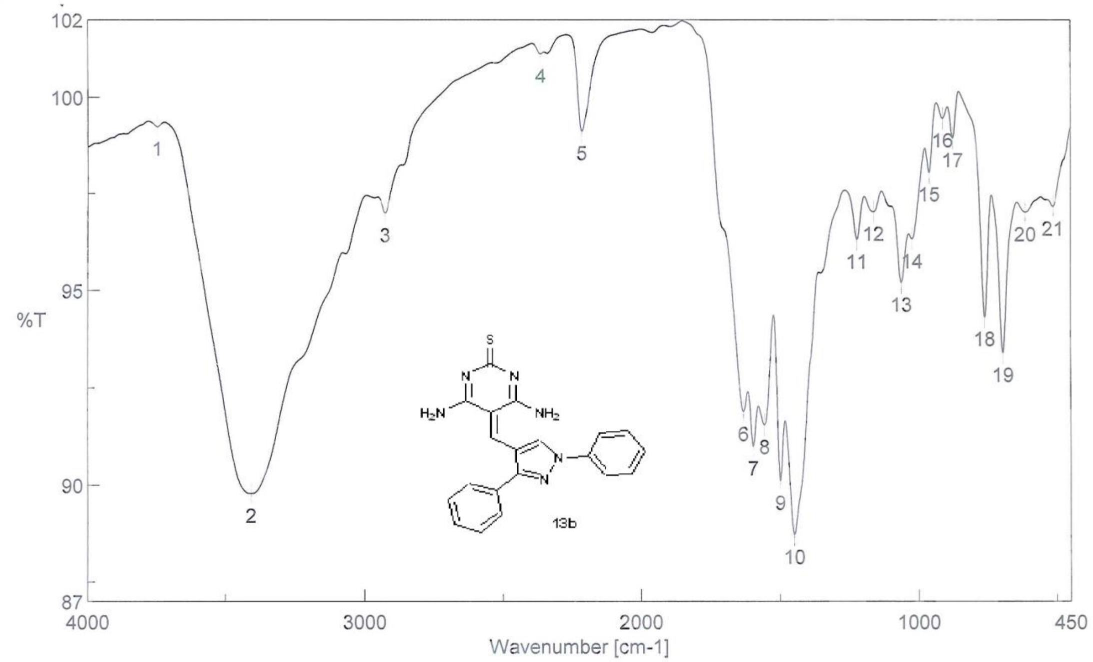

[Comments]

Sample name	G6
Comment	21/4/2016
User	IR
Division	IR
Company	MAC

[Result of Peak Picking]

No.	Position	Intensity	No.	Position	Intensity	No.	Position	Intensity
1	3747.98	99.2234	2	3408.57	89.7747	3	2926.45	96.9738
4	2362.37	101.114	5	2212.92	99.098	6	1631.48	91.8778
7	1596.77	90.9907	8	1556.27	91.54	9	1499.38	90.1032
10	1449.24	88.7124	11	1222.65	96.2909	12	1162.87	97.0044
13	1061.62	95.1876	14	1023.05	96.3014	15	961.341	98.0277
16	912.165	99.4294	17	875.524	98.9146	18	759.816	94.3067
19	694.248	93.3935	20	613.252	96.9949	21	512.972	97.1398

Micro Analytical Center
مركز التحاليل الدقيقة
جامعة القاهرة

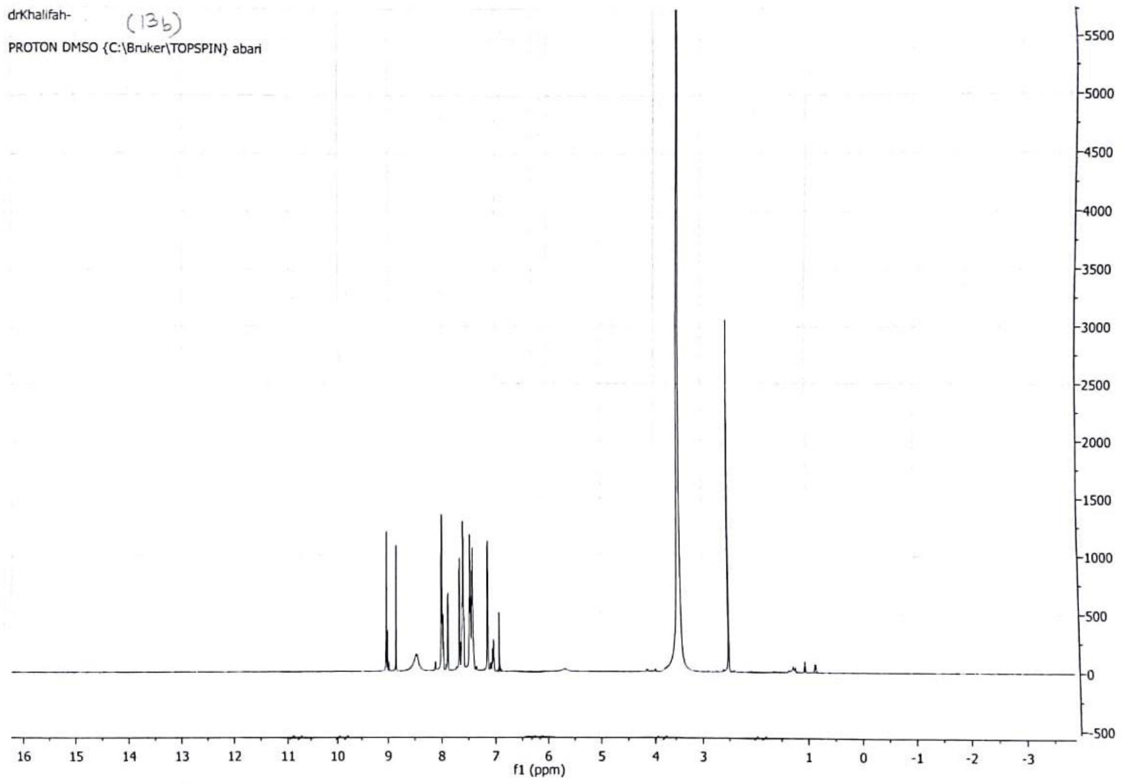
drKhalifah-
(13b)
PROTON DMSO {C:\Bruker\TOPSPIN} abari
f1 (ppm)

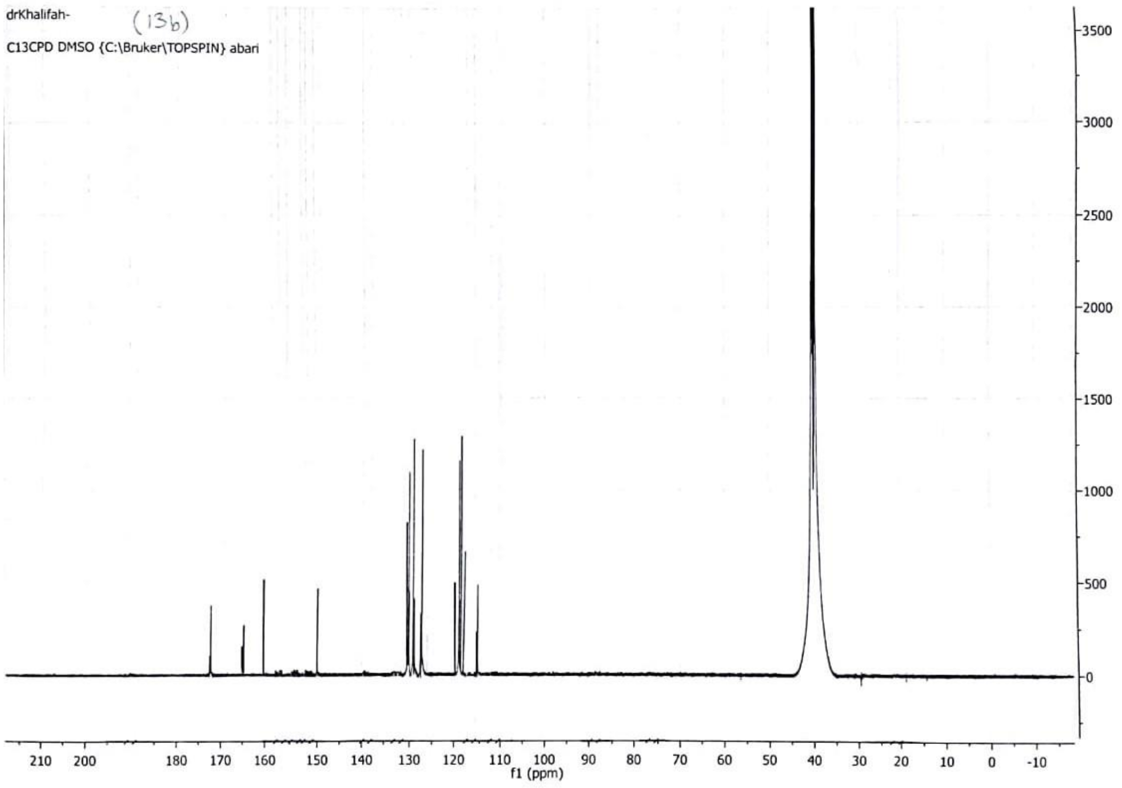
drKhalifah-
(13b)
C13CPD DMSO {C:\Bruker\TOPSPIN} abari
f1 (ppm)

Al-Azhar University
The Regional Center for Mycology & Biotechnology
C:\Xcalibur\data\S\EMAN-SAMIR-G5

2/16/2016 9:40:01 AM
C:\Xcalibur\methods\Mass DIP.meth

RT: 0.00 - 6.00 SM: 15B

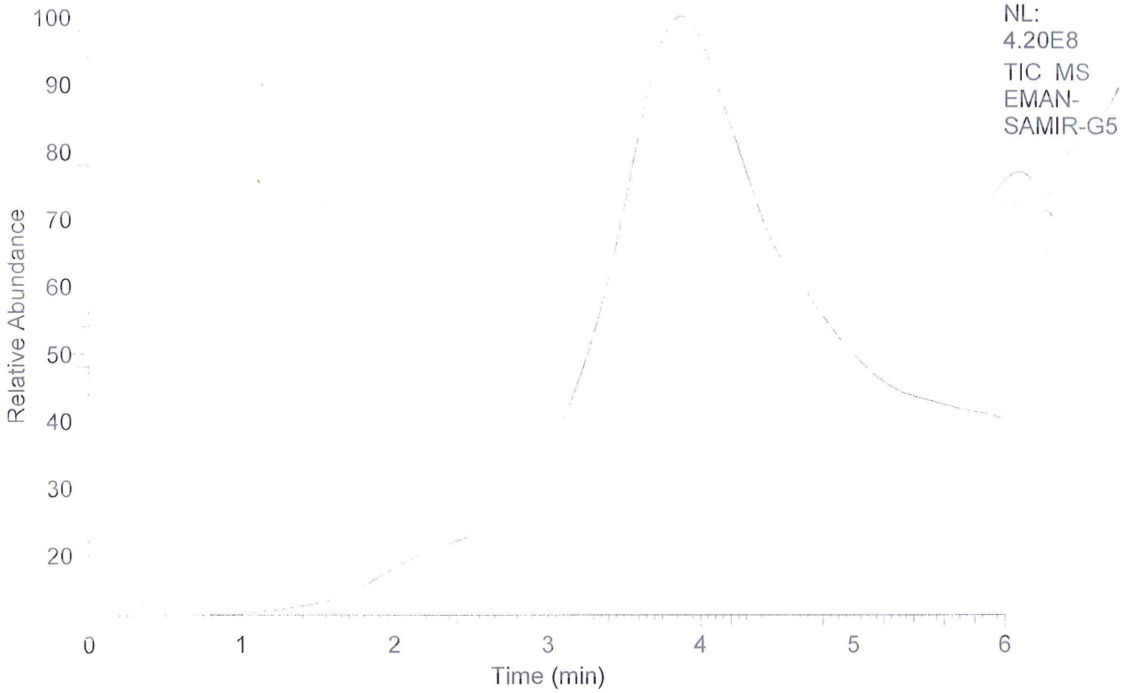

EMAN-SAMIR-G5 #253 RT: 4.25 AV: 1 SB: 6 4.15 , 3.45-3.51 NL: 5.37E6
T: {0,0} + c EI Full ms [40.00-1000.00]

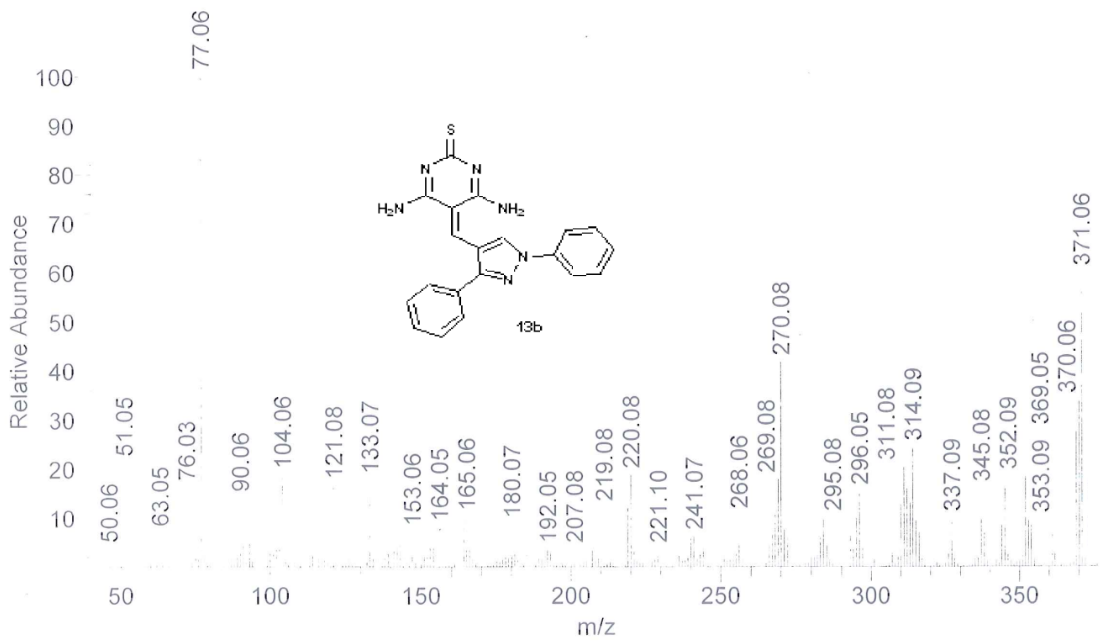

Supplement: Supplementary file 1 [file molecules-23-03074-s001.pdf]
